# Multi-Target Analysis and Design of Mitochondrial Metabolism

**DOI:** 10.1371/journal.pone.0133825

**Published:** 2015-09-16

**Authors:** Claudio Angione, Jole Costanza, Giovanni Carapezza, Pietro Lió, Giuseppe Nicosia

**Affiliations:** 1 Computer Laboratory-University of Cambridge, Cambridge, United Kingdom; 2 Center for Genomic Science of IIT@SEMM, Istituto Italiano di Tecnologia, Milan, Italy; 3 Department of Mathematics and Computer Science-University of Catania, Catania, Italy; Johns Hopkins University, UNITED STATES

## Abstract

Analyzing and optimizing biological models is often identified as a research priority in biomedical engineering. An important feature of a model should be the ability to find the best condition in which an organism has to be grown in order to reach specific optimal output values chosen by the researcher. In this work, we take into account a mitochondrial model analyzed with flux-balance analysis. The optimal design and assessment of these models is achieved through single- and/or multi-objective optimization techniques driven by epsilon-dominance and identifiability analysis. Our optimization algorithm searches for the values of the flux rates that optimize multiple cellular functions simultaneously. The optimization of the fluxes of the metabolic network includes not only input fluxes, but also internal fluxes. A faster convergence process with robust candidate solutions is permitted by a relaxed Pareto dominance, regulating the granularity of the approximation of the desired Pareto front. We find that the maximum ATP production is linked to a total consumption of NADH, and reaching the maximum amount of NADH leads to an increasing request of NADH from the external environment. Furthermore, the identifiability analysis characterizes the type and the stage of three monogenic diseases. Finally, we propose a new methodology to extend any constraint-based model using protein abundances.

## 1 Introduction

The analysis of models and the automated design of metabolic networks and synthetic pathways are key features for investigating biochemical systems. To this end, several mathematical model approaches have been designed, based on ordinary differential expression, stochastic methods, master equations or on algebraic equations [1]. More recently, algorithms and computational methods have been implemented to perform their numerical simulation. This led to biological models that researchers used to build gene regulatory networks of cells under specific experimental conditions [2], to inferring the metabolic flux behavior in single cells for synthetic purposes [3], and to pharmacokinetics/pharmacodynamics studies [4].

**Fig 1:**
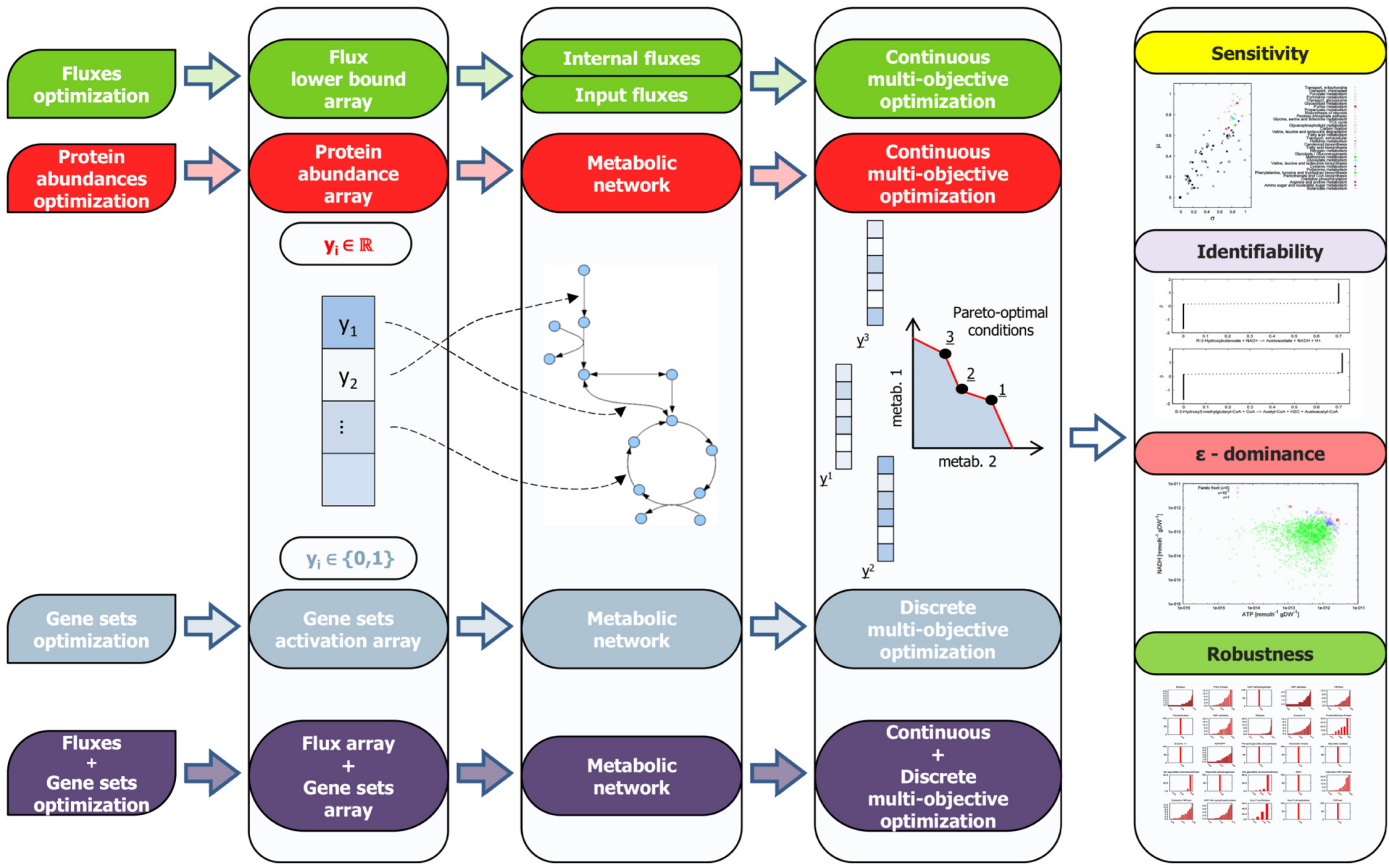
The BioCAD framework is able to perform multi-objective optimization on gene sets and protein abundances. The optimization can be applied to the Boolean arrays of gene sets (simulating an on/off condition), to the real-valued fluxes, or to the real-valued arrays representing protein abundances. In both cases, we seek for the Pareto-optimal arrays to simultaneously optimize two or more objective functions. The optimization is augmented with sensitivity, identifiability, robustness and *ϵ*-dominance analysis. The sensitivity analysis quantifies the importance of the input variables in the model, the identifiability analysis infers functional relations between them, the robustness is used in combination with the sensitivity and quantifies if a solution is reachable even if small perturbations are applied to the system, while the *ϵ*-dominance analysis identifies sub-optimal points.

In this study, we propose the BioCAD framework, which integrates different computational tools able to analyze and query biological models. In particular, it integrates the Pareto optimal principle, the *ϵ*-dominance analysis and the identifiability analysis, with the aim of designing robust metabolic networks that perform specific tasks. Since the models considered in BioCAD represent biological processes, the tasks here reported are biological functions, such as the energy yield of a cell. The framework is general-purpose, and the design acts on genes, reactions, enzymes and metabolites (Fig 1).

In the work by Costanza et al. [5], the authors propose a technique for robust design of microbial strains by using multi-objective optimization techniques [6]. Given two or more conflicting objectives in a system, the Pareto front is the set of trade-off points that are “non-dominated”, i.e., they are such that an increase of one objective comes at the cost of a decrease in the other objective. In a pre-processing step, models are analyzed by means of sensitivity analysis methods. In a post-processing step, each Pareto front achieved by the multi-objective optimization [6] is tested by the robustness analysis [7] in order to evaluate the fragility of each Pareto point. We remark that an effective framework for analysis of metabolic networks would include the following analyses: evolutionary many-objective optimization, robustness analysis, *ϵ*-dominance analysis, sensitivity and identifiability analysis [8].

Our BioCAD pipeline is able to tackle biological problems in an automated way. More specifically, it is able to analyze a metabolic network and design an optimized version of the same network. In the BioCAD framework, we present here a new optimization method called optBioCAD, a stochastic general-purpose optimization algorithm able to perform single and multi-objective optimization. optBioCAD searches for optimal (i) genetic strategies, (ii) metabolites and enzyme concentrations, and (iii) flux rates by using combinatorial/continuous optimization. The *ϵ*-dominance Pareto front analysis extends the search space of solutions, therefore revealing other suitable (suboptimal) points, while the identifiability analysis finds functional relations among decision variables. The source code of our pipeline is in S1 Code. The novelty of the present work lies also in the Protein-Abundance Design through Multi-objective Optimization (PADMO) algorithm, able to integrate protein abundances in genome-scale metabolic networks while ensuring flux balance and optimization. The use of gene expression and protein abundance in computer-aided-design can help to build highly functional and robust engineered strains with reduced times and costs [9].

As introduced above, the energy yield can be considered an interesting point to analyze, since the metabolism of a cell is highly correlated with the energy production and utilization. Mitochondria are organelles in eukaryotic cells and play a key role in the cell. First, they are responsible for the energy productivity, since they synthesize adenosine triphosphate (ATP), the chemical energy in the cell. Second, the mitochondrion is the site of carbohydrates metabolism, fatty acid oxidation and urea cycle. Mitochondria are implied in many other important processes, such as the regulation of calcium homeostasis and other inorganic ions [10, 11], cellular differentiation, cell death (apoptosis) [12], as well as the control of the cell cycle and cell growth [13]. Mitochondria have been also found responsible for several human diseases, including mitochondrial disorders [14], cardiac dysfunction [15], and type 2 diabetes [16]. For the reasons discussed above, we investigate two mitochondrial models [17, 18].

The first model we take into account is the mitochondrial network by Smith et al. [17], composed of 423 reactions (including transformation reactions and transport reactions between compartments and those between internal and external environment) and 228 metabolites. Here, the system is described by considering a steady state for all the metabolites, and solved through flux balance analysis (FBA) [19].

FBA is a modelling approach to simulate and investigate genome-scale metabolic networks [3]. FBA is able to handle large networks and estimate the value of the metabolic fluxes through the network when an objective function is defined (e.g., the growth rate of a cell or its energy yield). The network can be thought as a graph, where each edge represents a reaction and the metabolic flux through the reaction. The direction of each edge is linked to the reversibility or irreversibility of the corresponding enzymatic/transport/exchange reaction. Moreover, the vertices represent the substrates or the products of each reaction. The output of the network (the flux distribution) represents the phenotypic state of the biological system and depends on the topology of the network and on the input, i.e., the uptake rates. From a computational standpoint, the time needed to solve the network is remarkably short if compared, for instance, to methods used to solve ordinary differential equations. Furthermore, FBA does not involve kinetic parameters and is not affected by computational errors of numerical approximation. As a result, FBA allows handling large networks with a large number of components [19].

In the past, FBA models have been chosen for analysis aimed to synthetic biology. For instance, OptKnock [20] implements a bi-level programming framework, GDLS [21] proposes a local optimization algorithm, and GDMO [5] performs optimization based on genetic algorithms, where bacterial cells are optimized and computationally designed in terms of gene knockouts.

For cross-comparison purposes, we will also consider a second model representing the genome-scale metabolic network of the *Chlamydomonas* algal cell [18]. This model includes the mitochondrial organelle; therefore, we use it to calculate the energy yield of the algal cell by searching optimal gene knockout and uptake rates in the FBA framework. The analysis performed on *Chlamydomonas* is reported in S1 File.

We search for those metabolites that are fundamental for optimizing the energy productivity, i.e., for maximizing ATP and NADH production in the matrix. Our method is able to tackle and compare different systems modeled through FBA, gene-protein-reaction mapping (GPR), systems of ODEs, and also to optimize reaction fluxes and gene sets simultaneously. Fig 1 shows the optimization steps performed by BioCAD (columns 1–4) and the additional analyses available (column 5).

The results we obtain indicate that the maximum ATP production is linked to a total consumption of NADH, probably because NADH is completely used to synthesize ATP through the electron transport chain. On the other hand, reaching the maximum amount of NADH leads also to an increasing request of NADH from the external environment. The optimization process showed also that biomass formation increases when ATP production is stopped. As expected, oxygen is more requested when ATP production is maximized, together with hydroxybutanoate, isocitrate, alpha-D-glucose and citrate metabolites. We finally report that the identifiability analysis can be used to detect and characterize the stage of a metabolic disease, as well as the type of monogenic disorder. More specifically, we highlight that functional relations among chemical reactions depend on the disease investigated and, within the same disease, on its stage.

## 2 Results

### 2.1 Optimization of the mitochondrial FBA model

**Fig 2:**
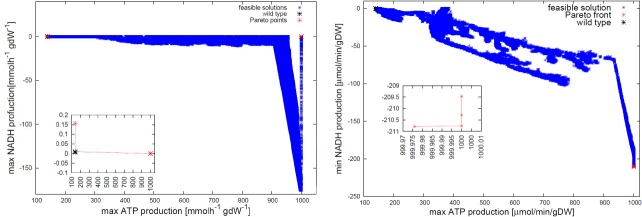
Optimization of the FBA mitochondrial model. (Left) Maximization of ATP and NADH production in the FBA mitochondrial model [17], carried out with 1000 individuals and halted at the 1500^*th*^ generation. We optimized the uptake rate fluxes (73 exchange fluxes) to analyze the energy state of the mitochondrion. In blue the dominated feasible points, in black the wild type conditions, i.e., before optimization and in red the non-dominated Pareto points. Negative flux values represent an uptake rate, while positive values represent a production rate. (Right) Maximization of ATP production and minimization of NADH production.

Using our novel bio-inspired algorithm optBioCAD, we optimize the FBA mitochondrial model by Smith and Robinson [17]. The model contains 423 reactions and 228 metabolites. We specifically optimize energy-related objectives (ATP and NADH production). To measure the availability of NADH, we added an exchange reaction for the metabolite NADH to the external environment through the mitochondrial membrane. Given the common assumptions of flux-balance analysis, such addition does not perturb the dynamics of the metabolic network. Maximizing or minimizing the flux through this reaction represents a FBA-compatible way to require maximum of minimum overall concentration of the NADH metabolite in the mitochondrion. The aim is to find the optimal environment for mitochondria so as to increase their bioenergy yield. The decision variables are the 73 input fluxes. We therefore search for the best values of uptake rate of substrates, which are bounded by a maximum uptake rate of 1000 *μ*mol min^−1^ gDW^−1^ (*DW* stands for dry weight, i.e., the weight of the mitochondrion without water). The optimization finds a single Pareto point that reaches the maximum amount of ATP, without NADH production (Fig 2, left panel). In another optimization experiment, we maximize ATP production and simultaneously minimize NADH production. After 1000 generations of the optimization algorithm, we observe that ATP production grows more rapidly when NADH is consumed more. During the simulation, the algorithm finds many non-dominated points with respect to the previous generations. Instead, in the last generations (after the 900^*th*^ generation), the algorithm finds only four Pareto points, which represent our final results. The results are shown in Fig 2, right panel.

We set the input fluxes of the mitochondrial model as described by Smith and Robinson [17]. With this setting, ATP production is equal to 139.4264 *μ*mol min^−1^ gDW^−1^, while NADH is totally consumed, and the production is equal to 0. At the end of the optimization process, we find that the first non-dominated solution reaches NADH = −140.5484 *μ*mol min^−1^ gDW^−1^ and ATP = 971.0874 *μ*mol min^−1^ gDW^−1^, and the second one reaches NADH = −139.5166 *μ*mol min^−1^ gDW^−1^ and ATP = 971.1778 *μ*mol min^−1^ gDW^−1^. By comparing the initial state with the Pareto optimal state, we remark that ATP increases when the uptake rates linked to (R)-3-hydroxybutanoate, isocitrate, alpha-D-glucose, citrate and oxygen increase. Oxygen is the element that evolves more, and changes from 19.8 *μ*mol min^−1^ gDW^−1^ to 143.17 *μ*mol min^−1^ gDW^−1^. In this case, the optimization does not take into account the limitation of substrates (such as glucose or oxygen) in the biological environment, so we consider this study as an asymptotic analysis for investigating the potential of mitochondria. Recently, different studies reported some experimental validations. For instance, Smith and Robinson [17] found that that when the maximum ATP production is reached (139.43 *μ*mol min^−1^ gDW^−1^ calculated from the FBA model, and 150 *μ*mol min^−1^ gDW^−1^ experimentally measured [22]), both oxygen and glucose uptake rates were at the maximum allowable rate [23, 24]. A negative value of NADH production indicates the NADH uptake rate. We suppose that NADH molecules are used in mitochondria to synthesize ATP molecules. When more NADH molecules are consumed, more ATP molecules are formed.

To give a more in-depth interpretation of our optimization, we choose a minimal set of decision variables, consisting of the following twelve input fluxes: oxygen, arginine, lysine, proline, aspartate, alpha-D-glucose, (R)-3-hydroxybutanoate, isoleucine, valine, hexadecanoic acid, (S)-lactate, HCO3-. Moreover, we change the maximum allowable uptake rate for each variable, that is equal to +33% of the nominal value. In this condition, NADH production does not decrease, while ATP increases and we find ATP = 185.4299 *μ*mol min^−1^ gDW^−1^. Also in this experiment, the oxygen is the variable that increases more (with respect to its nominal value).

#### Optimization of internal fluxes

The optimization of the metabolic reactions in the FBA mitochondrial model has been performed using a genetic algorithm and a novel mutation operator. The genetic algorithm is inspired to NSGA-II with a new mutation heuristic. Since perturbing metabolic reactions in FBA models may lead to unfeasible solutions, we create a mutation operator that takes into account this issue. We introduce two parameters, *C* and *N*. C is the maximum number of fluxes that can be perturbed. If the mutation results in unfeasible solutions due to the constraints operating in the network, the procedure of mutation is repeated until a new child solution is found or a maximum number of *N* trials is reached. In the latter case, the current parent solution is maintained. We have performed the optimization of 229 transformation fluxes and conducted two experiments: (i) the simultaneous maximization of ATP and NADH production and (ii) the simultaneous maximization of ATP production and the minimization of NADH production. For both the experiments we have used a population of 1000 individuals. Each individual contains a vector of 229 values, and each value represents the rate of the corresponding metabolic flux. The mutation operator takes also into account the reversibility of the reactions. Our algorithm has performed the evolution of the population until the 300^th^ generation. The results are shown in Fig S4 in S1 File. For both the experiments, the algorithm finds a set of non-dominated Pareto solutions, showed in red. Dominated and feasible solutions are shown in blue, while the point in black represents the wild type condition, i.e., the condition before the optimization.

Another multi-objective optimization that we take into account for the FBA mitochondrial model involves the production of biomass, ATP and reactive oxygen species (ROS) from complex I. Six reactions in the model represent the formation of the molecules responsible for the growth of the mitochondrion (amino acids, DNA, RNA, lipid, ATP, and heme). In an optimization to maximize ATP and biomass we define as biomass flux the sum of all the biomass fluxes except that of ATP (Fig S5 in S1 File). Conversely, in the optimization of ROS and biomass, to obtain the biomass flux we sum up all the six fluxes (Fig S6 in S1 File).

### 2.2 Characterization of monogenic diseases using identifiability analysis

Studying functional relations in the mitochondrial metabolic network merits further attention due to the possibility to characterize mitochondrial monogenic diseases. Here we consider the FBA model of the mitochondrion by Smith et al. [17]. The identifiability analysis reveals whether a component of a model is identifiable, i.e., uniquely determinable, thus indicating what can (and cannot) be inferred from a model. We choose ATP production as the objective function to evaluate the distribution of fluxes in the network. To reduce the non-identifiability, one should fix the non-identifiable fluxes at an arbitrary value. This does not affect the dynamical properties of the model, since all the variables related to the fixed one will change accordingly.

Our idea is that by performing the Identifiability Analysis (IA) (see Section 4) in healthy, pathological and disease conditions, we can characterize the onset of a disease by looking at the functional relations among fluxes. When taking into account a specific disease, we constrain the reaction responsible for that disease to various values in the range from 0 to the reaction flux under normal conditions [17], and we evaluate the amount of ATP and NADH as outputs of the model. We define a model condition as *disease status* if ATP production is less or equal to 33% of the production under normal conditions, and *inflammation status* if ATP production is less or equal to 66% but more than 33% of the production under normal conditions.

#### Fumarase deficiency

The fumarase deficiency is a monogenic disorder due to the impairment of the fumarate hydratase enzyme, caused by a mutation in the fumarate hydratase gene, which encodes the enzyme. As a result, the fumarate is not converted into malate in the TCA cycle, and therefore a marker for this disease is the presence of fumarate in the urine. Therefore, here we focus on the reaction Fumarate + H_2_O → (S)-Malate. The fumarase flux is constrained to be equal to values equally distributed between 0 and 13.986 *μ*mol min^−1^ gDW^−1^. We first set the flux to be null, and then we increase it by 0.007 *μ*mol min^−1^ gDW^−1^, so as to obtain a 423 × 2000 matrix *V* containing all the fluxes corresponding to fixed fluxes of fumarate. In healthy conditions and when the objective function in FBA is the maximum ATP production, we obtain a fumarase flux of 6.9721 *μ*mol min^−1^ gDW^−1^. In the fumarase deficiency conditions (about 2–3 *μ*mol min^−1^ gDW^−1^), ATP production is reduced until 75% of the maximum value [17]. For the fumarase deficiency, we identify these intervals of the fumarase flux: healthy stage [10,5.69), inflammation stage [5.69,3.69), pathological stage [3.69,0] *μ*mol min^−1^ gDW^−1^ (we adopt the standard notation for half-closed intervals; [*a*, *b*) indicates that *a* is included and *b* is not included).

**Fig 3:**
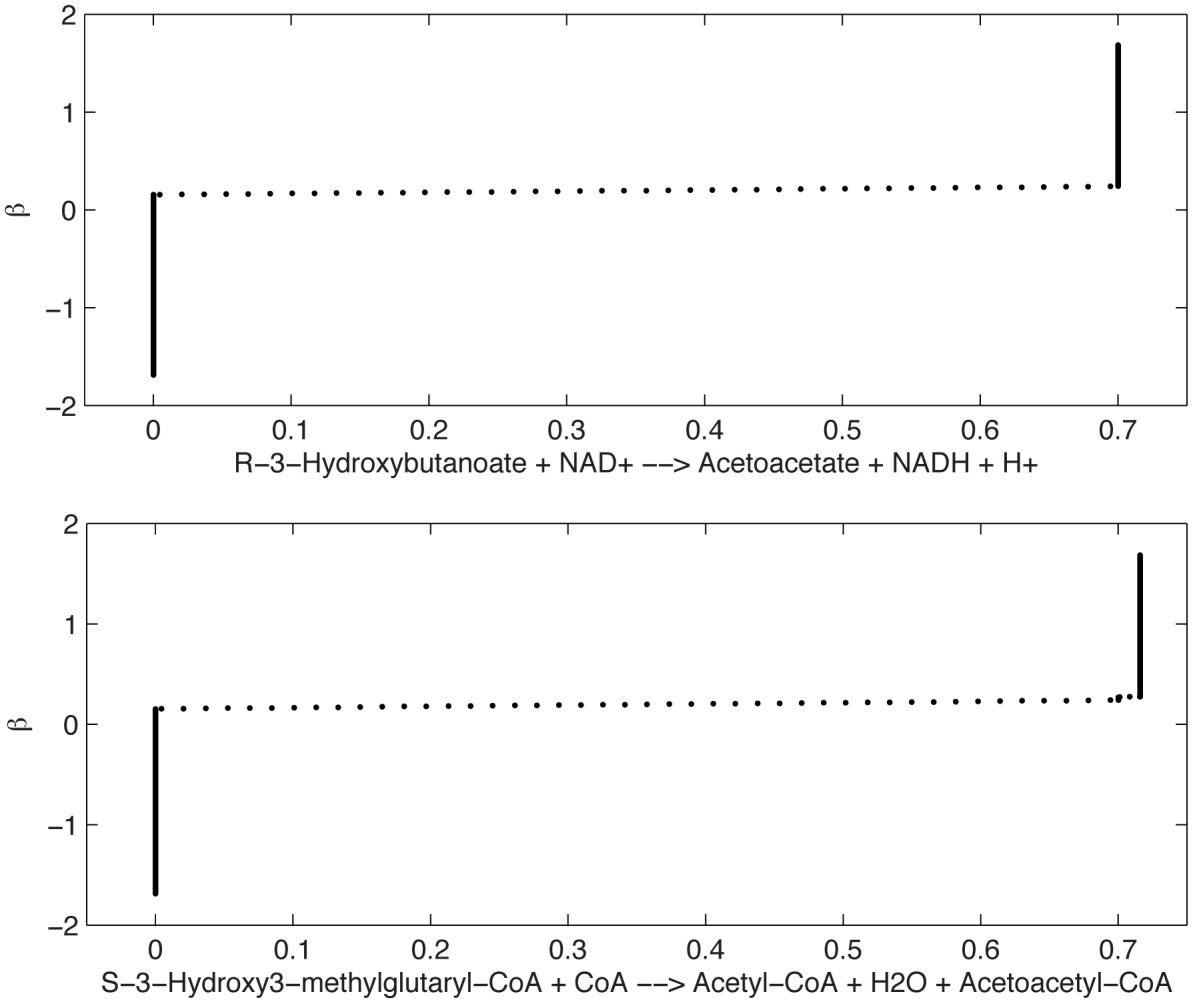
Optimal transformations *β* (*y* axis) found for the two fluxes R01361MM (top) and R01978MM (bottom) (*x* axis) [*μ*mol min^−1^ gDW^−1^] in the mitochondrial FBA model [17]. This plot proves that there is a strong relation between these two fluxes, with slightly different and noisier behavior in the neighborhood of 0 and 0.7.

We first apply the IA to detect global relations between two or more variables, allowing the fumarase flux to span all the interval [0,13.986] *μ*mol min^−1^ gDW^−1^. In the table summarizing the results (Table A in S1 File), the “groups” column indicates the functional relations between variables. For instance, R01361MM (conversion of (R)-3-Hydroxybutanoate and NAD^+^ into acetoacetate, NADH and H^+^) and R01978MM (conversion of (S)-3-Hydroxy-3-methylglutaryl-CoA, and CoA into acetyl-CoA, H_2_O and Acetoacetyl-CoA) are functionally related. In other words, the first reaction (response variable *x*_47_) is strongly related to the second reaction (predictor *x*_62_). (See Table A in S1 File and Table L in S1 FIle for reaction ID and stoichiometry.) In Fig 3 we plot the optimal transformations *β* found for these two reactions (see also Section 4). We note that the transformations are similar to each other, indicating the structural non identifiability of both variables. The functional relation between these two reactions (*x*_47_ and *x*_62_) has been detected by the identifiability analysis applied to both reactions. This is therefore a strong relation, marked by a double asterisk in Table A in S1 File.

In Table B in S1 File, Table D in S1 File, and Table E in S1 File, we show the results of the IA applied to the metabolic network with various values of the fumarase flux. The (*x*_28_, *x*_50_) group is detected both in the healthy and in the pathological stage, but not in the inflammation stage. In the pathological stage only four different functional relations are detected, which means that variables are mostly unrelated to one another.

#### Succinate dehydrogenase deficiency

This disorders affects the mitochondrial complex II, responsible for linking the TCA cycle with the electron transport chain. It is often due to the bi-allelic inactivation of the SDHA gene. The deficiency of succinate dehydrogenase causes encephalomyopathy or tumor formation, and may affect motor and mental skills.

The behavior of ATP as function of the succinate dehydrogenase flux is equivalent to that obtained as function of the fumarase flux [17]. Therefore, we identify three intervals of the succinate dehydrogenase flux: healthy stage [10,5.69), inflammation stage [5.69,3.69), pathological stage [3.69,0] *μ*mol min^−1^ gDW^−1^.

**Fig 4:**
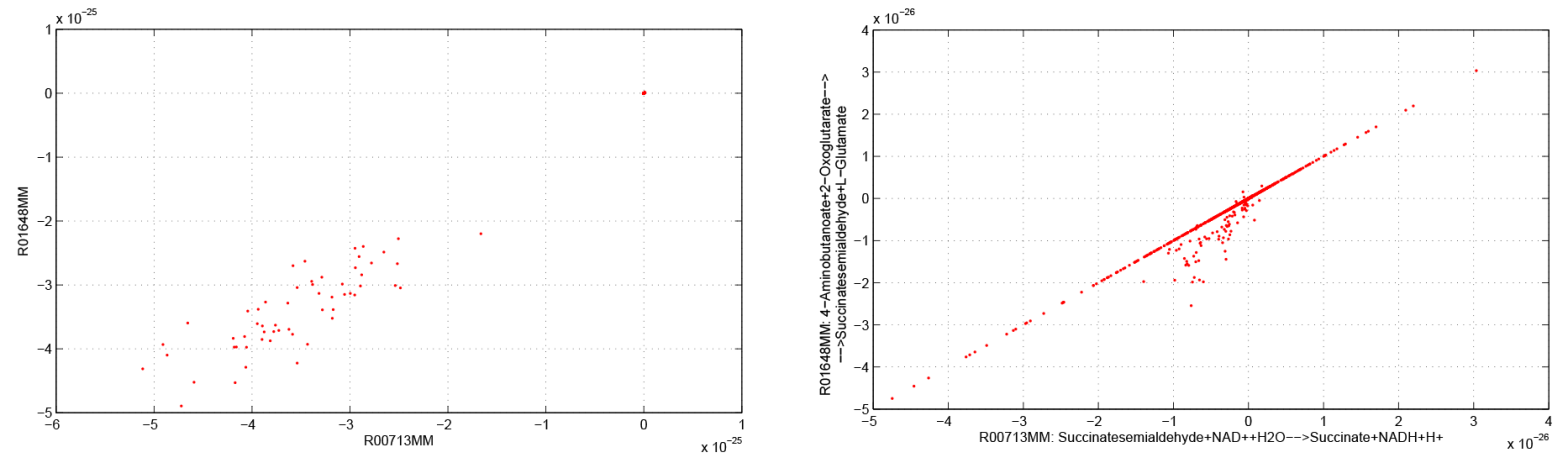
Fumarase deficiency (left) and succinate dehydrogenase deficiency (right) in the healthy stage. Functional relation found for the two fluxes R00713MM and R01648MM (*x* axis) [*μ*mol min^−1^ gDW^−1^] in the mitochondrial FBA model [17]. Reaction stoichiometry is reported in Table L in S1 File. In the same stage of disease, the IA can be used to detect the type of monogenic disorder through the shape of the functional relation.

**Fig 5:**
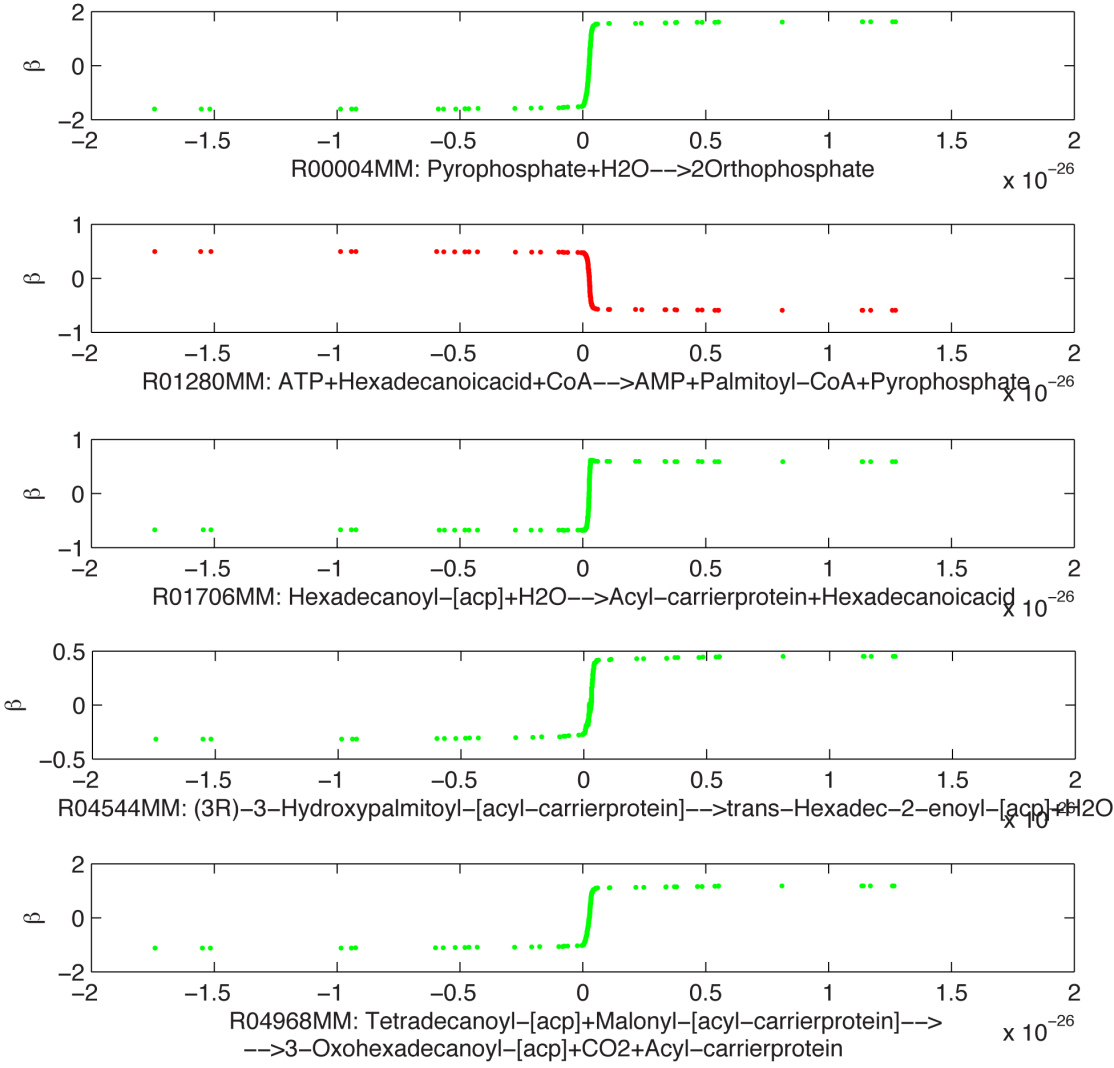
Succinate dehydrogenase deficiency—inflammation stage. Optimal transformations *β* (*y* axis) found for the five fluxes R00004MM, R01280MM, R01706MM, R04544MM, and R04968MM (*x* axis) [*μ*mol min^−1^ gDW^−1^] in the mitochondrial FBA model [17].

In Fig 4 we show a strong functional relation between the two reactions R00713MM and R01648MM in healthy stage (full reaction stoichiometry is reported in Table L in S1 File). By cross-comparing, in the healthy stage, this functional relation under succinate dehydrogenase deficiency with the functional relation under fumarase deficiency referring to the same functional group, we report that the IA can be used to detect the type of monogenic disorder. In fact, the relation between the two variables depends on the disease investigated, although the intervals chosen are equivalent to those of the fumarase deficiency. Functional groups within succinate dehydrogenase deficiency may also include a high number of reactions (Fig 5).

#### *α*-ketoglutarate dehydrogenase deficiency

The *α*-ketoglutarate dehydrogenase enzyme provides protection against cyanide induced convulsions, and decreases mitochondrial damage induced by seizures caused by kainic acid. The corresponding deficiency affects the TCA cycle, and in particular the conversion of oxoglutarate into succinyl-CoA.

**Fig 6:**
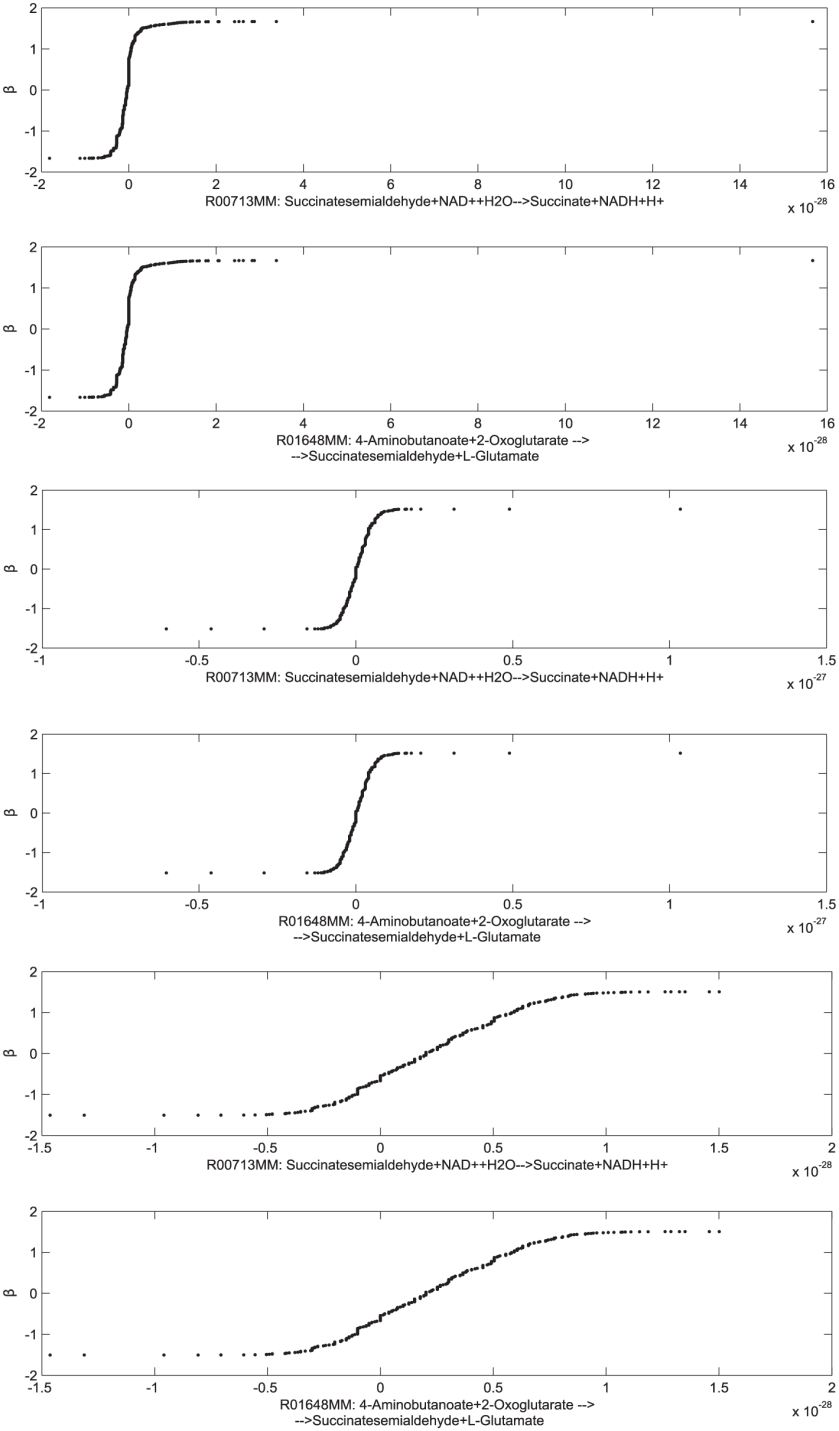
*α*-ketoglutarate dehydrogenase deficiency—healthy stage (top), inflammation stage (middle), pathological stage (bottom). The optimal transformations *β* (*y* axis) have been found for the two fluxes R00713MM and R01648MM (*x* axis) [*μ*mol min^−1^ gDW^−1^] in the mitochondrial FBA model [17].

The intervals identified by plotting ATP as function of *α*-ketoglutarate dehydrogenase flux are as follows: healthy stage [0,8.31), inflammation stage [8.31,10.31), pathological stage [10.31,12] *μ*mol min^−1^ gDW^−1^. Interestingly, the transformations *β* found for the healthy, inflammation and pathological conditions (Fig 6) show that the optimal transformation in the pathological stage has a different shape with respect to the healthy and the inflammation stages. The functional relations associated with these plots are in S1 File.

### 2.3 Optimization of protein abundances for FBA models

Given that protein synthesis is an outcome of expression of the genes that code for protein segments, our idea is to link the values representing protein abundances to the bounds of the flux of the reactions controlled by those proteins. We remark that the FBA models allow the variation of the bounds of each reaction flux. After the perturbation of the range of the reaction fluxes, due to the values of protein abundance given by the optimization algorithm, the actual reaction fluxes are the results of the FBA, simulating the local variability of fluxes. As a result, by perturbing the values of protein abundance through a multi-objective optimization method, desired fluxes can be concurrently increased [25]. In other words, we perform a Protein-Abundance Design through Multi-objective Optimization (PADMO).

Let *y*_*i*_ be the protein abundance of the *i*th protein, responsible for the *i*th reaction of the model. In order to map the protein abundance value into a specific condition of the model, we use the following piecewise multiplicative function (Fig S11 in S1 File):

$$\begin{array}{c} f\left(y_{i}\right)=\{\begin{array}{cc} (1+{|log(y_{i})|)}^{\left(y_{i}-1\right)/|y_{i}-1|} & \text{if}\;y_{i}\in {\mathbb{R}}^{+}\setminus \{1\}\\ 1 & \text{if}\;y_{i}=1 \end{array} \end{array}$$ (1)

In the FBA model, we change the minimum and maximum flux of the *i*th reaction accordingly: $v_{i}^{min}=V_{i}^{min}f(y_{i})$, $v_{i}^{max}=V_{i}^{max}f(y_{i})$, where $V_{i}^{min}$ and $V_{i}^{max}$ are the minimum and maximum flux of the wild-type configuration of the model. The bounds of the reaction fluxes are multiplied by the logarithmic piecewise multiplicative function (Eq 1) so as to avoid that the genetic algorithm underlying the optimization is driven towards high and unfeasible values of protein abundance. Namely, the upper and lower bound of the flux in the FBA model in a specific condition are equal to the wild-type bounds multiplied by *f*. In *y*_*i*_ = 1, the function has a discontinuity of the third kind, removable by imposing *f*(1) = 1. The logarithm function, in a different way, has already been used in other approaches [26], so as to build new objective functions when the argument is the ratio between gene expression values. Regarding *f* as a *f*(*x*) scaling function, the properties $f(x)\vec{x\to 0}0$ and *f*(1) = 1 ensure that GDMO [5] becomes a particular case of PADMO. The steps of PADMO are presented in Fig 1 (red row).

The FBA model can be optimized through a multi-objective evolutionary algorithm. In GDMO [5], each “individual” of the population is represented by a binary variable set representing the knockout strategy of gene sets. Conversely, in PADMO the individuals are arrays of real values, each of which represents the abundance of a protein. Through the function *f*(*x*), these abundances have a continuous effect on the FBA model, rather than only an on/off effect on reactions.

**Fig 7:**
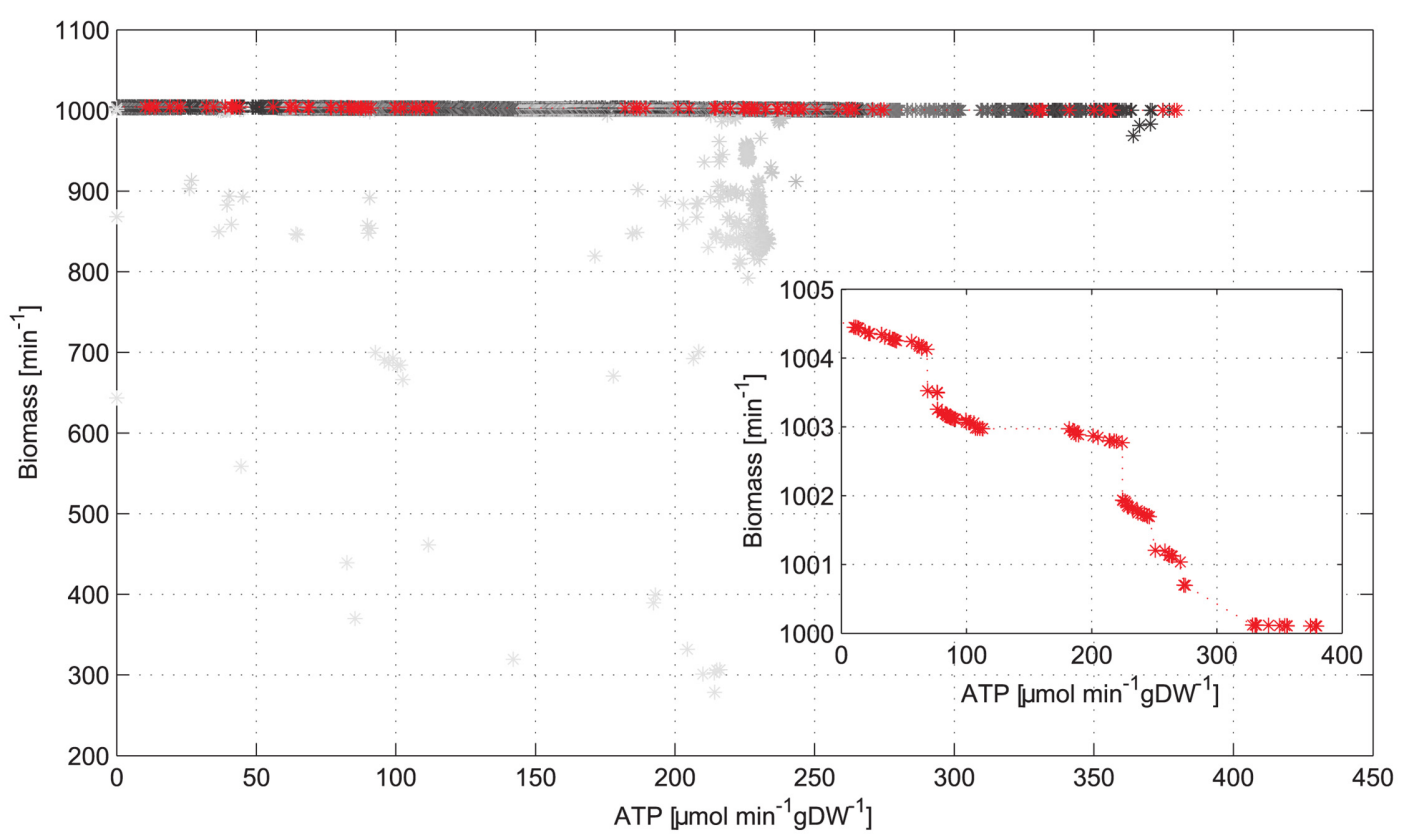
Maximization of ATP and biomass in the mitochondrial FBA model when taking into account the protein abundances as real-valued variables. The red points constitute the Pareto frontier, while the others represent all the feasible points. The points are color coded according to the generation to which they belong. Points from the early generations of the genetic algorithm are colored light grey, while points from the last ones are colored black.

The multi-objective optimization can then be performed with optBioCAD. For each generation of the algorithm, we provide the Pareto optimal solutions, in order to evaluate the evolution of the Pareto front. This loop is repeated until the solutions set does not improve, or until an individual with a desired phenotype is achieved. The number of generations and population are parameters chosen by the researcher. Using this approach, each point of the Pareto front is not merely a specific optimal model in the objective space, but also a protein abundance array representing a specific condition in the variable space. In Fig 7 we show the results of PADMO applied to the maximization of ATP and biomass in the mitochondrial FBA model. We run the model looking for the optimal arrays of protein abundances that maximize both concurrently. Since the model has five biomass reactions other than that of ATP, we define as biomass flux the sum of all the five biomass reaction fluxes.

## 3 Discussion

Mitochondria are the core of cellular metabolism, since they generate ATP molecules. Data from literature demonstrate that mitochondria play a crucial role in neuronal cell survival [27]. Calcium is an important ion inside mitochondria and its concentration is fundamental to regulate functions and acts at several levels during the ATP synthesis. The dysregulation of the mitochondrial Ca^2+^ homeostasis is involved in many pathologies. For example, an accumulation of Ca^2+^ ions in the mitochondrial space can lead to an increased generation of ROS (reactive oxygen species) that alters the permeability of the inner membrane leading the cell to apoptosis. Additionally, the dysregulation of the Ca^2+^ homeostasis is involved in neurodegenerative diseases [11]. ATP metabolism, Ca^2+^ homeostasis, NAD+, NADH and ROS are key players in the cellular mechanisms, and their alteration can lead to the cell death. Diseases like Parkinson [28], Alzheimer [29] and Amyotrophic Lateral Sclerosis [30] have a common point: mitochondrial dysfunction occurs prior to the onset of the pathology symptoms.

In this study, we analyzed how the genetic and the energy-converting pathways of mitochondria can be explored using multi-objective optimization, sensitivity, identifiability and *ϵ*-dominance. These techniques are framed in a unique pipeline applicable to a variety of conditions and organisms. As well as optimizing simultaneously two or more outputs of a model, the pipeline can also provide interesting insights into clusters of chemical reaction networks, which are often found in the cell and reflect the presence of different pathways with different responses to external or internal perturbations. Indeed, in addition to optimizing input and output fluxes, we defined a strategy to determine the role of the internal fluxes in the mitochondrial FBA network, evaluating the response of the network to changes of their rate.

The multi-objective optimization algorithm we propose in this manuscript, named optBioCAD, allows for a better coverage of the objective space. More specifically, it has been designed to generate diversity among the solutions during the convergence process, and to use various perturbation operators to ensure both a local and a global search of optimal solutions. Our pipeline is suitable for comparing the structure of the metabolism through optimization and associated measures for any model taken as a “black-box” input.

For instance, by comparing Fig 2 and Fig S1 in S1 File, the analysis of the Pareto front highlights that the NADH production is flexible in the mitochondrial model by Smith et al., but not in the algal model, which instead allows higher values of ATP production. Furthermore, the algal model is able to better highlight the changes in the metabolism with respect to the wild type condition. On the other hand, the multi-objective optimization of the mitochondrial model finds many Pareto-optimal and sub-optimal points, while there are only few optimal points for each optimization run on the algal metabolism.

The sensitivity analysis has detected the variables playing the major role on the output of the model. Specifically, the PoSA algorithm assesses the sensitivity of each pathway, rather than focusing only on a single reaction or component of the model. Most importantly, PoSA is also able to handle Boolean inputs [5]. Conversely, the identifiability analysis has detected the functional groups of variables. The elements of a functional group are variables functionally related with each other, which cannot be determined unambiguously. In our work, the identifiability analysis is applied to the input variables of each model, considering only the values in the variable space that correspond to the Pareto optimal points in the objective space. The *ϵ*-dominance analysis is performed to investigate the neighborhood of the suitable Pareto-optimal designs (see S1 File).

The decision variables of our multi-objective optimization algorithm can also be the protein abundance values. Each point of the Pareto front represents a different strain or the same strain responding differently to different sets of environmental conditions. By refining our algorithm with the possibility of modifying real-valued protein abundances rather than binary knockout arrays, we provide a new method to optimize simultaneously two or more outputs of the model by finding the best protein abundance array. Most notably, this may permit to determine the best environmental condition in which an organism has to be grown in order to reach specific optimal output values from a range of objective functions chosen by the researcher. This method is particularly suitable for bacteria [31], when different environmental conditions cause differences in gene expression arrays and therefore in the protein abundances, whose effect can be analyzed in the FBA model using our novel PADMO algorithm. Interestingly, our approach can serve as support for the study of gene expression also in mitochondria. For instance, in studies linking gene expression data and mitochondrial disease [32], our method can be used to predict the effect of changes in gene expression due to the specific disease under investigation. In particular, using our algorithm to implement the genetic and genomic status of mitochondria can help infer biomarkers for diseases diagnoses and prognoses [33].

The interplay between these techniques in our general-purpose framework can be exploited not merely to reach the optimal and suboptimal configuration for a model in a single-objective and multi-objective fashion, but also to conduct tentative analyses on the variables and components of any model including ODEs, DAEs, FBA and GPR mapping. In fact, our framework is also suitable for general-purpose and comparative analysis, enabling to investigate and cross-compare not merely any biological pathway modeled with ordinary differential equations, differential algebraic equations, flux balance analysis and gene-protein-reaction mapping, but also models in which fluxes and gene sets need to be optimized concurrently. Striking applications of our pipeline could be in the field of metabolic engineering of a whole biological network. In particular, we propose a general and automated way to optimize biological models and assess their optimal solutions. In a network of organisms interacting with each other, where each model becomes a submodel of the whole network model, our approach can be used for analyzing all the network. The results given by the framework applied to each submodel can be easily integrated, being output of the same pipeline, thus allowing a convergence of different modelling techniques [5, 34, 35].

In each optimization procedure we have considered a single organelle, while in the cells there are usually many compartments, each of which contains an organelle. Compartments, also referred to as submodules, may differ for their activity depending on their location in the cell. In a module of interacting organelles, most of the reactions involve more than one compartment. Indeed, any kind of circuit can be split into submodules to increase its efficiency. An appropriate approach towards whole-cell analysis would therefore be to build a Pareto front where each objective belongs to a different compartment [36, 37], linking compartments with a set of delay differential equations (DDEs) to account for events that depend on the state of the system at an earlier time (e.g., diffusion processes or maturation events). In this way, we could envisage our framework in a larger common pipeline to investigate not merely biological circuits, but also human body monitoring techniques, biosensors design, as well as a possible integration with microelectronics, e.g., CMOS biomicrosystems.

## 4 Methods

### 4.1 Searching for optimal trade-offs: the Pareto front

Standard optimization routines in computational biology are concerned with the maximization of a single objective, usually the growth rate. However, there is increasing evidence that cells need to optimize multiple, often conflicting, objectives. When two tasks are in contrast with each other, there is no phenotype that can be optimal at all of them. However, multi-objective optimization [6] allows searching for all the trade-off solutions for both tasks.

We exploit the concept of multi-objective Pareto optimality to maximize or minimize two or more desired metabolites in a model, thus obtaining new in silico synthetic strains, which are optimal in many parameters or variables simultaneously.

Let us assume to have *r* objective functions *f*_1_, …, *f*_*r*_ to optimize. The problem of multi-objective optimization can be formalized as finding a solution x* that optimizes the vector function

$$\begin{array}{c} f\left(x\right)=(f_{1}\left(x\right),f_{2}\left(x\right),...,f_{r}\left(x\right)), \end{array}$$ (2)

where *x* is the variable in the search space.

The solution of a multi-objective problem is a set of points called Pareto optimal solutions or *Pareto front*. A point y* in the solution space is said to be Pareto optimal if there does not exist a point *y* such that *f*(*y*) *dominates* *f*(y*). Formally, if we consider the maximization problem, y* is Pareto optimal if ∄ *y* s.t. *f*_*i*_(*y*) > *f*_*i*_(y*), ∀*i* = 1, …, *r*, where *f* is the vector of *r* objective functions to maximize in the objective space. Without loss of generality, we have assumed that all the functions have to be maximized (note that minimizing a function *f*_*i*_ can be thought of as maximizing −*f*_*i*_). The objective functions *f*_*i*_ are usually in conflict with each other, and therefore we use the Pareto-front concept to find the set of designs that represent the best trade-off between two or more requirements. From a biological perspective, the Pareto front is the set of all the phenotypes that remain after eliminating all the feasible phenotypes dominated on all tasks [38].

### 4.2 OptBioCAD: a stochastic general-purpose optimization algorithm

In order to tackle multi-objective optimization problems, we design a novel algorithm, called optBioCAD, which belongs to the class of the well-known evolutionary multi-objective optimization algorithms (e.g., [39] and [40]). Each candidate solution is a vector of *n* values, where *n* is the dimension of the problem, and is assigned an age *τ*, initially set to 0 [40, 41]. An initial population *P* ^(0)^ of dimension *d* is randomly generated, and each variable is defined in the range where the search is performed. The copying phase is responsible for the production of copies of the candidate solutions. Each member of the population is copied *dup* times, thus producing a population *P*_*cop*_ of size *d* × *dup*, where each copied candidate solution takes the same age of its parent; simultaneously, the age of the parent increments by one unit. Once the *P*_*cop*_ population is created, it undergoes the mutation phase in order to find better solutions; in this phase, two operators, called *local search* and *global search*, are applied to each candidate solution. Firstly, the local search operator mutates a randomly chosen variable *x*_*i*_ of a given candidate solution using a *self-adaptive Gaussian mutation* computed as $x_{i}^{new}=x_{i}+\sigma_{i}^{new}N(0,1)$, where $\sigma_{i}^{new}=\sigma_{i}e^{\gamma N(0,1)+\gamma^{\prime }N_{i}(0,1)}$, with initial conditions $\sigma_{i}=0.4(x_{i}^{max}-x_{i}^{min})/\sqrt{n},\gamma =1/\sqrt{2n},\gamma^{\prime }=1/\sqrt{2\sqrt{n}}$, *n* being the number of decision variables.

Successively, the global search operator applies a convex perturbation to a given solution by setting $x_{i}^{new}=(1-\gamma_{i})x_{i}+\gamma_{i}x_{k}$, where *x*_*k*_ is a variable randomly chosen such that *x*_*i*_ ≠ *x*_*k*_, with *γ*_*i*_ = *N*(0,1). These local and global search operators are controlled by a specific mutation rate *α*; for the local search, we define *α* = *e* ^−*ρF*^, while for the global search operator we adopted $\alpha =\frac{1}{\beta }e^{-F}$, where *F* is the fitness function value normalized in [0, 1], while *ρ* and *β* are parameters. The fitness function is defined as $F=\sum_{i=1}^{r}f_{i}/f_{max_{i}}$ where *f* is the vector of the *r* objective functions and *f*_*max*_ is the vector of the *r* best values (of all the populations), one for each objective function. *F* is normalized dividing by *r*.

The two operators are applied sequentially; the local search operator acts on the *P*_*cop*_ producing a new population *P*_*LS*_. The global search operator mutates *P*_*LS*_ generating the *P*_*GS*_ population. After the local search operator, the population *P*_*GS*_ is evaluated; if a candidate solution achieves a better value of objective function, its age is set to 0, otherwise it is increased by one. Then, the *diversity enforcing* operator is applied to *P* ^(*t*)^ and *P*_*GS*_; it deletes candidate solutions with an age greater than *τ*_*B*_ + 1, where *τ*_*B*_ is a parameter of the algorithm. The deleted candidate solutions are saved into the archive *BC*_*arch*_. Since the archive contains at most *s*_*a*_ solutions, if there is enough space, the candidate solution is put into the first available location, otherwise it is put in a random location. Finally, the selection is performed and the new population *P* ^(*t* + 1)^ is created by picking the best individuals from the parents and the mutated candidate solutions. However, if ∣*P* ^(*t* + 1)^∣ < *d*, *d* − ∣*P* ^(*t* + 1)^∣ candidate solutions are randomly picked from the archive and added to the new population.

In many real world applications, it is common to deal with constraints, which could be imposed on input and output values [42]. In general, a constraint is a function *g*(*x*) that certificates if a solution for a given optimization problem is *feasible* or not. We consider constraints defined as *g*(*x*): *R* ^*n*^ → *R* if *g*(*x*) ≤ *θ*, where *θ* is a *feasibility* threshold. The algorithm considers the constraint values during the selection procedure. Given two individuals *p*_1_, *p*_2_, if both are feasible, then the individual with the lowest objective function value is picked; if *p*_1_ is feasible and *p*_2_ is unfeasible, *p*_1_ is chosen, otherwise if *p*_1_ and *p*_2_ are unfeasible the individual with the lowest constraints violation is selected.

In section 1, all the simulations are performed with the following algorithm parameters: *d* = 20, *dup* = 2, *τ*_*B*_ = 50, *ρ* = 1, *β* = 7 and *s*_*a*_ = 160. Additionally, the model (input of OptBioCAD) can be a FBA, FBA-GPR, ODEs or DAEs model. For each model a vector $\vec{mp}$ of parameters is defined and its elements are used as decision variables. The output of the algorithm will be *P* ^(*t*)^, where *t* = *t*_*final*_, and will contain the optimal designs, ${\vec{mp}}_{i}^{*},i=1,\ldots ,d$. Moreover, *s*_*a*_ additional solutions will be contained in the *BC*_*arch*_ archive and in *P* ^(*t*)^, where *t* = 0, 1, …, *t*_*final*_. In section 1 and 3, the results come from these two last sets. The pseudo-code of OptBioCAD is reported in Table 1. The choice of the biomass production as a goal to optimize (e.g., allowing the maximal growth of the organism), is common for FBA models [43].

**Table 1:** optBioCAD Pseudo-code.

1: **optBioCAD** (*model*, *d*, *dup*, *τ*_*B*_, *ρ*, *β*, *s*_*a*_) /* the model can be FBA, FBA-GPR, ODEs or DAEs */
2: *t* ← 0;
3: *BC*_*arch*_ ← *Create*_*Archive*(*s*_*a*_);
4: *P* ^(*t*)^ ← *Initialize*(*d*);
5: *Evaluate*(*P* ^(*t*)^, *model*);
6: *EvaluateConstraints*(*P* ^(*t*)^, *model*);
7: **while** ¬*Stop*_*Condition*(*t*) **do**
8: *P*_*cop*_ ← *Copying*(*P* ^(*t*)^, *dup*);
9: *P*_*LS*_ ← *Local*_*Search*_*Operator*(*P*_*cop*_, *ρ*);
10: *P*_*GS*_ ← *Global*_*Search*_*Operator*(*P*_*LS*_, *β*);
11: *Evaluate*(*P*_*GS*_, *model*);
12: *EvaluateConstraints*(*P*_*GS*_, *model*);
13: *Diversity*_*Enforcing*(*P* ^(*t*)^, *P*_*GS*_, *τ*_*B*_);
14: *BC*_*arch*_ ← (*BC*_*arch*_ ∪ *P* ^(*t*)^ ∪ *P*_*GS*_);
15: *P* ^(*t* + 1)^ ← *Selection*(*P* ^(*t*)^, *P*_*GS*_, *BC*_*arch*_);
16: *t* ← *t* + 1;
17: **end while**
18: **return** (*P* ^(*t*)^); /* output the best *d* candidate solutions (metabolic networks) */

### 4.3 *ϵ*-dominance analysis

The *ϵ*-dominance analysis, inspired by Laumanns et al. [44], is a technique that improves the diversity of the solutions and the convergence of the optimization algorithm. In Section 1, we highlighted that a point y* in the solution space is said to be Pareto optimal if there does not exist a point *y* such that *f*_*i*_(*y*) > *f*_*i*_(y*), ∀*i* = 1, …, *r*, where *f* is the vector of *r* objective functions to optimize in the objective space. The *ϵ*-dominance technique applies a “relaxed” condition of dominance. That is, a point y* in the solution space is said to be *ϵ*-non-dominated if there does not exist a point *y* such that *f*(*y*) dominates *f*(y*) of a value higher than *ϵ*. Formally, y* is said to be *ϵ*-non-dominated if ∄ *y* s.t. *f*_*i*_(*y*) ≥ *f*_*i*_(y*)+*ϵ*_*i*_, *ϵ*_*i*_ > 0, ∀*i* = 1, …, *r*. This “relaxed” condition captures both the “*ϵ*-non-dominated” solutions and the non-dominated ones (Pareto-optimal), since a Pareto-optimal solution is also *ϵ*-non-dominated, but the converse does not hold. This technique allows suboptimal solutions to remain in the population, therefore increasing diversity and facilitating the search for multi-modal solutions in single objective optimization problems.

In our work, we use this method to seek solutions that may have been discarded because they are dominated by a small amount *ϵ* that, for our purposes, can be considered negligible. After the optimization, we perform an *ϵ*-dominance analysis to search accurately near the edge of the Pareto-optimal region. Formally, let *f* be the array of the *r* objective functions, and suppose that all the objective functions are positive and must be maximized. Let *ϵ* > 0 be the tolerance of our relaxed condition. We seek all points (solutions) y* belonging to the set {y* : *f*_*i*_(y*) + *ϵ*_*i*_ ≥ *f*_*i*_(*y*), ∀*i* = 1, …, *r*},where *f* is the vector of the *r* objective functions, and *y* represents the non-dominated points. This set will contain both the new “*ϵ*-non-dominated” points and the old non-dominated ones. The “*ϵ*-non-dominated” solutions can be considered suboptimal solution because they are close to the Pareto-optimal region. To better understand this analysis, we report some examples in Section 2 in S1 File.

### 4.4 Identifiability analysis

A biological model is made up of many components (e.g., parameters, variables) estimated through fitting to experiments. The Identifiability Analysis (IA) seeks the functional relations underlying the components of a given system, and can be used after the multi-objective optimization. Coupled with the sensitivity analysis, it gives insight into the model under investigation.

A component is said to be *non-identifiable* if there is no unique solution for its estimation. The non-identifiability can be (i) *structural*, when there are relations among components and therefore they cannot be determined unambiguously; (ii) *practical*, when the low amount or quality of data available does not allow achieving a good estimate for the component. From the definitions, it follows that if a model is structurally non-identifiable, it is also practically non-identifiable. Conversely, the structural identifiability does not necessarily imply the practical identifiability. Using repeated fitting to data and estimations of components, the IA is aimed at finding the structural non-identifiable components of a model, providing hints for simplifying the model and thus avoiding redundancy, or indicating where new experimental measures are needed to ensure the identifiability of the model.

Specifically, let *m* be the number of decision variables {*x*_1_, …, *x*_*m*_} of the model, which are related by unknown linear or non-linear functional relations. Let *n* be the number of estimates available for each variable. These estimates are usually organized into a matrix *K* = [*v*_1_, …, *v*_*m*_] ∈ ℝ^*n* × *m*^, where each column array *v*_*i*_ ∈ ℝ^*n*^ consists of the *n* estimates for the *i*th variable.

With the aim of detecting relations among flux rates, we assign a variable *x*_*i*_ to each flux rate, *i* = 1, …, *m*. Let us denote by *α* and *β*_*j*_ the true transformations that linearize the relations among variables:

$$\begin{array}{c} \alpha \left(x_{i}\right)=\sum_{j\neq i}^{m}\beta_{j}\left(x_{j}\right)+\xi , \end{array}$$ (3)

where *ξ* is a Gaussian noise. The alternating conditional expectation (ACE) algorithm [45] estimates the optimal transformations $\hat{\alpha }(x_{i})$ and $\hat{\beta_{j}}(x_{j}),j\neq i$, such that

$$\begin{array}{c} \hat{\alpha }\left(x_{i}\right)=\sum_{j\neq i}^{m}\hat{\beta_{j}}\left(x_{j}\right), \end{array}$$ (4)

where *x*_*i*_ is the response, while all the other variables are the predictors. Starting from an initial guess of *α*(*x*_*i*_) = *x*_*i*_/‖*x*_*i*_‖ and *β*_*j*_(*x*_*j*_) = 0, *j* ≠ *i*, ACE iteratively estimates *α* and {*β*_*j*_}_*j* ≠ *i*_ based on the minimization of the square residuals of (Eq 4).

As a post processing step of the multi-objective optimization ATP-NADH of the FBA mitochondrial model [17], we use the IA to find all the relations among reaction fluxes. The mitochondrial FBA model is composed of 423 reactions, of which *p* = 73 are the “input fluxes”, i.e., reactions transporting metabolites from the external environment into the mitochondrion; 135 are the matrix reactions, i.e., all those taking place in the matrix compartment, e.g., Krebs cycle and beta-oxidation; all the remaining reactions take place in the inner membrane space.

We take into account 135 decision variables, namely the 135 fluxes of the matrix reactions in the 2000 mitochondrial conditions obtained with 2000 different values of fumarate. To perform the IA, we infer functional relations with the ACE algorithm within the method proposed by Hengl et al. [46]. In this way, we seek structural identifiability affecting decision variables. Each row of he matrix *K*, in the standard ACE approach, is obtained by estimating parameters of a system. Specifically, a row contains the estimated parameters, obtained by minimizing the square residuals between experimental data and model predictions, but with a different initial guess of the parameters in each row. In our case, this is replaced by repeating the FBA analysis for each fumarate value, thus taking into account all the configurations found by the FBA run with different fumarase flux. Therefore, the key result of our approach is the possibility to highlight stage-specific functional relations among reactions in the three monogenic diseases. Since the fitting matrix *K* consists of the 135 columns in *V* associated with matrix fluxes (see Section 3 for the definition of *V*), we reduce the problem of finding structural non-identifiability to the problem of detecting groups of interdependent fluxes in *K*. We note that the identifiability analysis depends on the constraints, since a non-identifiable constraint involving decision variables is a functional relation between them. In our approach, the constraint is detected by performing 2000 estimates of the 135 variables (matrix fluxes), with the aim of characterizing three mitochondrial diseases by looking at the functional relations among the fluxes in the model. We use the Mean Optimal Transformation Approach (MOTA) [46] and the ACE algorithm, fixing at 10 the maximum number of reactions allowed to enclose a functional relation.

In S1 File we report the tables summarizing the results of the IA applied to the three monogenic diseases. There are fluxes showing functional relations in a group of more than two elements, although in this case strong relations among variables are less likely. We remark that a variable is detected in a group depending on the contribution strength of a predictor to the response. Each variable is considered once as response variable. As a result, if a functional relation is among *k* variables, it is tested *k* times, although it is unlikely that the same functional group is really detected all the *k* times. The *r* ^2^ column indicates how much variance of the response can be explained by the predictors. A high amount of explained variance of the response indicates a significant effect of the fixation of the predictors on the standard deviations of the response. The *cv*(*x*) = *std*(*x*)/*mean*(*x*) helps to distinguish practical identifiable from non-identifiable variables [46]. In case of practical non-identifiability, the choice of the value that needs to be fixed strongly depends on the experiments, also considering reference values in the literature.

### 4.5 Sensitivity analysis

The Sensitivity Analysis (SA) is a method that evaluates the importance of the input(s) in a model. The idea is to randomly perturb the input(s) of a model in order to obtain a distribution of elementary effects (on the output) due to the perturbations of each input. An elementary effect is calculated by comparing (subtracting) the output(s) of the model when the input is perturbed, with the output(s) of the model without perturbation. Plotting the average and the standard deviation of the distribution of elementary effects gives an idea of how the perturbation of the input affects the output(s) of the model. If the mean of the distribution is large, the input has an important overall influence on the output(s) [47]. If the distribution has a large standard deviation, the input has a high influence depending on the values of the other inputs.

The SA is frequently used for the in-silico design of electronic devices, and in the last decade it has been widely used in bioengineering. In electronic design automation, the input(s) of the model can be gain and tension, or resistance and conductance. Conversely, in a biological model the input(s) of the model can be: (i) nutrients of a cell, for instance the uptake rate of glucose or oxygen; (ii) gene knockouts in the genome of a bacterium, for instance the knockout of the pyruvate dehydrogenase complex; (iii) enzyme concentrations in a metabolic pathway, for instance the concentration of RuBisCO in a plant cell. According to the modelling technique and the parameters included in the model, the SA provides a ranking of selected input(s), evaluating their importance. Our BioCAD framework includes two sensitivity methods: Morris’ [47] and Sobol’s [48] methods.

**Fig 8:**
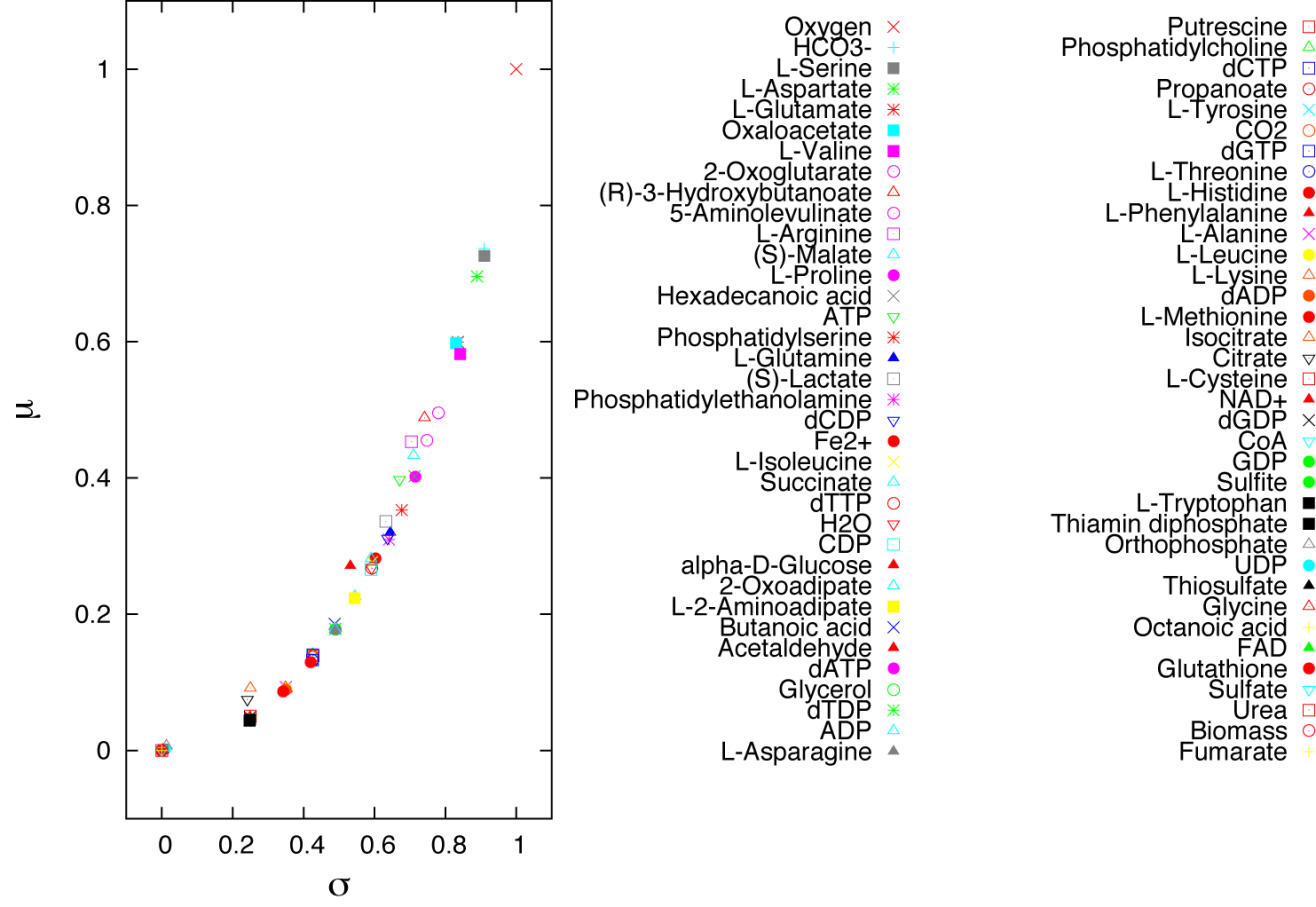
Sensitivity analysis on the mitochondrial FBA model. The plot shows the mean and the standard deviation of the elementary effects computed through the Morris’ method applied on the upper bounds of the exchange reaction fluxes. The uptake rates are ranked according to their relative influence on the ATP production. The mitochondrial ATP production is highly sensitive to changes in the uptake rate of oxygen, HCO_3_, L-serine, and L-aspartate.

In Fig 8, we show the result of the Morris method considering as input the uptake flux rates in the genome-scale metabolic network of the mitochondrion [17] discussed in Section 2. In the plot we report the mean and the standard deviation of the distribution of elementary effects for each input. The legend in the figure ranks the inputs from the most to the less important. These results were confirmed by the local robustness analysis [7], performed through perturbation of one input at a time. Finally, a pathway-oriented SA is reported in Fig S3 in S1 File for the model of *Chlamydomonas reinhardtii* [18] discussed in Section 1.

## Supporting Information

S1 File Supporting Figs. 1–32 and Tables 1–11. (PDF)

S1 Table Best solutions found after the optimization of the FBA algal mitochondria with 10 knockouts permitted. (XLSX)

S2 Table Best solutions found after the optimization of the FBA algal mitochondria with 50 knockouts permitted. (XLSX)

S1 Code BioCAD source code. (ZIP)
