# Supplementary material for: Multi-Target Analysis and Design of Mitochondrial Metabolism
Source: PLoS One. 2015 Sep 16;10(9):e0133825. doi: 10.1371/journal.pone.0133825 (PMC4574446; doi:10.1371/journal.pone.0133825)
Supplement: S1 File — (PDF) [file pone.0133825.s001.pdf]

Multi-Target Analysis and Design of Mitochondrial Metabolism

Claudio Angione[1,*,+], Jole Costanza[2,*], Giovanni Carapezza[3], Pietro Lió[1], Giuseppe Nicosia[3]
1 Computer Laboratory - University of Cambridge, Cambridge, UK
2 Center for Genomic Science of IIT@SEMM, Istituto Italiano di Tecnologia, Milan, Italy
3 Department of Mathematics and Computer Science - University of Catania, Catania, Italy
[*] **These authors contributed equally to this work**
[+] **Email: claudio.angione@cl.cam.ac.uk**

Supplementary information

1 Optimization of the FBA algal mitochondria

Maximizing ATP and NADH production. We take into account an algal model [1] that contains reactions related to the mitochondrial functions. By using OptBioCAD we maximize ATP and NADH production. The aim is to find (i) the Pareto optimal genetic strategy or (ii) the optimal environment that increases the algal bioenergy yield.

In the first case, the decision variables are the genes and, in particular, their presence or not in the metabolic network. The gene knockout strategies are represented as binary vectors. Hence, the combinatorial optimization problem consists of finding the Pareto optimal strings of bits, which represent the Pareto optimal genetic strategies. We consider two cases, in which the maximum number of knockouts allowed is equal to 10 and 50 respectively. In the second case, we search for the best values of uptake rate fluxes. We consider three cases, in which the uptake rate fluxes can reach a maximum value of 1000, 100 and 10 $mmolh^{-1}gDW^{-1}$ respectively. Moreover, we take into account a mixed-problem: finding the optimal string of bit y^* and looking for the best values of uptake rate fluxes that increase the algal bioenergy yield. We consider two cases: (a) maximum number of knockouts allowed equal to 10 and maximum value of uptake rate equal to 10 mmolh^{-1} gDW^{-1}, and (b) maximum number of knockouts allowed equal to 50 and maximum value of uptake rate equal to 1000 mmolh^{-1} gDW^{-1}.

The results of these seven experiments are shown in Figure S1. We observe how optimal environment conditions, obtained by changing the input fluxes, lead to an increased ATP and NADH production, while the genetic strategies remain near the starting point.

Maximizing ATP production. Here we carry out three single-objective optimizations considering only the maximization of ATP. In the first case, we search the optimal genetic strategy and consider the case in which the maximum number of knockouts allowed is 10. In the second case, we search for the best values of uptake rate fluxes and consider the case in which the uptake rate fluxes can reach a maximum value of 10 mmolh^{-1} gDW^{-1}. Finally, we consider the mixed-problem: finding the optimal string of bit y^* and looking for the best values of uptake rate fluxes where we consider a maximum of ten knockouts allowed and maximum value of 10 mmolh^{-1} gDW^{-1} as uptake rate. By analysing the results, we again observe that optimal environment conditions, obtained by changing the input fluxes, allow to reach larger values. Indeed, in the first and third simulation the value of ATP reaches 3.3334 mmolh^{-1} gDW^{-1}, while in the second simulation only $4.7748 \cdot 10^{-11}$ mmolh^{-1} gDW^{-1}.

Maximizing NAD and NADH production. Finally, we maximize NAD and NADH production. The decision variables are the genes, and the maximum number of knockouts allowed is ten. SI1-Figure S7 shows the results.

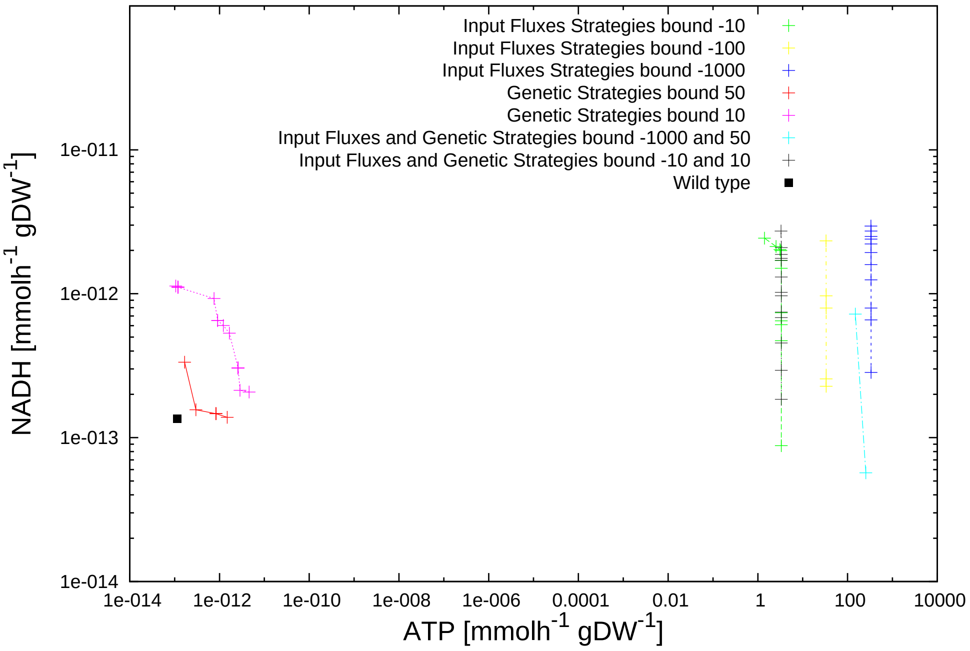

Figure S1. Comparison of the Pareto fronts obtained for the algal metabolism [1] when maximizing ATP and NADH production. The seven optimization strategies are described in Section 1, while the optBioCAD optimization algorithm is described in Section 4.2 of the main manuscript.

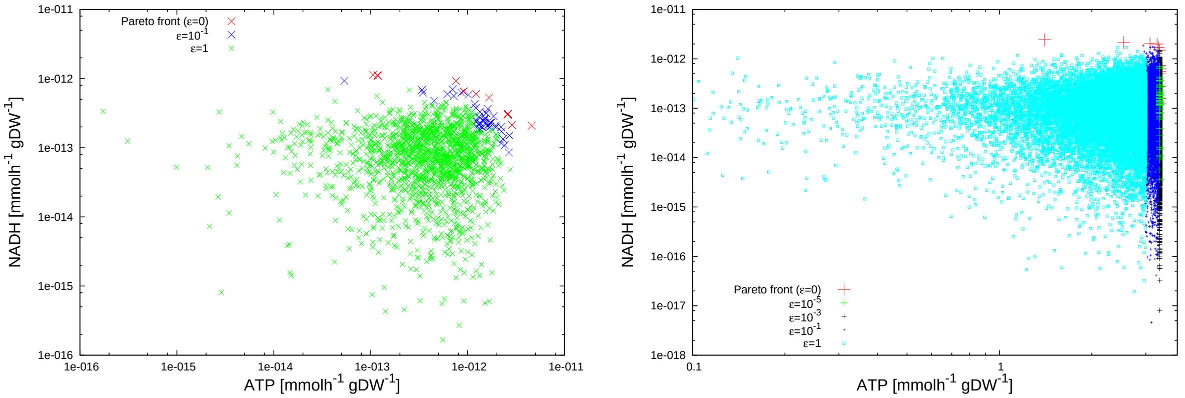

(a) (b)

Figure S2. ϵ-dominance analysis in the algal mitochondria model [1] for the maximization of ATP and NADH, with the gene sets as Boolean decision variables (a), and the fluxes as real decision variables (b). The red points are the non-dominated solutions, while the other points are suboptimal solutions found by varying epsilon. In (a), the ϵ-dominance with $\epsilon = 10^{-3}$ and $\epsilon = 10^{-5}$ revealed no additional points.

2 ϵ-dominance analysis

We consider the optimization of ATP and NADH relating to the search of genetic strategies (ten allowed knockouts), and strategies of input fluxes (with bound equal to 10 mmolh^{-1} gDW^{-1}). The corresponding Pareto front is shown in Figure S1, purple and green points respectively. They are also shown in Figure S2 (red points). In fact, if $\epsilon_i = 0, \forall i = 1, \ldots, r$, the solutions found by the ϵ-dominance analysis are non-dominated, i.e., Pareto-optimal points.

Figures S9 and S10 (red line) show the number of optimal solutions found. They are 10 and 16, respectively. Subsequently, the analysis is repeated by varying ϵ from 10^{-10} to 1. To adapt ϵ to each objective function f_i we use $\epsilon_i^* = \epsilon \cdot f_i^{max}$ where f_i^{max} is the best value obtained by f_i. In the first case (Figure S2a and S9) no suboptimal points are obtained for ϵ from 10^{-10} to 10^{-2}. Therefore, in Figure SI1-S9 the number of solutions remains equal to 10. For $\epsilon = 10^{-1}$ we have 59 points, so 49 suboptimal solutions are found. Finally, for $\epsilon = 1$, we find more than 7000 points. In Figure S2a we show these extra solutions (at different ϵ) and their position with respect to the Pareto front. In the second case (Figure S2b and SI1-S10), no suboptimal points are obtained for ϵ from 10^{-10} to 10^{-7}. The number of solutions remains equal to 16 (Figure SI1-S10). For ϵ from 10^{-6} to 10^{-1}, the number of total solutions increases from 20 to more than 90000 (only ten points are optimal, while the others are suboptimal); in Figure S2b we show their position.

3 Pathway-oriented sensitivity analysis

PoSA (Pathway-oriented Sensitivity Analysis) is able to rank the pathways of the metabolic network by perturbing genes in terms of knockout. All the genes in a pathway $b_s, s = 1, \ldots, p$, where p is the number of metabolic pathways of the network, are perturbed randomly. The output(s) of the model is compared with the output without the input perturbation. We consider as output the vector of the fluxes after performing flux balance analysis and perturbing input(s). PoSA performs combinatorial perturbation, since gene knockout are represented by means of binary variables.

For this problem, we define the "elementary effect" for the input b_s as

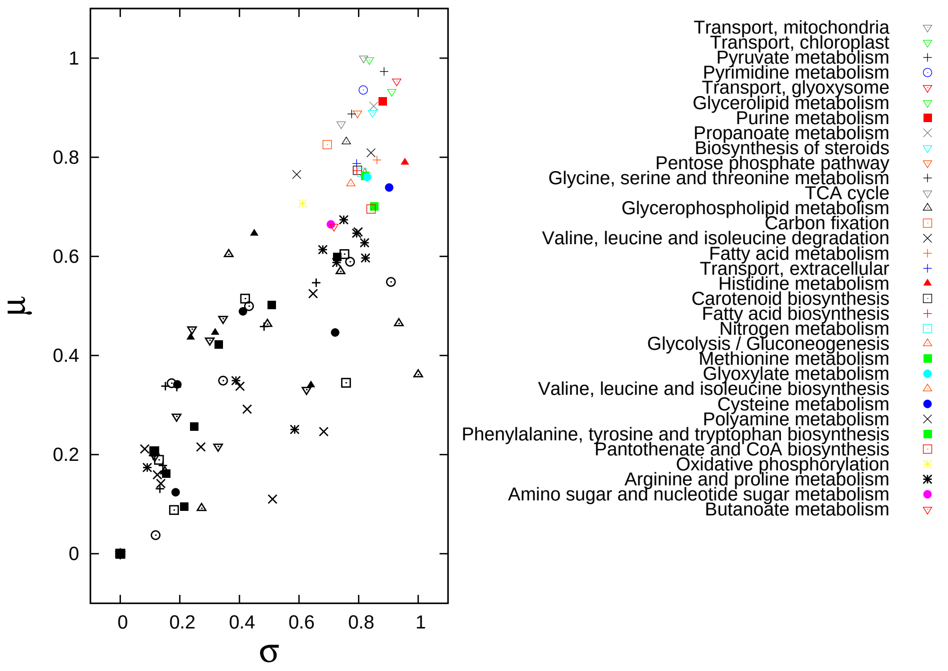

Figure S3. PoSA applied to the algal metabolism of *C. reinhardtii* [1]. In the key, only the most sensitive pathways are reported.

$$EE_s = \left[f(b_1, b_2, \ldots, b_{s-1}, \tilde{b}_s, b_{s+1}, \ldots, b_p) - f(\tilde{y}) \right] / \Delta_s, \tag{1}$$

where $\tilde{b}_s$ is the mutation on the input b_s, and consists of the *flip* of bits chosen randomly in b_s: if a bit is equal to 0 (or 1), the permutation turns it in 1 (or 0). Δ_s is a scale factor defined as $\Delta_s = \frac{1}{W_s} \sum_{i=1}^{W_s} \tilde{b}_s(i)$, $s = 1, \ldots, p$. The output $f(y)$ is the vector of output fluxes. $\tilde{y}$ is the mutation carried on the inputs defined in the real region of interest $\Omega = [0.0, 15.0]^p$, where $p = 73$ is the number of input fluxes.

The distribution of effects EE_s is obtained permuting y by randomly sampling points from Ω. The estimation of the mean $\mu*$ and standard deviation $\sigma*$ is used as indicator of which inputs should be considered important.

Figure S3 highlights that the pathway related to the "transport mitochondria" (all the reactions of transport from and to the mitochondrion) is the most sensitive. Other very sensitive pathways are the "transport chloroplast" (all the reactions of transport from and to the chloroplast), pyruvate metabolism, pyrimidine metabolism, transport glyoxysome and glycerolipid metabolism. This result underlines that the mitochondrion plays a key role in the algal metabolism.

In two additional spreadsheets, we also report the lists of genes knocked out as a result of the ATP-NADH optimization in the algal model. The two lists have been obtained with a maximum of 10 and 50 knockouts allowed in the metabolic network.

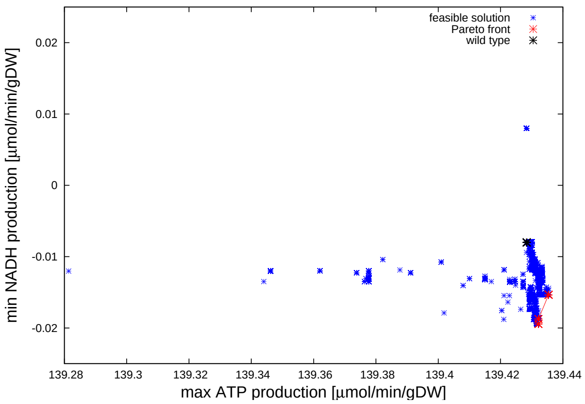

(a)

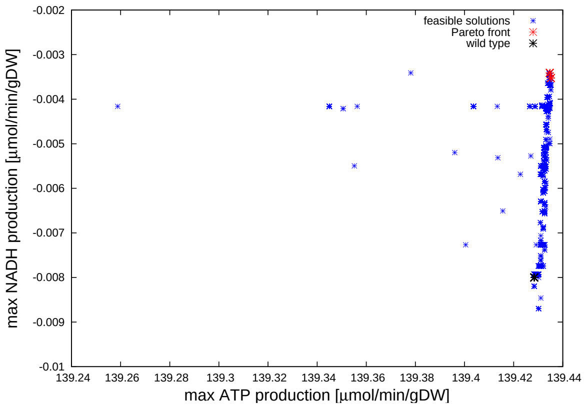

(b)

Figure S4. We seek the optimal amounts of internal fluxes in order to maximize ATP and minimize NADH (a), and to maximize ATP and NADH (b). For this optimization, we take into account the internal fluxes of the metabolic network. In this experiment we search for the optimal value of 229 metabolic fluxes. We perturb a random number (between 0 and 5) of internal fluxes by using the mutation genetic operator, and we evaluate if the configuration of the network remains feasible, i.e. satisfies all the constraints of the metabolic network. If the constraints are satisfied, we calculate the objective functions, otherwise we repeat the random mutation until a maximum number of 10 trials. Beyond 10 trials, the mutation is not performed and the current solution is maintained. Red points represent the Pareto front, blue points feasible solutions and in black the wild type condition, i.e., mitochondria before optimization.

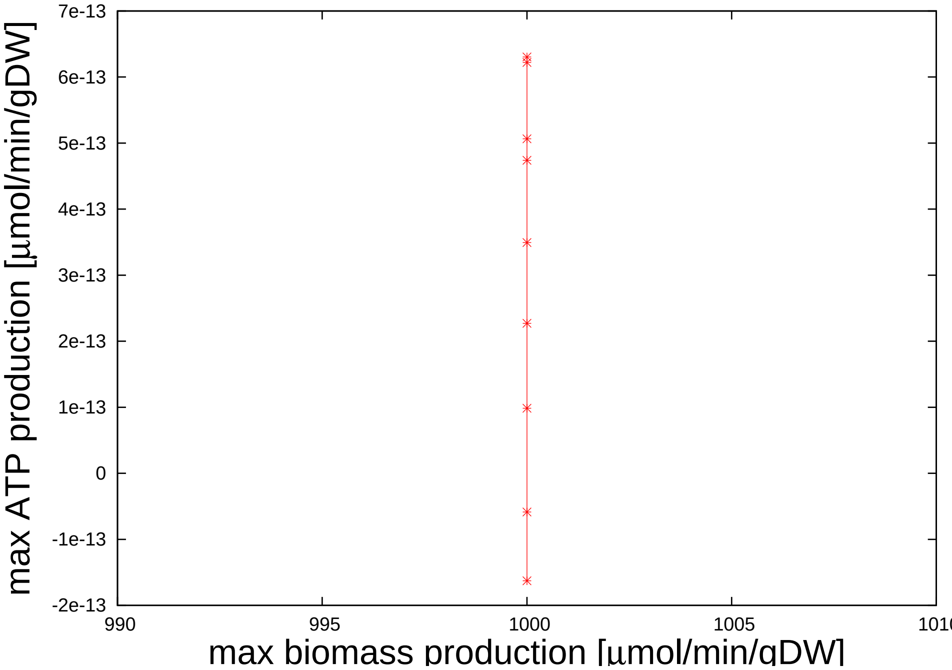

Figure S5. Pareto front obtained for the mitochondrial FBA model when optimizing ATP and biomass production, computed as the sum of the biomass in five compartments (amino acids, DNA, RNA, lipid, and heme).

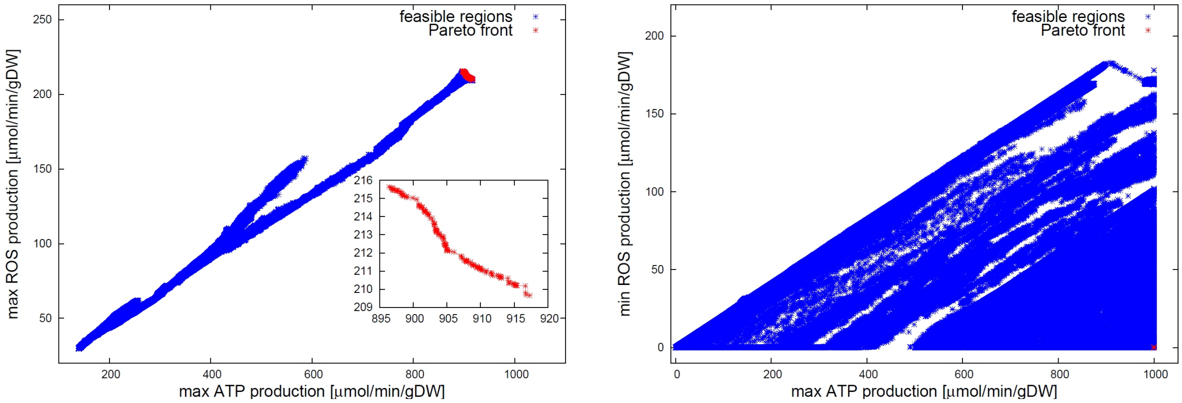

(a) (b)

Figure S6. (a) Production of ATP and reactive oxygen species (ROS) from complex I. We maximize ATP and ROS production simultaneously in the mitochondrial FBA model. (b) Maximization of ATP and concurrent minimization of reactive oxygen species (ROS) in the mitochondrial FBA model.

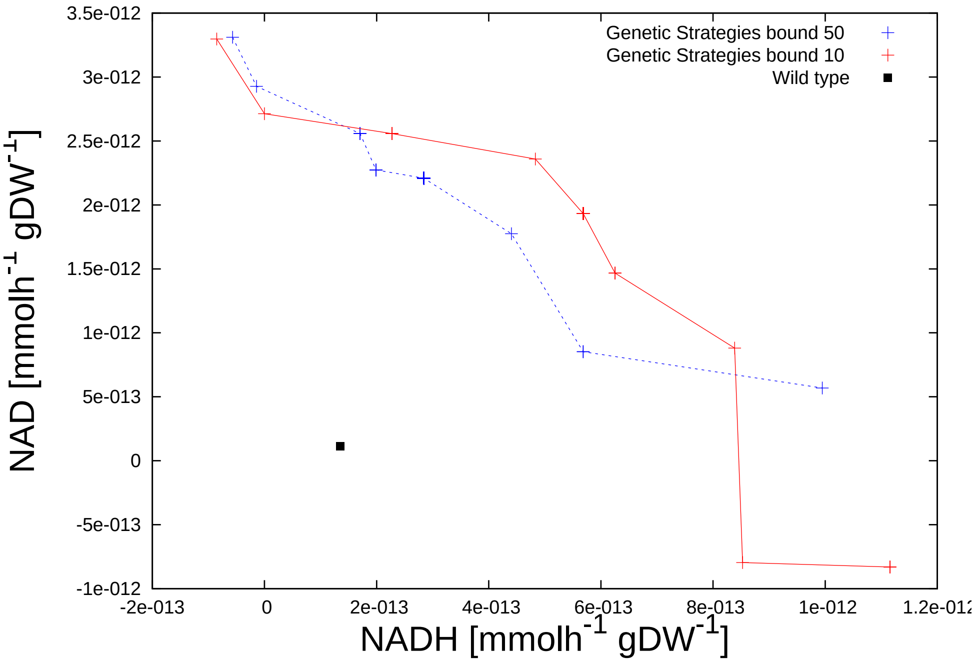

Figure S7. Pareto front obtained for the algal metabolism [1] when optimizing NAD and NADH production. We take into account the knockout status of the genes as decision variables, simulating the presence or the absence of the corresponding reactions in the metabolic network. We plot the output points corresponding to the Pareto optimal genetic strategies found with a bound of 10 and 50 knockouts allowed.

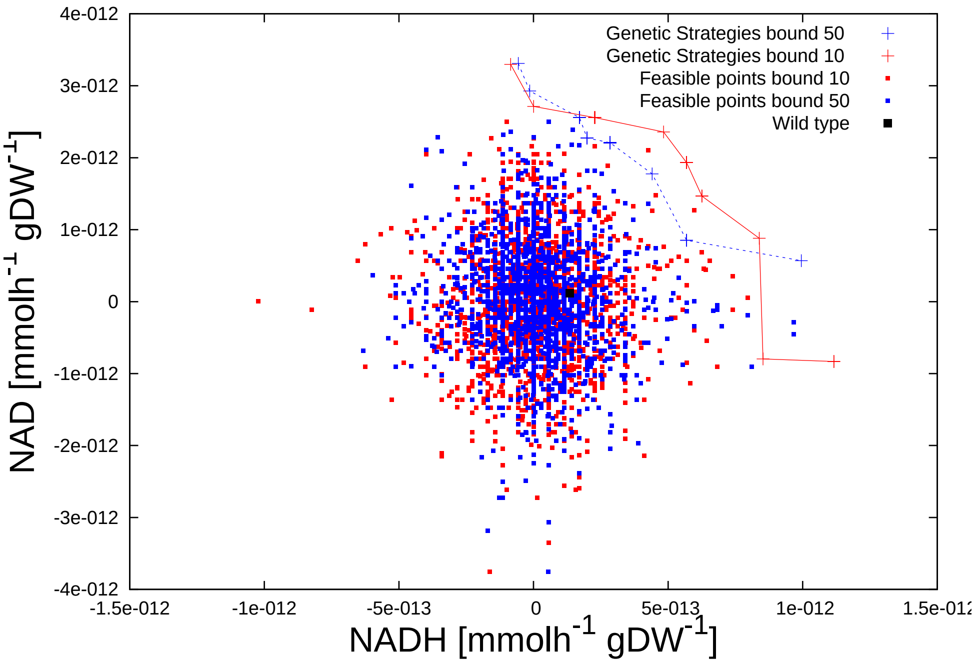

Figure S8. Feasible points associated with the Pareto front obtained in Figure S7.

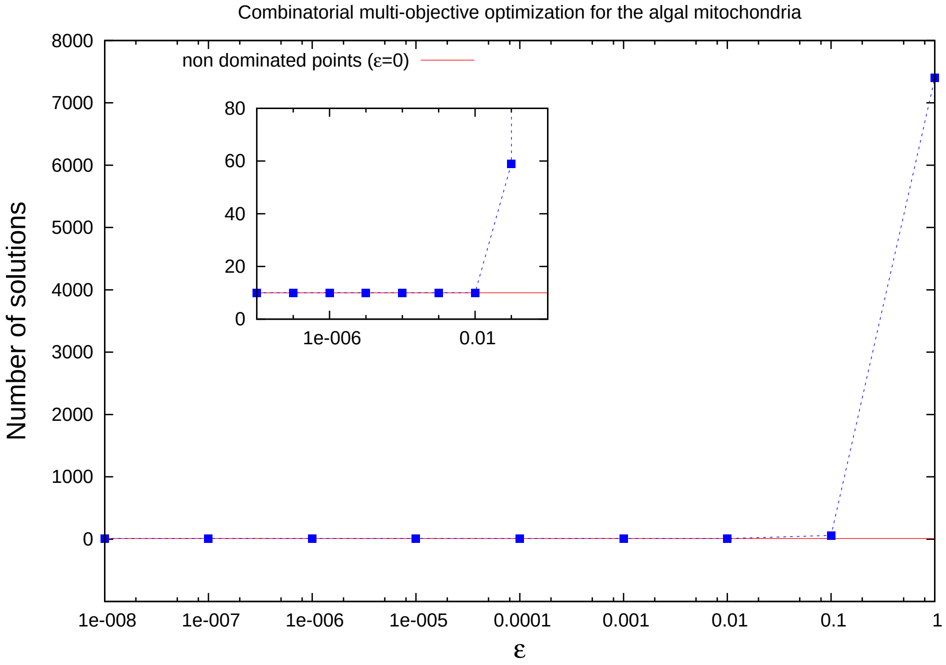

Figure S9. ϵ-dominance analysis in the algal mitochondria model [1] for the maximization of ATP and NADH, with the gene sets as Boolean decision variables. Distribution of the number of solutions as function of ϵ. The red line shows the number of Pareto-optimal solutions (non-dominated) found for $\epsilon = 0$. The blue line and points show the number of Pareto-optimal and suboptimal solutions found by varying ϵ from 10^{-10} to 1. (The inset plot shows the y range between 0 and 80, not visible in the main plot.)

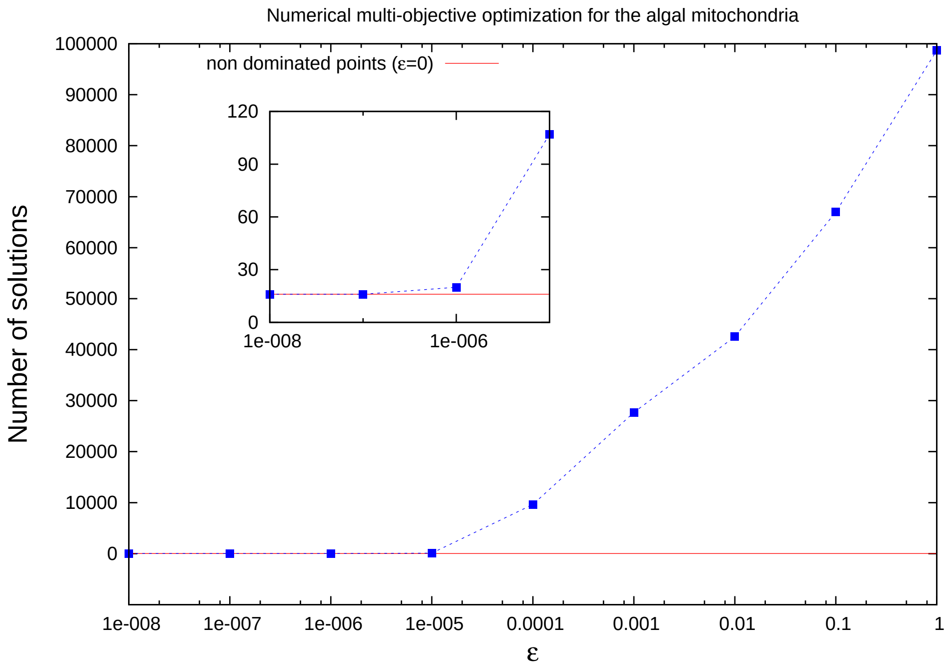

Figure S10. ϵ-dominance analysis in algal mitochondria model [1] for the maximization of ATP and NADH, with the fluxes as real decision variables. Distribution of the number of solutions as function of ϵ. The red line shows the number of Pareto-optimal solutions (non-dominated) found for $\epsilon = 0$. The blue line and points show the number of Pareto-optimal and suboptimal solutions found by varying ϵ from 10^{-10} to 1. (The inset plot shows the y range between 0 and 120, not visible in the main plot.)

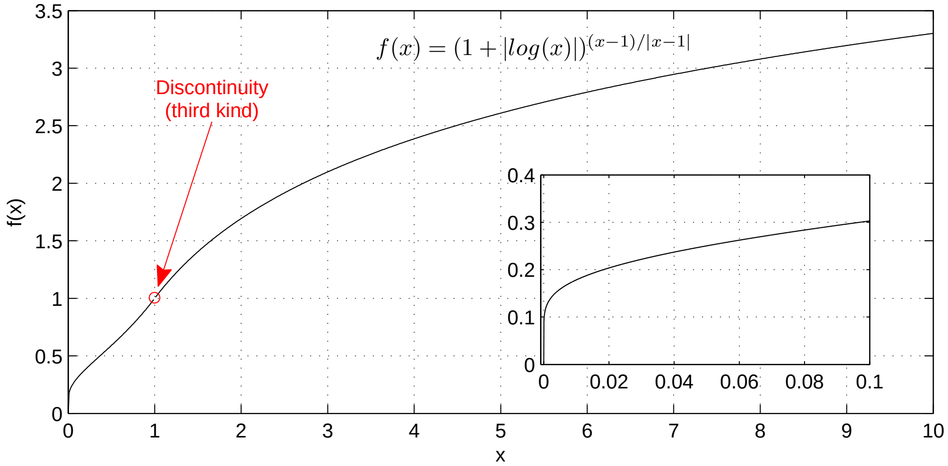

Figure S11. The function $f(x)$ used as a multiplicative factor for the gene set expression values influencing a flux. The upper and lower bound of the flux in the FBA model in a specific condition are equal to the wild-type bounds multiplied by $f(x)$. In $x = 1$ the function shown has a discontinuity of the third kind, removable by imposing $f(1) = 1$. In the inset we show the behavior of $f(x)$ in the neighborhood of 0.

Variable	Flux	Flux groups	r^2	cv
x_1	$R00004MM$	x_1, x_8, x_{12}, x_{27}*	1.000	2.056
x_2	$R00014MM$	$x_2, x_{16}, x_{45}, x_{58}, x_{72}, x_{82}, x_{98}$	n.a.	0.047
x_3	$R00081MM$	x_3, x_{64}**	1.000	0.423
x_4	$R00086MM$	$x_4, x_{13}, x_{57}, x_{65}, x_{73}$*	1.000	0.404
x_5	$R00127MM$	$x_5, x_{19}, x_{87}, x_{117}, x_{118}$*	1.000	1.233
x_6	$R00157MM$	$x_6, x_{14}, x_{85}, x_{117}, x_{118}$	n.a.	1.233
x_7	$R00205MM$	$x_7, x_{21}, x_{91}, x_{109}, x_{128}$	n.a.	0.869
x_8	$R00238MM$	$x_8, x_{21}, x_{37}, x_{57}, x_{73}$*	1.000	0.884
x_9	$R00243MM$	$x_9, x_{40}, x_{75}, x_{94}, x_{131}$*	1.000	5.136
x_{10}	$R00245MM$	$x_{10}, x_{16}, x_{57}, x_{61}, x_{78}$*	1.000	0.489
x_{11}	$R00256MM$	$x_{11}, x_{68}, x_{86}, x_{90}, x_{98}, x_{109}$	n.a.	1.271
x_{12}	$R00258MM$	$x_2, x_{12}, x_{13}, x_{43}, x_{78}, x_{98}$	n.a.	0.190
x_{13}	$R00275MM$	$x_4, x_8, x_{13}, x_{52}, x_{65}$*	1.000	0.383
x_{14}	$R00330MM$	$x_8, x_{14}, x_{86}, x_{88}, x_{91}$*	0.999	3.909
x_{15}	$R00342MM$	$x_{12}, x_{15}, x_{52}, x_{66}, x_{108}, x_{134}$*	0.998	0.420
x_{16}	$R00351MM$	$x_8, x_{16}, x_{21}, x_{57}, x_{73}$*	1.000	0.556
x_{17}	$R00355MM$	$x_2, x_{17}, x_{72}, x_{82}$	0.999	0.051
x_{18}	$R00371MM$	$x_{18}, x_{91}, x_{105}, x_{128}$*	1.000	0.869
x_{19}	$R00388MM$	$x_{19}, x_{77}, x_{108}, x_{109}, x_{110}, x_{128}$	n.a.	0.304
x_{20}	$R00430MM$	x_{14}, x_{20}, x_{134}*	0.999	3.780
x_{21}	$R00432MM$	$x_8, x_{21}, x_{37}, x_{73}, x_{77}, x_{105}$	n.a.	0.558
x_{22}	$R00512MM$	x_{22}, x_{129}**	0.999	2.648
x_{23}	$R00551MM$	$x_{23}, x_{88}, x_{107}, x_{111}$	1.000	0.000
x_{24}	$R00572MM$	$x_4, x_{13}, x_{24}, x_{65}, x_{73}$*	0.999	1.996
x_{25}	$R00667MM$	$x_{19}, x_{25}, x_{98}, x_{108}, x_{112}$	1.000	0.000
x_{26}	$R00705MM$	x_{26}, x_{94}*	1.000	41.821
x_{27}	$R00709MM$	$x_{12}, x_{15}, x_{27}, x_{45}, x_{57}, x_{108}$*	0.998	0.556
x_{28}	$R00713MM$	x_4, x_{12}, x_{28}*	0.981	2.246
x_{29}	$R00716MM$	$x_8, x_{29}, x_{45}, x_{90}, x_{98}$*	1.000	0.847
x_{30}	$R00740MM$	x_{30}, x_{40}*	1.000	41.821
x_{31}	$R00830MM$	$x_{31}, x_{40}, x_{75}, x_{76}, x_{94}, x_{130}, x_{131}, x_{135}$	n.a.	n.a.
x_{32}	$R00833MM$	x_{32}, x_{61}, x_{128}*	1.000	0.157
x_{33}	$R00851MM$	$x_{33}, x_{66}, x_{91}, x_{109}$*	1.000	2.648
x_{34}	$R00927MM$	x_{34}, x_{81}, x_{93}*	1.000	1.152
x_{35}	$R00941MM$	x_{35}, x_{51}**	1.000	0.867
x_{36}	$R00945MM$	$x_{19}, x_{29}, x_{36}, x_{88}, x_{105}, x_{111}$	n.a.	0.867
x_{37}	$R01082MM$	$x_{16}, x_{21}, x_{37}, x_{57}, x_{73}$*	1.000	0.578
x_{38}	$R01175MM$	$x_{38}, x_{68}, x_{98}, x_{104}, x_{108}, x_{110}$	n.a.	0.728
x_{39}	$R01177MM$	$x_{39}, x_{88}, x_{90}, x_{114}, x_{115}$*	1.000	0.841
x_{40}	$R01214MM$	$x_{31}, x_{40}, x_{75}, x_{76}, x_{94}, x_{130}, x_{131}, x_{135}$	n.a.	0.000
x_{41}	$R01218MM$	$x_8, x_{41}, x_{58}, x_{73}, x_{78}$*	1.000	1.196

Table A. Part I. Identifiability analysis applied to the FBA model of the mitochondrion with various fumarate conditions. The 135 matrix fluxes are grouped according to functional relations. r^2 indicates the amount of variance of the response explained by the predictors. A large $cv(x) = std(x)/mean(x)$ indicates that the data are scattered (practical non-identifiability). "n.a." stands for "not available", indicating that the response is not significantly estimated by the predictors. An asterisk is added when $r^2 > 0.9$ and $cv > 0.1$, while another asterisk is added if the same functional group with $r^2 > 0.9$ and $cv > 0.1$ has been detected even if the role of response and predictors is switched, thus highlighting a strong interdependence between the variables involved.

Variable	Flux	Flux groups	r^2	cv
x_{42}	$R01253MM$	$x_2, x_{12}, x_{13}, x_{42}, x_{58}, x_{65}, x_{82}, x_{134}$	0.999	0.000
x_{43}	$R01279MM$	$x_{43}, x_{90}, x_{104}, x_{106}, x_{108}*$	1.000	0.726
x_{44}	$R01280MM$	$x_{44}, x_{53}**$	1.000	1.582
x_{45}	$R01325MM$	$x_4, x_{27}, x_{45}, x_{57}, x_{134}*$	1.000	0.556
x_{46}	$R01360MM$	$x_8, x_{19}, x_{46}, x_{115}, x_{128}*$	1.000	1.112
x_{47}	$R01361MM$	$x_{47}, x_{62}**$	1.000	1.112
x_{48}	$R01624MM$	$x_{48}, x_{97}**$	1.000	1.582
x_{49}	$R01626MM$	$x_{49}, x_{53}*$	1.000	1.582
x_{50}	$R01648MM$	$x_{16}, x_{21}, x_{50}, x_{52}*$	0.987	2.956
x_{51}	$R01655MM$	$x_{35}, x_{51}**$	1.000	0.867
x_{52}	$R01700MM$	$x_{11}, x_{27}, x_{45}, x_{52}, x_{66}, x_{73}$	n.a.	0.558
x_{53}	$R01706MM$	$x_{44}, x_{53}**$	1.000	1.582
x_{54}	$R01799MM$	$x_{24}, x_{54}*$	0.999	2.648
x_{55}	$R01801MM$	$x_{43}, x_{55}, x_{117}, x_{118}*$	1.000	61.209
x_{56}	$R01859MM$	$x_{56}, x_{67}*$	1.000	1.152
x_{57}	$R01900MM$	$x_8, x_{16}, x_{27}, x_{45}, x_{57}*$	1.000	0.556
x_{58}	$R01923MM$	$x_{39}, x_{58}, x_{109}, x_{111}*$	1.000	0.726
x_{59}	$R01939MM$	$x_{39}, x_{45}, x_{59}, x_{79}, x_{108}, x_{112}, x_{128}$	n.a.	0.847
x_{60}	$R01940MM$	$x_{60}, x_{70}*$	1.000	0.798
x_{61}	$R01975MM$	$x_8, x_{61}, x_{66}, x_{78}, x_{104}*$	1.000	0.803
x_{62}	$R01978MM$	$x_{47}, x_{62}**$	1.000	1.112
x_{63}	$R02030MM$	$x_{15}, x_{63}, x_{105}, x_{114}, x_{116}$	n.a.	2.648
x_{64}	$R02161MM$	$x_3, x_{64}**$	1.000	0.423
x_{65}	$R02163MM$	$x_4, x_{13}, x_{21}, x_{65}, x_{66}*$	1.000	0.383
x_{66}	$R02164MM$	$x_8, x_{27}, x_{45}, x_{52}, x_{66}*$	1.000	0.558
x_{67}	$R02199MM$	$x_{67}, x_{133}**$	1.000	1.152
x_{68}	$R02241MM$	$x_{24}, x_{68}*$	0.999	2.648
x_{69}	$R02313MM$	$x_{69}, x_{104}, x_{107}, x_{112}, x_{128}*$	1.000	0.847
x_{70}	$R02487MM$	$x_{19}, x_{70}, x_{90}, x_{113}*$	1.000	0.798
x_{71}	$R02529MM$	$x_{71}, x_{88}, x_{114}, x_{128}*$	0.999	0.869
x_{72}	$R02569MM$	$x_2, x_{12}, x_{15}, x_{37}, x_{58}, x_{65}, x_{72}, x_{82}, x_{98}$	n.a.	0.047
x_{73}	$R02570MM$	$x_8, x_{21}, x_{37}, x_{73}, x_{77}, x_{88}$	n.a.	0.558
x_{74}	$R02571MM$	$x_8, x_{74}, x_{78}, x_{109}*$	1.000	0.798
x_{75}	$R02661MM$	$x_{31}, x_{40}, x_{75}, x_{76}, x_{94}, x_{130}, x_{131}, x_{135}$	n.a.	0.000
x_{76}	$R02662MM$	$x_{31}, x_{40}, x_{75}, x_{76}, x_{94}, x_{130}, x_{131}, x_{135}$	n.a.	0.000
x_{77}	$R02765MM$	$x_{43}, x_{77}, x_{91}, x_{111}, x_{128}*$	0.999	1.152
x_{78}	$R03026MM$	$x_{21}, x_{37}, x_{52}, x_{61}, x_{62}, x_{78}$	n.a.	0.803
x_{79}	$R03102MM$	$x_{59}, x_{79}, x_{104}, x_{105}, x_{108}, x_{128}$	n.a.	0.847
x_{80}	$R03172MM$	$x_{19}, x_{39}, x_{80}, x_{98}, x_{112}*$	1.000	1.152
x_{81}	$R03174MM$	$x_{34}, x_{81}*$	1.000	1.152
x_{82}	$R03270MM$	$x_2, x_{16}, x_{45}, x_{58}, x_{72}, x_{82}, x_{85}$	n.a.	0.047
x_{83}	$R03314MM$	$x_{83}, x_{86}, x_{88}, x_{109}$	1.000	0.000
x_{84}	$R03381MM$	x_{84}, x_{135}	1.000	0.000
x_{85}	$R03777MM$	$x_{85}, x_{104}, x_{112}, x_{114}, x_{115}*$	1.000	0.726
x_{86}	$R03778MM$	$x_{86}, x_{90}, x_{107}, x_{114}, x_{116}*$	1.000	0.841
x_{87}	$R03857MM$	$x_{87}, x_{104}, x_{106}, x_{110}, x_{116}*$	1.000	0.726
x_{88}	$R03858MM$	$x_{88}, x_{106}, x_{107}, x_{109}, x_{113}*$	1.000	0.841

Table A. Part II. Identifiability analysis applied to the FBA model of the mitochondrion with various fumarate conditions. The 135 matrix fluxes are grouped according to functional relations.

Variable	Flux	Flux groups	r^2	cv
x_{89}	$R03990MM$	$x_{89}, x_{110}, x_{112}, x_{114}, x_{115}$*	1.000	0.726
x_{90}	$R03991MM$	$x_{90}, x_{107}, x_{109}, x_{111}, x_{113}$*	1.000	0.841
x_{91}	$R04170MM$	$x_{91}, x_{105}, x_{109}, x_{111}, x_{113}$*	1.000	0.841
x_{92}	$R04203MM$	x_{92}, x_{132}**	1.000	1.152
x_{93}	$R04204MM$	x_{34}, x_{93}*	1.000	1.152
x_{94}	$R04224MM$	$x_{31}, x_{40}, x_{75}, x_{76}, x_{94}, x_{130}, x_{131}, x_{135}$	n.a.	0.000
x_{95}	$R04355MM$	x_{49}, x_{95}*	1.000	1.582
x_{96}	$R04428MM$	x_{96}, x_{99}**	1.000	1.582
x_{97}	$R04430MM$	x_{48}, x_{97}**	1.000	1.582
x_{98}	$R04433MM$	$x_{98}, x_{108}, x_{112}, x_{113}$*	1.000	0.720
x_{99}	$R04533MM$	x_{96}, x_{99}**	1.000	1.582
x_{100}	$R04536MM$	x_{49}, x_{100}*	0.999	1.582
x_{101}	$R04537MM$	x_{101}, x_{123}**	1.000	1.582
x_{102}	$R04543MM$	x_{102}, x_{103}*	1.000	1.582
x_{103}	$R04544MM$	x_{103}, x_{125}**	1.000	1.582
x_{104}	$R04737MM$	$x_{104}, x_{110}, x_{112}, x_{113}, x_{115}$*	1.000	0.841
x_{105}	$R04738MM$	$x_{91}, x_{105}, x_{108}, x_{111}, x_{116}$*	1.000	0.841
x_{106}	$R04739MM$	$x_{104}, x_{106}, x_{108}, x_{112}, x_{115}$*	1.000	0.841
x_{107}	$R04740MM$	$x_{39}, x_{88}, x_{105}, x_{107}, x_{114}$*	1.000	0.841
x_{108}	$R04741MM$	$x_{104}, x_{108}, x_{110}, x_{112}, x_{115}$*	1.000	0.841
x_{109}	$R04742MM$	$x_{88}, x_{91}, x_{107}, x_{109}, x_{114}$*	1.000	0.841
x_{110}	$R04743MM$	$x_{90}, x_{106}, x_{110}, x_{112}, x_{115}$*	1.000	0.841
x_{111}	$R04744MM$	$x_{86}, x_{88}, x_{109}, x_{111}, x_{116}$*	1.000	0.841
x_{112}	$R04745MM$	$x_{91}, x_{108}, x_{110}, x_{112}, x_{115}$*	1.000	0.841
x_{113}	$R04746MM$	$x_{39}, x_{88}, x_{111}, x_{113}, x_{115}$*	1.000	0.841
x_{114}	$R04747MM$	$x_{88}, x_{91}, x_{107}, x_{111}, x_{114}$*	1.000	0.841
x_{115}	$R04748MM$	$x_{90}, x_{106}, x_{108}, x_{112}, x_{115}$*	1.000	0.841
x_{116}	$R04749MM$	$x_{107}, x_{109}, x_{114}, x_{115}, x_{116}$*	1.000	0.841
x_{117}	$R04751MM$	$x_{90}, x_{110}, x_{112}, x_{115}, x_{117}$*	1.000	0.726
x_{118}	$R04754MM$	$x_{91}, x_{106}, x_{108}, x_{110}, x_{118}$*	1.000	0.726
x_{119}	$R04952MM$	x_{95}, x_{119}*	1.000	1.582
x_{120}	$R04953MM$	x_{100}, x_{120}*	1.000	1.582
x_{121}	$R04954MM$	x_{121}, x_{122}**	1.000	1.582
x_{122}	$R04956MM$	x_{121}, x_{122}**	1.000	1.582
x_{123}	$R04959MM$	x_{101}, x_{123}**	1.000	1.582
x_{124}	$R04968MM$	x_{102}, x_{124}*	1.000	1.582
x_{125}	$R04970MM$	x_{103}, x_{125}**	1.000	1.582
x_{126}	$R05064MM$	x_{40}, x_{126}	1.000	0.000
x_{127}	$R05066MM$	x_{76}, x_{127}	1.000	0.000
x_{128}	$R07162MM$	$x_{19}, x_{86}, x_{112}, x_{114}, x_{128}$*	1.000	0.304
x_{129}	$R07390MM$	x_{22}, x_{129}**	0.999	2.648
x_{130}	$R07599MM$	$x_{31}, x_{40}, x_{75}, x_{76}, x_{94}, x_{130}, x_{131}, x_{135}$	n.a.	0.000
x_{131}	$R07600MM$	$x_{31}, x_{40}, x_{75}, x_{76}, x_{94}, x_{130}, x_{131}, x_{135}$	n.a.	0.000
x_{132}	$R07603MM$	x_{92}, x_{132}**	1.000	1.152
x_{133}	$R07604MM$	x_{67}, x_{133}**	1.000	1.152
x_{134}	$R07618MM$	$x_{11}, x_{15}, x_{27}, x_{45}, x_{52}, x_{134}$	n.a.	0.424
x_{135}	$R08157MM$	$x_{31}, x_{40}, x_{75}, x_{76}, x_{94}, x_{130}, x_{131}, x_{135}$	n.a.	0.000

Table A. Part III. Identifiability analysis applied to the FBA model of the mitochondrion with various fumarate conditions. The 135 matrix fluxes are grouped according to functional relations.

Variable	Flux	Flux groups	r^2	cv
x_1	$R00004MM$	$x_1, x_5, x_{19}, x_{128}*$	1.000	0.539
x_2	$R00014MM$	x_2, x_{72}, x_{82}	1.000	0.029
x_3	$R00081MM$	x_3	0.999	0.027
x_4	$R00086MM$	$x_4, x_{13}, x_{37}, x_{52}, x_{65}$	1.000	0.061
x_5	$R00127MM$	x_5*	1.000	3.923
x_6	$R00157MM$	x_6*	1.000	3.922
x_7	$R00205MM$	$x_7, x_{18}, x_{71}**$	1.000	2.040
x_8	$R00238MM$	$x_8, x_{21}, x_{57}, x_{66}, x_{73}*$	1.000	0.302
x_9	$R00243MM$	$x_9, x_{31}, x_{76}, x_{131}, x_{135}*$	1.000	3.689
x_{10}	$R00245MM$	$x_{10}, x_{16}, x_{110}, x_{117}, x_{118}*$	1.000	0.387
x_{11}	$R00256MM$	$x_7, x_{11}, x_{18}, x_{71}*$	1.000	2.581
x_{12}	$R00258MM$	$x_{12}, x_{18}*$	0.999	0.261
x_{13}	$R00275MM$	$x_4, x_{13}, x_{57}, x_{65}**$	1.000	0.110
x_{14}	$R00330MM$	$x_{14}, x_{16}, x_{21}, x_{53}, x_{66}, x_{73}*$	1.000	2.982
x_{15}	$R00342MM$	$x_{15}, x_{45}, x_{52}, x_{66}, x_{134}*$	1.000	0.166
x_{16}	$R00351MM$	$x_8, x_{16}, x_{57}, x_{105}, x_{109}*$	1.000	0.204
x_{17}	$R00355MM$	$x_{17}, x_{60}, x_{70}, x_{74}$	0.997	0.030
x_{18}	$R00371MM$	$x_7, x_{18}*$	1.000	2.040
x_{19}	$R00388MM$	$x_{19}, x_{89}, x_{106}, x_{114}, x_{128}*$	1.000	0.373
x_{20}	$R00430MM$	$x_{14}, x_{16}, x_{20}, x_{134}*$	1.000	3.143
x_{21}	$R00432MM$	$x_8, x_{21}, x_{37}, x_{57}, x_{73}*$	1.000	0.205
x_{22}	$R00512MM$	$x_{22}*$	0.999	4.183
x_{23}	$R00551MM$	$x_{23}, x_{31}, x_{76}, x_{130}, x_{135}$	1.000	0.000
x_{24}	$R00572MM$	$x_{15}, x_{21}, x_{24}, x_{57}, x_{73}$	n.a.	1.862
x_{25}	$R00667MM$	$x_{25}, x_{31}, x_{40}, x_{94}, x_{131}$	1.000	0.000
x_{26}	$R00705MM$	$x_{26}, x_{31}*$	1.000	30.545
x_{27}	$R00709MM$	$x_{15}, x_{16}, x_{27}, x_{45}, x_{134}*$	1.000	0.204
x_{28}	$R00713MM$	$x_{28}, x_{50}**$	1.000	4.004
x_{29}	$R00716MM$	$x_{29}*$	0.998	1.903
x_{30}	$R00740MM$	$x_{30}, x_{131}*$	1.000	30.545
x_{31}	$R00830MM$	$x_{31}, x_{40}, x_{75}, x_{76}, x_{94}, x_{130}$	1.000	n.a.
x_{32}	$R00833MM$	$x_{15}, x_{21}, x_{32}, x_{37}, x_{73}, x_{92}*$	1.000	0.112
x_{33}	$R00851MM$	$x_{16}, x_{33}, x_{57}*$	1.000	4.642
x_{34}	$R00927MM$	$x_{34}, x_{75}, x_{76}, x_{130}*$	0.999	0.491
x_{35}	$R00941MM$	$x_{35}, x_{40}, x_{75}, x_{131}, x_{135}*$	0.999	2.024
x_{36}	$R00945MM$	$x_{36}, x_{75}, x_{76}, x_{94}, x_{130}*$	0.999	2.024
x_{37}	$R01082MM$	$x_{21}, x_{37}, x_{57}, x_{73}, x_{134}*$	1.000	0.210
x_{38}	$R01175MM$	$x_{38}, x_{104}, x_{108}, x_{110}*$	1.000	0.281
x_{39}	$R01177MM$	$x_{39}, x_{58}, x_{107}, x_{111}, x_{113}*$	1.000	0.281
x_{40}	$R01214MM$	$x_{31}, x_{40}, x_{75}, x_{76}, x_{94}, x_{130}$	1.000	0.000
x_{41}	$R01218MM$	$x_{41}, x_{51}**$	1.000	2.024

Table B. Part I. Healthy stage in the fumarase deficiency. Six functional groups were detected two times with $r^2 > 0.9$ and $cv > 0.1$, even with the roles of response and predictors switched.

Variable	Flux	Flux groups	r^2	cv
x_{42}	$R01253MM$	$x_{31}, x_{42}, x_{75}, x_{131}, x_{135}$	1.000	0.000
x_{43}	$R01279MM$	$x_{43}, x_{85}, x_{86}, x_{115}, x_{118}$*	1.000	0.281
x_{44}	$R01280MM$	x_{44}, x_{49}*	1.000	0.932
x_{45}	$R01325MM$	$x_{8}, x_{16}, x_{27}, x_{45}, x_{57}$*	1.000	0.204
x_{46}	$R01360MM$	x_{46}*	0.996	0.439
x_{47}	$R01361MM$	x_{47}*	1.000	0.439
x_{48}	$R01624MM$	x_{48}, x_{97}, x_{121}*	1.000	0.932
x_{49}	$R01626MM$	x_{49}, x_{53}, x_{100}*	1.000	0.932
x_{50}	$R01648MM$	x_{28}, x_{50}**	1.000	3.995
x_{51}	$R01655MM$	x_{41}, x_{51}**	1.000	2.024
x_{52}	$R01700MM$	$x_{15}, x_{27}, x_{52}, x_{73}, x_{134}$*	1.000	0.205
x_{53}	$R01706MM$	x_{53}, x_{125}*	0.999	0.932
x_{54}	$R01799MM$	$x_{8}, x_{21}, x_{54}, x_{73}$*	1.000	4.383
x_{55}	$R01801MM$	x_{55}	n.a.	46.807
x_{56}	$R01859MM$	x_{56}, x_{94}*	1.000	0.491
x_{57}	$R01900MM$	$x_{13}, x_{16}, x_{37}, x_{57}, x_{65}$*	1.000	0.204
x_{58}	$R01923MM$	$x_{58}, x_{86}, x_{90}, x_{91}, x_{105}$*	1.000	0.281
x_{59}	$R01939MM$	x_{59}*	0.998	1.903
x_{60}	$R01940MM$	x_{60}, x_{70}, x_{74}**	1.000	0.421
x_{61}	$R01975MM$	$x_{16}, x_{27}, x_{57}, x_{61}, x_{78}$*	1.000	0.273
x_{62}	$R01978MM$	x_{62}*	0.999	0.440
x_{63}	$R02030MM$	x_{63}*	0.999	4.179
x_{64}	$R02161MM$	x_{64}	n.a.	0.027
x_{65}	$R02163MM$	$x_{4}, x_{13}, x_{57}, x_{65}$**	1.000	0.110
x_{66}	$R02164MM$	$x_{15}, x_{27}, x_{52}, x_{66}, x_{134}$*	1.000	0.206
x_{67}	$R02199MM$	x_{67}, x_{130}*	1.000	0.491
x_{68}	$R02241MM$	x_{68}*	0.998	4.237
x_{69}	$R02313MM$	x_{69}*	0.999	1.903
x_{70}	$R02487MM$	x_{60}, x_{70}, x_{74}**	1.000	0.421
x_{71}	$R02529MM$	x_{7}, x_{18}, x_{71}**	1.000	2.040
x_{72}	$R02569MM$	x_{2}, x_{72}, x_{82}	1.000	0.029
x_{73}	$R02570MM$	$x_{8}, x_{21}, x_{37}, x_{52}, x_{73}$*	1.000	0.205
x_{74}	$R02571MM$	x_{60}, x_{70}, x_{74}**	1.000	0.421
x_{75}	$R02661MM$	$x_{31}, x_{40}, x_{75}, x_{76}, x_{94}, x_{130}$	1.000	0.000
x_{76}	$R02662MM$	$x_{31}, x_{40}, x_{75}, x_{76}, x_{94}, x_{130}$	1.000	0.000
x_{77}	$R02765MM$	$x_{40}, x_{76}, x_{77}, x_{94}, x_{135}$*	0.998	0.491
x_{78}	$R03026MM$	$x_{8}, x_{61}, x_{78}, x_{91}, x_{113}$*	1.000	0.273
x_{79}	$R03102MM$	$x_{31}, x_{75}, x_{79}, x_{94}$*	0.999	1.903
x_{80}	$R03172MM$	x_{80}, x_{133}*	1.000	0.491
x_{81}	$R03174MM$	$x_{31}, x_{81}, x_{130}, x_{135}$*	0.999	0.491
x_{82}	$R03270MM$	x_{2}, x_{72}, x_{82}	1.000	0.029
x_{83}	$R03314MM$	$x_{31}, x_{75}, x_{76}, x_{83}, x_{94}$	n.a.	0.000
x_{84}	$R03381MM$	x_{84}, x_{130}	1.000	0.000
x_{85}	$R03777MM$	$x_{39}, x_{85}, x_{115}, x_{117}, x_{118}$*	1.000	0.281
x_{86}	$R03778MM$	$x_{86}, x_{90}, x_{108}, x_{114}, x_{116}$*	1.000	0.281
x_{87}	$R03857MM$	$x_{43}, x_{87}, x_{108}, x_{111}, x_{117}$*	1.000	0.281
x_{88}	$R03858MM$	$x_{88}, x_{91}, x_{111}, x_{113}, x_{114}$*	1.000	0.281

Table B. Part II. Healthy stage in the fumarase deficiency.

Variable	Flux	Flux groups	r^2	cv
x_{89}	$R03990MM$	$x_{85}, x_{89}, x_{106}, x_{112}, x_{115}*$	1.000	0.281
x_{90}	$R03991MM$	$x_{58}, x_{88}, x_{90}, x_{105}, x_{113}*$	1.000	0.281
x_{91}	$R04170MM$	$x_{91}, x_{108}, x_{111}, x_{113}, x_{116}*$	1.000	0.281
x_{92}	$R04203MM$	$x_{92}*$	0.999	0.491
x_{93}	$R04204MM$	$x_{93}, x_{94}*$	1.000	0.491
x_{94}	$R04224MM$	$x_{31}, x_{40}, x_{75}, x_{76}, x_{94}, x_{130}$	1.000	0.000
x_{95}	$R04355MM$	$x_{49}, x_{95}*$	1.000	0.932
x_{96}	$R04428MM$	$x_{96}, x_{100}, x_{121}*$	1.000	0.932
x_{97}	$R04430MM$	$x_{97}, x_{122}*$	1.000	0.932
x_{98}	$R04433MM$	$x_{38}, x_{39}, x_{58}, x_{98}, x_{116}*$	1.000	0.277
x_{99}	$R04533MM$	$x_{99}, x_{120}**$	1.000	0.932
x_{100}	$R04536MM$	$x_{49}, x_{100}, x_{120}*$	1.000	0.932
x_{101}	$R04537MM$	$x_{101}, x_{121}, x_{122}*$	1.000	0.932
x_{102}	$R04543MM$	$x_{53}, x_{102}*$	0.999	0.932
x_{103}	$R04544MM$	$x_{49}, x_{103}, x_{125}*$	1.000	0.932
x_{104}	$R04737MM$	$x_{85}, x_{87}, x_{91}, x_{104}, x_{110}*$	1.000	0.281
x_{105}	$R04738MM$	$x_{86}, x_{90}, x_{105}, x_{107}, x_{109}*$	1.000	0.281
x_{106}	$R04739MM$	$x_{39}, x_{85}, x_{87}, x_{106}, x_{117}*$	1.000	0.281
x_{107}	$R04740MM$	$x_{58}, x_{86}, x_{88}, x_{107}, x_{111}*$	1.000	0.281
x_{108}	$R04741MM$	$x_{85}, x_{108}, x_{110}, x_{112}, x_{115}*$	1.000	0.281
x_{109}	$R04742MM$	$x_{105}, x_{109}, x_{111}, x_{114}, x_{116}*$	1.000	0.281
x_{110}	$R04743MM$	$x_{43}, x_{87}, x_{110}, x_{112}, x_{116}*$	1.000	0.281
x_{111}	$R04744MM$	$x_{43}, x_{91}, x_{111}, x_{113}, x_{114}*$	1.000	0.281
x_{112}	$R04745MM$	$x_{91}, x_{106}, x_{108}, x_{112}, x_{117}*$	1.000	0.281
x_{113}	$R04746MM$	$x_{39}, x_{107}, x_{109}, x_{111}, x_{113}*$	1.000	0.281
x_{114}	$R04747MM$	$x_{58}, x_{91}, x_{111}, x_{113}, x_{114}*$	1.000	0.281
x_{115}	$R04748MM$	$x_{43}, x_{85}, x_{112}, x_{115}, x_{117}*$	1.000	0.281
x_{116}	$R04749MM$	$x_{39}, x_{86}, x_{87}, x_{114}, x_{116}*$	1.000	0.281
x_{117}	$R04751MM$	$x_{85}, x_{104}, x_{106}, x_{110}, x_{117}*$	1.000	0.281
x_{118}	$R04754MM$	$x_{85}, x_{104}, x_{108}, x_{112}, x_{118}*$	1.000	0.281
x_{119}	$R04952MM$	$x_{95}, x_{119}*$	1.000	0.932
x_{120}	$R04953MM$	$x_{99}, x_{120}**$	1.000	0.932
x_{121}	$R04954MM$	$x_{101}, x_{121}*$	1.000	0.932
x_{122}	$R04956MM$	$x_{97}, x_{101}, x_{122}, x_{123}*$	1.000	0.932
x_{123}	$R04959MM$	$x_{121}, x_{122}, x_{123}*$	1.000	0.932
x_{124}	$R04968MM$	$x_{49}, x_{124}*$	1.000	0.932
x_{125}	$R04970MM$	$x_{103}, x_{125}*$	1.000	0.932
x_{126}	$R05064MM$	x_{94}, x_{126}	1.000	0.000
x_{127}	$R05066MM$	x_{127}, x_{130}	1.000	0.000
x_{128}	$R07162MM$	$x_{19}, x_{107}, x_{113}, x_{114}, x_{128}*$	1.000	0.373
x_{129}	$R07390MM$	$x_{129}*$	0.999	4.177
x_{130}	$R07599MM$	$x_{31}, x_{40}, x_{75}, x_{76}, x_{94}, x_{130}$	1.000	0.000
x_{131}	$R07600MM$	$x_{31}, x_{40}, x_{75}, x_{76}, x_{94}, x_{131}$	1.000	0.000
x_{132}	$R07603MM$	$x_{76}, x_{132}*$	1.000	0.491
x_{133}	$R07604MM$	$x_{31}, x_{40}, x_{76}, x_{133}*$	1.000	0.491
x_{134}	$R07618MM$	$x_{15}, x_{21}, x_{27}, x_{52}, x_{134}*$	1.000	0.168
x_{135}	$R08157MM$	$x_{31}, x_{40}, x_{75}, x_{76}, x_{94}, x_{135}$	1.000	0.000

Table C. Part III. Healthy stage in the fumarase deficiency.

Variable	Flux	Flux groups	r^2	cv
x_1	$R00004MM$	$x_1, x_{44}, x_{54}*$	0.995	2.135
x_2	$R00014MM$	x_2, x_{72}, x_{82}	1.000	0.000
x_3	$R00081MM$	$x_3, x_{15}, x_{16}, x_{50}, x_{64}, x_{98}*$	1.000	0.115
x_4	$R00086MM$	$x_4, x_{13}, x_{108}, x_{117}*$	1.000	0.113
x_5	$R00127MM$	x_5*	0.912	0.267
x_6	$R00157MM$	x_6	n.a.	0.267
x_7	$R00205MM$	x_7	n.a.	0.000
x_8	$R00238MM$	$x_8, x_{50}, x_{78}, x_{91}, x_{111}, x_{114}*$	1.000	0.199
x_9	$R00243MM$	$x_9, x_{23}, x_{25}, x_{26}, x_{29}, x_{30}$	1.000	n.a.
x_{10}	$R00245MM$	$x_{10}, x_{43}, x_{108}, x_{110}, x_{128}$	1.000	0.032
x_{11}	$R00256MM$	x_{11}	n.a.	0.000
x_{12}	$R00258MM$	x_{12}	n.a.	0.000
x_{13}	$R00275MM$	$x_{13}, x_{27}, x_{85}, x_{89}*$	1.000	0.104
x_{14}	$R00330MM$	$x_{14}, x_{73}, x_{91}*$	1.000	0.268
x_{15}	$R00342MM$	$x_{10}, x_{15}, x_{64}, x_{98}$	1.000	0.082
x_{16}	$R00351MM$	$x_{16}, x_{19}, x_{88}, x_{90}, x_{91}*$	1.000	0.119
x_{17}	$R00355MM$	x_{17}	0.985	0.000
x_{18}	$R00371MM$	x_{18}	0.997	0.000
x_{19}	$R00388MM$	$x_{16}, x_{19}, x_{39}, x_{107}$	1.000	0.048
x_{20}	$R00430MM$	$x_{20}*$	1.000	0.267
x_{21}	$R00432MM$	$x_8, x_{16}, x_{21}, x_{50}, x_{112}, x_{134}*$	1.000	0.119
x_{22}	$R00512MM$	$x_{22}, x_{63}, x_{129}**$	0.997	0.649
x_{23}	$R00551MM$	$x_9, x_{23}, x_{25}, x_{26}, x_{29}, x_{30}$	1.000	0.000
x_{24}	$R00572MM$	$x_{24}, x_{54}**$	0.997	0.654
x_{25}	$R00667MM$	$x_9, x_{23}, x_{25}, x_{26}, x_{29}, x_{30}$	1.000	0.000
x_{26}	$R00705MM$	$x_9, x_{23}, x_{25}, x_{26}, x_{29}, x_{30}$	1.000	n.a.
x_{27}	$R00709MM$	$x_{10}, x_{27}, x_{38}, x_{85}*$	1.000	0.119
x_{28}	$R00713MM$	$x_{28}*$	0.952	0.466
x_{29}	$R00716MM$	$x_9, x_{23}, x_{25}, x_{26}, x_{29}, x_{30}$	1.000	0.000
x_{30}	$R00740MM$	$x_9, x_{23}, x_{25}, x_{26}, x_{29}, x_{30}$	1.000	n.a.
x_{31}	$R00830MM$	$x_9, x_{23}, x_{25}, x_{26}, x_{29}, x_{31}$	1.000	n.a.
x_{32}	$R00833MM$	x_{32}	0.995	0.000
x_{33}	$R00851MM$	$x_{33}, x_{68}**$	0.965	0.691
x_{34}	$R00927MM$	$x_9, x_{23}, x_{25}, x_{26}, x_{29}, x_{34}$	1.000	n.a.
x_{35}	$R00941MM$	$x_9, x_{23}, x_{25}, x_{26}, x_{29}, x_{35}$	1.000	0.000
x_{36}	$R00945MM$	$x_9, x_{23}, x_{25}, x_{26}, x_{29}, x_{36}$	1.000	0.000
x_{37}	$R01082MM$	$x_8, x_{37}, x_{111}, x_{114}*$	1.000	0.123
x_{38}	$R01175MM$	$x_3, x_{13}, x_{27}, x_{38}, x_{108}*$	1.000	0.217
x_{39}	$R01177MM$	$x_{10}, x_{21}, x_{39}, x_{58}*$	1.000	0.217
x_{40}	$R01214MM$	$x_9, x_{23}, x_{25}, x_{26}, x_{29}, x_{40}$	1.000	0.000
x_{41}	$R01218MM$	$x_9, x_{23}, x_{25}, x_{26}, x_{29}, x_{41}$	1.000	0.000

Table D. Part I. Inflammation stage in the fumarase deficiency. Eight strong interdependencies have been found in this case.

Variable	Flux	Flux groups	r^2	cv
x_{42}	$R01253MM$	x_{42}	0.999	0.000
x_{43}	$R01279MM$	$x_{13}, x_{21}, x_{38}, x_{43}, x_{50}, x_{85}*$	1.000	0.217
x_{44}	$R01280MM$	$x_1, x_{24}, x_{44}*$	0.994	2.733
x_{45}	$R01325MM$	$x_{45}, x_{50}, x_{65}, x_{98}, x_{114}, x_{118}*$	1.000	0.119
x_{46}	$R01360MM$	$x_9, x_{23}, x_{25}, x_{26}, x_{29}, x_{46}$	1.000	n.a.
x_{47}	$R01361MM$	$x_9, x_{23}, x_{25}, x_{26}, x_{29}, x_{47}$	1.000	n.a.
x_{48}	$R01624MM$	$x_{48}, x_{97}**$	0.999	5.268
x_{49}	$R01626MM$	$x_{49}*$	0.982	1.322
x_{50}	$R01648MM$	$x_{50}, x_{104}*$	0.983	1.137
x_{51}	$R01655MM$	$x_9, x_{23}, x_{25}, x_{26}, x_{29}, x_{51}$	1.000	0.000
x_{52}	$R01700MM$	$x_{52}, x_{61}, x_{65}, x_{108}, x_{110}*$	1.000	0.119
x_{53}	$R01706MM$	$x_{53}*$	0.983	5.271
x_{54}	$R01799MM$	$x_{24}, x_{54}**$	0.997	0.657
x_{55}	$R01801MM$	x_{55}	n.a.	15.905
x_{56}	$R01859MM$	$x_9, x_{23}, x_{25}, x_{26}, x_{29}, x_{56}$	1.000	n.a.
x_{57}	$R01900MM$	$x_{39}, x_{50}, x_{57}, x_{58}, x_{88}, x_{90}*$	1.000	0.119
x_{58}	$R01923MM$	$x_{50}, x_{58}, x_{107}, x_{109}, x_{115}, x_{128}*$	1.000	0.217
x_{59}	$R01939MM$	$x_9, x_{23}, x_{25}, x_{26}, x_{29}, x_{59}$	1.000	0.000
x_{60}	$R01940MM$	$x_9, x_{23}, x_{25}, x_{26}, x_{29}, x_{60}$	1.000	0.000
x_{61}	$R01975MM$	$x_{50}, x_{52}, x_{61}, x_{86}, x_{107}, x_{117}*$	1.000	0.199
x_{62}	$R01978MM$	$x_9, x_{23}, x_{25}, x_{26}, x_{29}, x_{62}$	1.000	n.a.
x_{63}	$R02030MM$	$x_{22}, x_{63}, x_{129}**$	0.999	0.655
x_{64}	$R02161MM$	$x_{27}, x_{52}, x_{64}, x_{134}*$	1.000	0.115
x_{65}	$R02163MM$	$x_{65}, x_{117}, x_{118}, x_{134}*$	1.000	0.104
x_{66}	$R02164MM$	$x_{50}, x_{66}, x_{87}, x_{98}, x_{117}, x_{118}*$	1.000	0.119
x_{67}	$R02199MM$	$x_9, x_{23}, x_{25}, x_{26}, x_{29}, x_{67}$	1.000	n.a.
x_{68}	$R02241MM$	$x_{33}, x_{68}**$	0.965	0.676
x_{69}	$R02313MM$	$x_9, x_{23}, x_{25}, x_{26}, x_{29}, x_{69}$	1.000	0.000
x_{70}	$R02487MM$	$x_9, x_{23}, x_{25}, x_{26}, x_{29}, x_{70}$	1.000	0.000
x_{71}	$R02529MM$	x_{71}	n.a.	0.000
x_{72}	$R02569MM$	x_2, x_{72}, x_{82}	1.000	0.000
x_{73}	$R02570MM$	$x_{73}, x_{107}, x_{109}, x_{111}, x_{114}*$	1.000	0.119
x_{74}	$R02571MM$	$x_9, x_{23}, x_{25}, x_{26}, x_{29}, x_{74}$	1.000	0.000
x_{75}	$R02661MM$	$x_9, x_{23}, x_{25}, x_{26}, x_{29}, x_{75}$	1.000	0.000
x_{76}	$R02662MM$	$x_9, x_{23}, x_{25}, x_{26}, x_{29}, x_{76}$	1.000	0.000
x_{77}	$R02765MM$	$x_{77}*$	1.000	33.161
x_{78}	$R03026MM$	$x_{58}, x_{73}, x_{78}, x_{91}*$	1.000	0.199
x_{79}	$R03102MM$	$x_9, x_{23}, x_{25}, x_{26}, x_{29}, x_{79}$	1.000	0.000
x_{80}	$R03172MM$	x_{80}	0.836	3.329
x_{81}	$R03174MM$	$x_9, x_{23}, x_{25}, x_{26}, x_{29}, x_{81}$	1.000	n.a.
x_{82}	$R03270MM$	x_2, x_{72}, x_{82}	1.000	0.000
x_{83}	$R03314MM$	x_{83}	0.995	0.000
x_{84}	$R03381MM$	x_{84}	0.974	0.000
x_{85}	$R03777MM$	$x_{19}, x_{64}, x_{65}, x_{85}, x_{118}*$	1.000	0.217
x_{86}	$R03778MM$	$x_{19}, x_{50}, x_{58}, x_{78}, x_{86}, x_{104}*$	1.000	0.217
x_{87}	$R03857MM$	$x_3, x_{65}, x_{87}, x_{110}, x_{114}*$	1.000	0.217
x_{88}	$R03858MM$	$x_{39}, x_{43}, x_{73}, x_{88}, x_{114}*$	1.000	0.217

Table D. Part II. Inflammation stage in the fumarase deficiency.

Variable	Flux	Flux groups	r^2	cv
x_{89}	$R03990MM$	$x_4, x_{89}, x_{98}, x_{110}$*	1.000	0.217
x_{90}	$R03991MM$	$x_{16}, x_{90}, x_{91}, x_{109}, x_{114}$*	1.000	0.217
x_{91}	$R04170MM$	$x_{13}, x_{19}, x_{91}, x_{105}, x_{109}$*	1.000	0.217
x_{92}	$R04203MM$	$x_9, x_{23}, x_{25}, x_{26}, x_{29}, x_{92}$	1.000	n.a.
x_{93}	$R04204MM$	$x_9, x_{23}, x_{25}, x_{26}, x_{29}, x_{93}$	1.000	n.a.
x_{94}	$R04224MM$	$x_9, x_{23}, x_{25}, x_{26}, x_{29}, x_{94}$	1.000	0.000
x_{95}	$R04355MM$	x_{95}*	0.990	3.729
x_{96}	$R04428MM$	x_{96}, x_{99}**	0.999	2.360
x_{97}	$R04430MM$	x_{48}, x_{97}**	0.999	5.124
x_{98}	$R04433MM$	$x_4, x_{38}, x_{50}, x_{98}, x_{108}, x_{112}$*	1.000	0.213
x_{99}	$R04533MM$	x_{96}, x_{99}**	0.999	2.359
x_{100}	$R04536MM$	x_{100}, x_{102}*	0.992	2.531
x_{101}	$R04537MM$	x_{101}, x_{123}**	0.999	6.143
x_{102}	$R04543MM$	x_{102}, x_{120}**	0.992	1.294
x_{103}	$R04544MM$	x_{103}, x_{125}**	0.999	2.141
x_{104}	$R04737MM$	$x_3, x_{15}, x_{52}, x_{104}$*	1.000	0.217
x_{105}	$R04738MM$	$x_{37}, x_{50}, x_{87}, x_{90}, x_{105}, x_{111}$*	1.000	0.217
x_{106}	$R04739MM$	$x_{50}, x_{61}, x_{85}, x_{106}, x_{112}, x_{115}$*	1.000	0.217
x_{107}	$R04740MM$	$x_4, x_{39}, x_{88}, x_{107}$*	1.000	0.217
x_{108}	$R04741MM$	$x_{45}, x_{50}, x_{52}, x_{106}, x_{108}, x_{128}$*	1.000	0.217
x_{109}	$R04742MM$	$x_8, x_{50}, x_{88}, x_{90}, x_{107}, x_{109}$*	1.000	0.217
x_{110}	$R04743MM$	$x_3, x_{89}, x_{110}, x_{134}$*	1.000	0.217
x_{111}	$R04744MM$	$x_{19}, x_{50}, x_{52}, x_{58}, x_{88}, x_{111}$*	1.000	0.217
x_{112}	$R04745MM$	$x_{13}, x_{64}, x_{89}, x_{112}, x_{114}$*	1.000	0.217
x_{113}	$R04746MM$	$x_{37}, x_{50}, x_{88}, x_{98}, x_{113}, x_{114}$*	1.000	0.217
x_{114}	$R04747MM$	$x_4, x_{50}, x_{89}, x_{91}, x_{113}, x_{114}$*	1.000	0.217
x_{115}	$R04748MM$	$x_{85}, x_{89}, x_{104}, x_{115}$*	1.000	0.217
x_{116}	$R04749MM$	$x_{50}, x_{86}, x_{91}, x_{107}, x_{113}, x_{116}$*	1.000	0.217
x_{117}	$R04751MM$	$x_{10}, x_{64}, x_{87}, x_{117}$*	1.000	0.217
x_{118}	$R04754MM$	$x_4, x_{50}, x_{104}, x_{114}, x_{118}, x_{128}$*	1.000	0.217
x_{119}	$R04952MM$	x_{119}*	1.000	2.929
x_{120}	$R04953MM$	x_{102}, x_{120}**	0.992	2.379
x_{121}	$R04954MM$	x_{121}, x_{122}**	0.999	5.189
x_{122}	$R04956MM$	x_{121}, x_{122}**	0.999	5.189
x_{123}	$R04959MM$	x_{101}, x_{123}**	0.999	6.143
x_{124}	$R04968MM$	x_{121}, x_{124}*	0.979	1.594
x_{125}	$R04970MM$	x_{103}, x_{125}**	0.999	2.141
x_{126}	$R05064MM$	x_{47}, x_{126}	0.999	0.000
x_{127}	$R05066MM$	x_{31}, x_{127}	0.999	0.000
x_{128}	$R07162MM$	$x_{38}, x_{50}, x_{85}, x_{98}, x_{118}, x_{128}$	1.000	0.048
x_{129}	$R07390MM$	x_{22}, x_{63}, x_{129}**	0.998	0.655
x_{130}	$R07599MM$	$x_9, x_{23}, x_{25}, x_{26}, x_{29}, x_{130}$	1.000	0.000
x_{131}	$R07600MM$	$x_9, x_{23}, x_{25}, x_{26}, x_{29}, x_{131}$	1.000	0.000
x_{132}	$R07603MM$	$x_9, x_{23}, x_{25}, x_{26}, x_{29}, x_{132}$	1.000	n.a.
x_{133}	$R07604MM$	$x_9, x_{23}, x_{25}, x_{26}, x_{29}, x_{133}$	1.000	n.a.
x_{134}	$R07618MM$	$x_4, x_{10}, x_{61}, x_{118}, x_{134}$	1.000	0.082
x_{135}	$R08157MM$	$x_9, x_{23}, x_{25}, x_{26}, x_{29}, x_{135}$	1.000	0.000

Table D. Part III. Inflammation stage in the fumarase deficiency.

Variable	Flux	Flux groups	r^2	cv
x_1	$R00004MM$	$x_1, x_{22}, x_{33}, x_{54}, x_{112}*$	1.000	1.196
x_2	$R00014MM$	$x_2, x_{57}, x_{58}, x_{72}, x_{82}, x_{112}$	1.000	0.018
x_3	$R00081MM$	$x_3, x_{27}, x_{52}, x_{64}, x_{134}*$	1.000	0.502
x_4	$R00086MM$	$x_4, x_{61}, x_{108}, x_{115}, x_{134}*$	1.000	0.503
x_5	$R00127MM$	$x_5, x_{21}, x_{45}, x_{78}, x_{113}$	n.a.	0.658
x_6	$R00157MM$	$x_6, x_{68}*$	0.992	0.658
x_7	$R00205MM$	$x_7, x_{36}, x_{51}, x_{94}, x_{127}$	n.a.	0.000
x_8	$R00238MM$	$x_8, x_{105}, x_{109}, x_{111}, x_{116}*$	1.000	11.664
x_9	$R00243MM$	$x_9, x_{26}, x_{31}, x_{35}, x_{36}, x_{40}$	1.000	n.a.
x_{10}	$R00245MM$	$x_{10}, x_{104}, x_{110}*$	1.000	0.736
x_{11}	$R00256MM$	$x_{11}, x_{75}, x_{126}, x_{131}$	0.998	0.000
x_{12}	$R00258MM$	x_{12}, x_{42}	1.000	0.000
x_{13}	$R00275MM$	$x_{13}, x_{15}, x_{45}, x_{65}, x_{128}*$	1.000	0.476
x_{14}	$R00330MM$	$x_{14}, x_{19}, x_{39}*$	1.000	2.063
x_{15}	$R00342MM$	$x_{13}, x_{15}, x_{52}, x_{90}, x_{106}*$	1.000	0.275
x_{16}	$R00351MM$	$x_{16}, x_{57}, x_{86}, x_{109}, x_{114}*$	1.000	0.530
x_{17}	$R00355MM$	$x_1, x_{17}, x_{22}, x_{33}, x_{54}$	1.000	0.036
x_{18}	$R00371MM$	x_{18}	n.a.	0.000
x_{19}	$R00388MM$	$x_{16}, x_{19}, x_{57}, x_{106}, x_{116}*$	1.000	0.134
x_{20}	$R00430MM$	$x_{20}*$	1.000	2.055
x_{21}	$R00432MM$	$x_8, x_{21}, x_{57}, x_{105}, x_{116}*$	1.000	0.531
x_{22}	$R00512MM$	$x_1, x_{22}, x_{33}, x_{54}, x_{58}, x_{105}*$	1.000	1.196
x_{23}	$R00551MM$	$x_9, x_{23}, x_{40}, x_{69}, x_{94}$	0.999	0.000
x_{24}	$R00572MM$	$x_{15}, x_{19}, x_{24}, x_{65}, x_{128}*$	0.999	1.655
x_{25}	$R00667MM$	$x_{17}, x_{25}, x_{78}, x_{90}$	1.000	0.000
x_{26}	$R00705MM$	$x_9, x_{26}, x_{31}, x_{35}, x_{36}, x_{40}$	1.000	n.a.
x_{27}	$R00709MM$	$x_3, x_{15}, x_{27}, x_{104}, x_{115}*$	1.000	0.530
x_{28}	$R00713MM$	$x_{28}, x_{50}**$	0.999	4.942
x_{29}	$R00716MM$	x_{29}, x_{35}	1.000	0.000
x_{30}	$R00740MM$	$x_{30}, x_{31}*$	1.000	11.478
x_{31}	$R00830MM$	$x_9, x_{26}, x_{31}, x_{35}, x_{36}, x_{40}$	1.000	n.a.
x_{32}	$R00833MM$	x_{32}	1.000	0.000
x_{33}	$R00851MM$	$x_1, x_{22}, x_{33}, x_{54}, x_{78}*$	1.000	1.196
x_{34}	$R00927MM$	$x_{34}, x_{81}, x_{93}**$	1.000	824.829
x_{35}	$R00941MM$	$x_9, x_{26}, x_{31}, x_{35}, x_{36}, x_{40}$	1.000	0.000
x_{36}	$R00945MM$	$x_9, x_{26}, x_{31}, x_{35}, x_{36}, x_{40}$	1.000	0.000
x_{37}	$R01082MM$	$x_{19}, x_{37}, x_{66}, x_{73}, x_{78}*$	1.000	0.579
x_{38}	$R01175MM$	$x_{38}, x_{85}, x_{89}*$	1.000	1.401
x_{39}	$R01177MM$	$x_{13}, x_{19}, x_{21}, x_{39}, x_{114}*$	1.000	6.885
x_{40}	$R01214MM$	$x_9, x_{26}, x_{31}, x_{35}, x_{36}, x_{40}$	1.000	0.000
x_{41}	$R01218MM$	$x_{41}, x_{58}, x_{105}, x_{107}, x_{109}*$	1.000	1.056

Table E. Part I. Pathological stage in the fumarase deficiency. Only four functional groups have been detected two times, indicating that in the pathological stage there are less strong functional relations among the variables of the model.

Variable	Flux	Flux groups	r^2	cv
x_{42}	$R01253MM$	x_{12}, x_{42}	1.000	0.000
x_{43}	$R01279MM$	$x_{38}, x_{43}, x_{87}*$	1.000	1.401
x_{44}	$R01280MM$	$x_{44}, x_{49}, x_{95}*$	1.000	0.624
x_{45}	$R01325MM$	$x_{13}, x_{15}, x_{45}, x_{112}, x_{128}*$	1.000	0.530
x_{46}	$R01360MM$	$x_{46}, x_{62}*$	1.000	16.699
x_{47}	$R01361MM$	$x_{47}, x_{62}**$	1.000	17.001
x_{48}	$R01624MM$	$x_{48}, x_{103}*$	1.000	1.532
x_{49}	$R01626MM$	$x_{44}, x_{48}, x_{49}*$	1.000	1.574
x_{50}	$R01648MM$	$x_{28}, x_{50}**$	0.999	4.829
x_{51}	$R01655MM$	$x_9, x_{26}, x_{31}, x_{35}, x_{36}, x_{51}$	1.000	0.000
x_{52}	$R01700MM$	$x_{52}, x_{64}, x_{65}, x_{104}, x_{115}*$	1.000	0.533
x_{53}	$R01706MM$	$x_{53}, x_{121}*$	1.000	0.624
x_{54}	$R01799MM$	$x_1, x_{19}, x_{22}, x_{33}, x_{54}*$	1.000	1.196
x_{55}	$R01801MM$	x_{55}	n.a.	n.a.
x_{56}	$R01859MM$	$x_9, x_{26}, x_{31}, x_{35}, x_{36}, x_{56}$	1.000	n.a.
x_{57}	$R01900MM$	$x_{16}, x_{57}, x_{78}, x_{91}, x_{107}*$	1.000	0.530
x_{58}	$R01923MM$	$x_8, x_{21}, x_{58}, x_{90}, x_{115}$	n.a.	1.401
x_{59}	$R01939MM$	$x_9, x_{36}, x_{59}, x_{94}, x_{126}$	n.a.	0.000
x_{60}	$R01940MM$	$x_{31}, x_{35}, x_{36}, x_{60}, x_{76}$	n.a.	0.000
x_{61}	$R01975MM$	$x_{27}, x_{61}, x_{104}, x_{110}, x_{128}*$	1.000	11.664
x_{62}	$R01978MM$	$x_{47}, x_{62}**$	1.000	16.699
x_{63}	$R02030MM$	$x_4, x_{22}, x_{63}, x_{68}, x_{98}, x_{129}*$	1.000	1.196
x_{64}	$R02161MM$	$x_4, x_{45}, x_{57}, x_{64}, x_{66}*$	1.000	0.502
x_{65}	$R02163MM$	$x_{65}, x_{110}, x_{115}, x_{116}, x_{128}*$	1.000	0.476
x_{66}	$R02164MM$	$x_3, x_{27}, x_{61}, x_{66}, x_{108}*$	1.000	0.531
x_{67}	$R02199MM$	$x_{67}, x_{92}, x_{132}, x_{133}**$	1.000	n.a.
x_{68}	$R02241MM$	$x_{66}, x_{68}, x_{108}, x_{134}*$	1.000	1.196
x_{69}	$R02313MM$	x_{69}, x_{126}	1.000	0.000
x_{70}	$R02487MM$	$x_{26}, x_{40}, x_{51}, x_{70}, x_{76}$	n.a.	0.000
x_{71}	$R02529MM$	x_{71}	n.a.	0.000
x_{72}	$R02569MM$	$x_2, x_8, x_{58}, x_{72}, x_{82}, x_{91}$	1.000	0.018
x_{73}	$R02570MM$	$x_{21}, x_{73}, x_{88}, x_{107}, x_{111}*$	1.000	0.533
x_{74}	$R02571MM$	$x_{35}, x_{51}, x_{56}, x_{74}, x_{76}$	n.a.	0.000
x_{75}	$R02661MM$	$x_9, x_{26}, x_{31}, x_{35}, x_{36}, x_{75}$	1.000	0.000
x_{76}	$R02662MM$	$x_9, x_{26}, x_{31}, x_{35}, x_{36}, x_{76}$	1.000	0.000
x_{77}	$R02765MM$	$x_{77}, x_{105}, x_{107}*$	0.999	n.a.
x_{78}	$R03026MM$	$x_{16}, x_{73}, x_{78}, x_{88}, x_{111}*$	1.000	11.664
x_{79}	$R03102MM$	x_{79}, x_{131}	0.999	0.000
x_{80}	$R03172MM$	x_{80}	n.a.	5.398
x_{81}	$R03174MM$	$x_{34}, x_{81}, x_{93}**$	1.000	n.a.
x_{82}	$R03270MM$	$x_2, x_{72}, x_{82}, x_{98}, x_{104}, x_{112}$	1.000	0.018
x_{83}	$R03314MM$	x_{12}, x_{83}	1.000	0.000
x_{84}	$R03381MM$	$x_9, x_{26}, x_{31}, x_{35}, x_{36}, x_{84}$	1.000	0.000
x_{85}	$R03777MM$	$x_{85}, x_{117}*$	1.000	1.401
x_{86}	$R03778MM$	$x_{19}, x_{86}, x_{105}, x_{109}, x_{115}*$	1.000	6.885
x_{87}	$R03857MM$	$x_{85}, x_{87}*$	1.000	1.401
x_{88}	$R03858MM$	$x_{78}, x_{88}, x_{90}, x_{105}, x_{115}*$	1.000	6.885

Table E. Part II. Pathological stage in the fumarase deficiency.

Variable	Flux	Flux groups	r^2	cv
x_{89}	$R03990MM$	$x_{89}, x_{118}*$	1.000	1.401
x_{90}	$R03991MM$	$x_{19}, x_{21}, x_{78}, x_{90}, x_{111}*$	1.000	6.885
x_{91}	$R04170MM$	$x_{39}, x_{86}, x_{90}, x_{91}, x_{113}*$	1.000	6.885
x_{92}	$R04203MM$	$x_{67}, x_{92}, x_{132}, x_{133}**$	1.000	n.a.
x_{93}	$R04204MM$	$x_{34}, x_{81}, x_{93}**$	1.000	n.a.
x_{94}	$R04224MM$	$x_9, x_{26}, x_{31}, x_{35}, x_{36}, x_{94}$	1.000	0.000
x_{95}	$R04355MM$	$x_{48}, x_{95}*$	1.000	1.532
x_{96}	$R04428MM$	$x_{49}, x_{96}*$	1.000	1.532
x_{97}	$R04430MM$	$x_{97}, x_{123}*$	1.000	1.532
x_{98}	$R04433MM$	$x_3, x_{98}, x_{108}*$	1.000	1.233
x_{99}	$R04533MM$	$x_{99}, x_{120}*$	1.000	1.532
x_{100}	$R04536MM$	$x_{100}, x_{125}*$	1.000	1.532
x_{101}	$R04537MM$	$x_{53}, x_{101}, x_{103}*$	1.000	1.532
x_{102}	$R04543MM$	$x_{97}, x_{102}*$	1.000	0.624
x_{103}	$R04544MM$	$x_{53}, x_{103}*$	1.000	0.624
x_{104}	$R04737MM$	$x_{61}, x_{64}, x_{66}, x_{104}, x_{115}*$	1.000	6.885
x_{105}	$R04738MM$	$x_{39}, x_{78}, x_{86}, x_{105}, x_{116}*$	1.000	6.885
x_{106}	$R04739MM$	$x_3, x_{65}, x_{106}, x_{108}, x_{110}*$	1.000	6.885
x_{107}	$R04740MM$	$x_{39}, x_{86}, x_{107}, x_{114}, x_{116}*$	1.000	6.885
x_{108}	$R04741MM$	$x_4, x_{52}, x_{65}, x_{108}, x_{115}*$	1.000	6.885
x_{109}	$R04742MM$	$x_8, x_{91}, x_{105}, x_{109}, x_{114}*$	1.000	6.885
x_{110}	$R04743MM$	$x_{65}, x_{78}, x_{106}, x_{110}, x_{115}*$	1.000	6.885
x_{111}	$R04744MM$	$x_{73}, x_{78}, x_{105}, x_{109}, x_{111}*$	1.000	6.885
x_{112}	$R04745MM$	$x_3, x_4, x_{104}, x_{110}, x_{112}*$	1.000	6.885
x_{113}	$R04746MM$	$x_{73}, x_{90}, x_{107}, x_{113}, x_{116}*$	1.000	6.885
x_{114}	$R04747MM$	$x_{21}, x_{105}, x_{109}, x_{114}, x_{116}*$	1.000	6.885
x_{115}	$R04748MM$	$x_{61}, x_{65}, x_{66}, x_{112}, x_{115}*$	1.000	6.885
x_{116}	$R04749MM$	$x_8, x_{19}, x_{86}, x_{91}, x_{116}*$	1.000	6.885
x_{117}	$R04751MM$	$x_{117}, x_{118}*$	1.000	1.401
x_{118}	$R04754MM$	$x_{43}, x_{118}*$	1.000	1.401
x_{119}	$R04952MM$	$x_{119}, x_{124}*$	1.000	1.532
x_{120}	$R04953MM$	$x_{97}, x_{120}*$	1.000	1.532
x_{121}	$R04954MM$	$x_{95}, x_{96}, x_{121}*$	1.000	1.532
x_{122}	$R04956MM$	$x_{100}, x_{122}*$	1.000	1.532
x_{123}	$R04959MM$	$x_{100}, x_{102}, x_{120}, x_{122}, x_{123}*$	1.000	1.532
x_{124}	$R04968MM$	$x_{49}, x_{53}, x_{124}*$	1.000	0.624
x_{125}	$R04970MM$	$x_{120}, x_{125}*$	1.000	0.624
x_{126}	$R05064MM$	$x_9, x_{26}, x_{31}, x_{35}, x_{36}, x_{126}$	1.000	0.000
x_{127}	$R05066MM$	$x_9, x_{26}, x_{31}, x_{35}, x_{36}, x_{127}$	1.000	0.000
x_{128}	$R07162MM$	$x_{64}, x_{66}, x_{108}, x_{112}, x_{128}*$	1.000	0.134
x_{129}	$R07390MM$	$x_3, x_4, x_{63}, x_{64}, x_{129}*$	1.000	1.196
x_{130}	$R07599MM$	$x_9, x_{26}, x_{31}, x_{35}, x_{36}, x_{130}$	1.000	0.000
x_{131}	$R07600MM$	$x_9, x_{26}, x_{31}, x_{35}, x_{36}, x_{131}$	1.000	0.000
x_{132}	$R07603MM$	$x_{67}, x_{92}, x_{132}, x_{133}**$	1.000	n.a.
x_{133}	$R07604MM$	$x_{67}, x_{92}, x_{132}, x_{133}**$	1.000	n.a.
x_{134}	$R07618MM$	$x_3, x_{52}, x_{64}, x_{110}, x_{134}*$	1.000	0.264
x_{135}	$R08157MM$	$x_9, x_{26}, x_{31}, x_{35}, x_{36}, x_{135}$	1.000	0.000

Table E. Part III. Pathological stage in the fumarase deficiency.

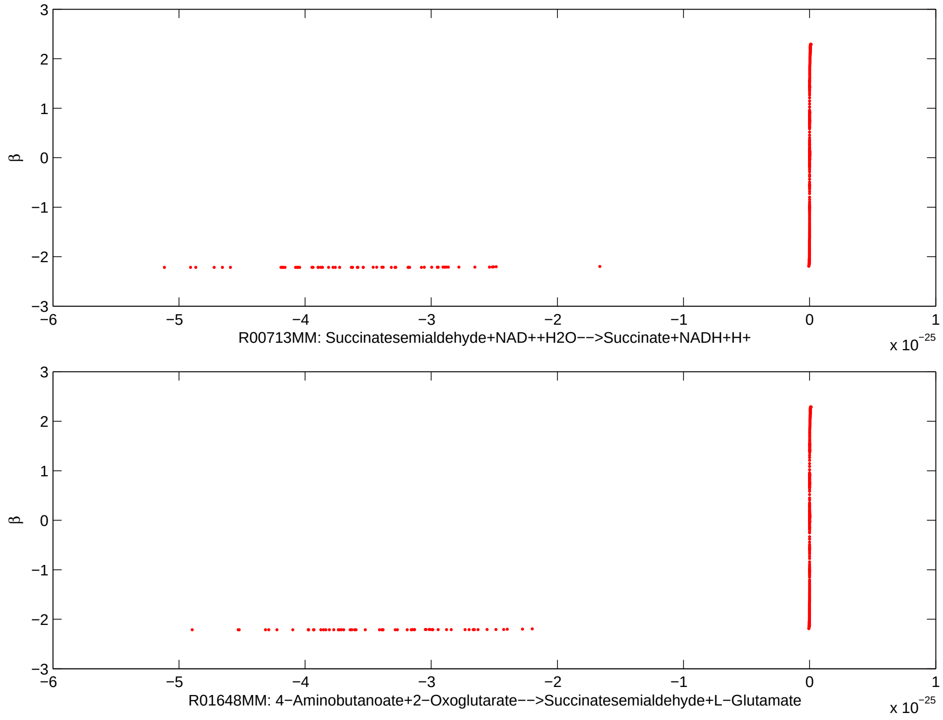

Figure S12. Fumarase deficiency - healthy stage. Optimal transformations β (y axis) found for the two fluxes R00713MM (top) and R01648MM (bottom) (x axis) [μmol min^{-1} gDW^{-1}] in the mitochondrial FBA model [2]. This plot proves that there is a strong relation between these two fluxes, with different outcome in the neighborhood of -5 and -1.7.

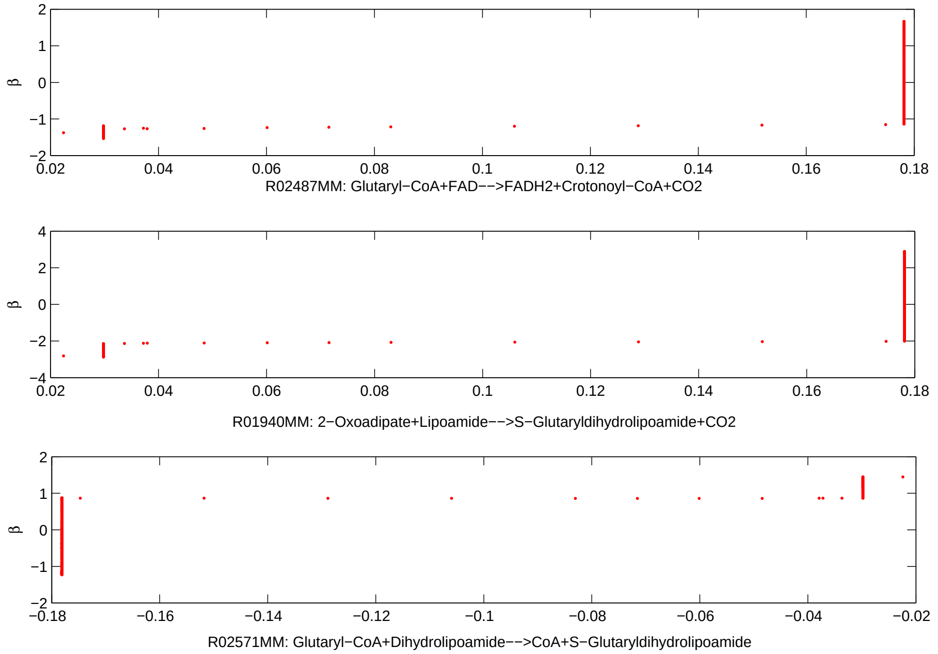

Figure S13. Fumarase deficiency - healthy stage. Optimal transformations β (y axis) found for the three fluxes R02487MM, R01940MM, and R02571MM (x axis) [μmol min^{-1} gDW^{-1}] in the mitochondrial FBA model [2].

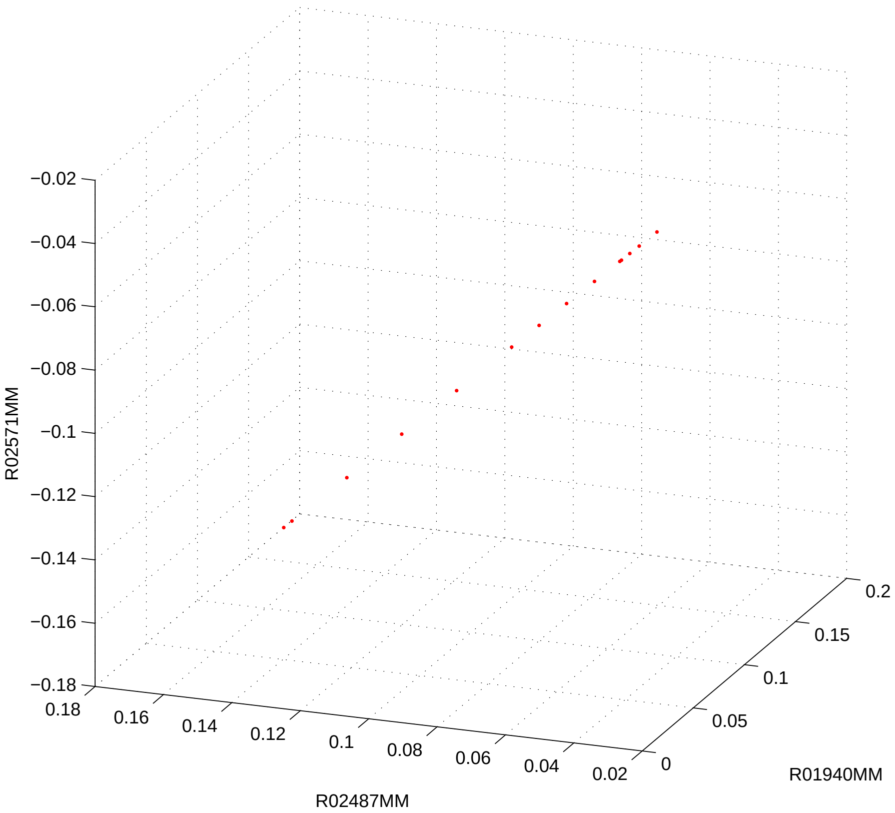

Figure S14. Fumarase deficiency - healthy stage. Functional relation found for the fluxes R02487MM, R01940MM, and R02571MM (x axis) [μmol min^{-1} gDW^{-1}] in the mitochondrial FBA model [2].

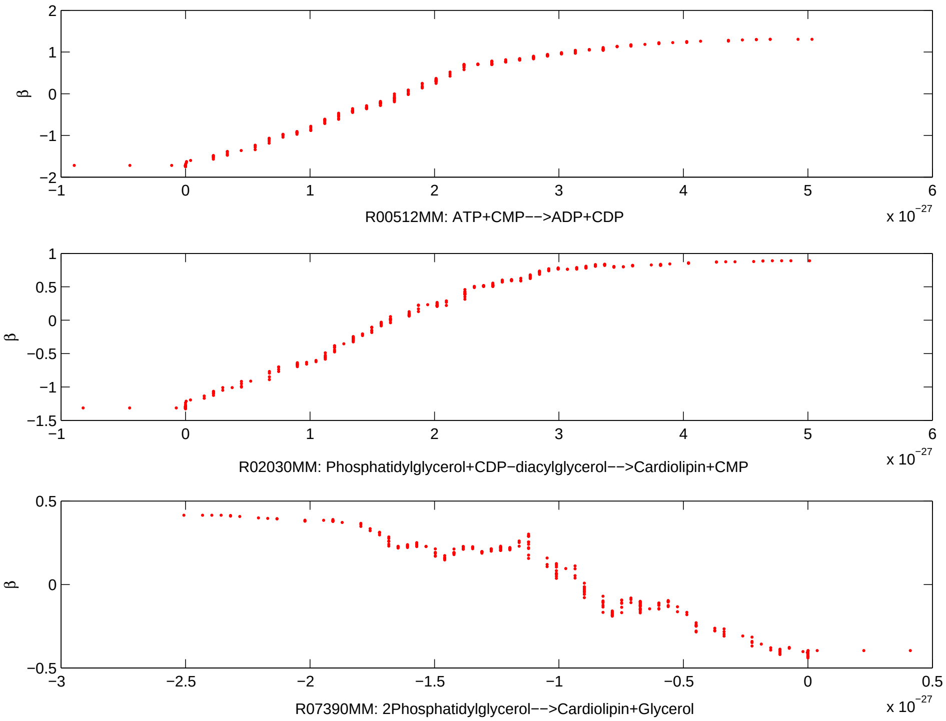

Figure S15. Fumarase deficiency - inflammation stage. Optimal transformations found for the three fluxes R00512MM, R02030MM, and R07390MM (x axis) [μmol min^{-1} gDW^{-1}] in the mitochondrial FBA model [2].

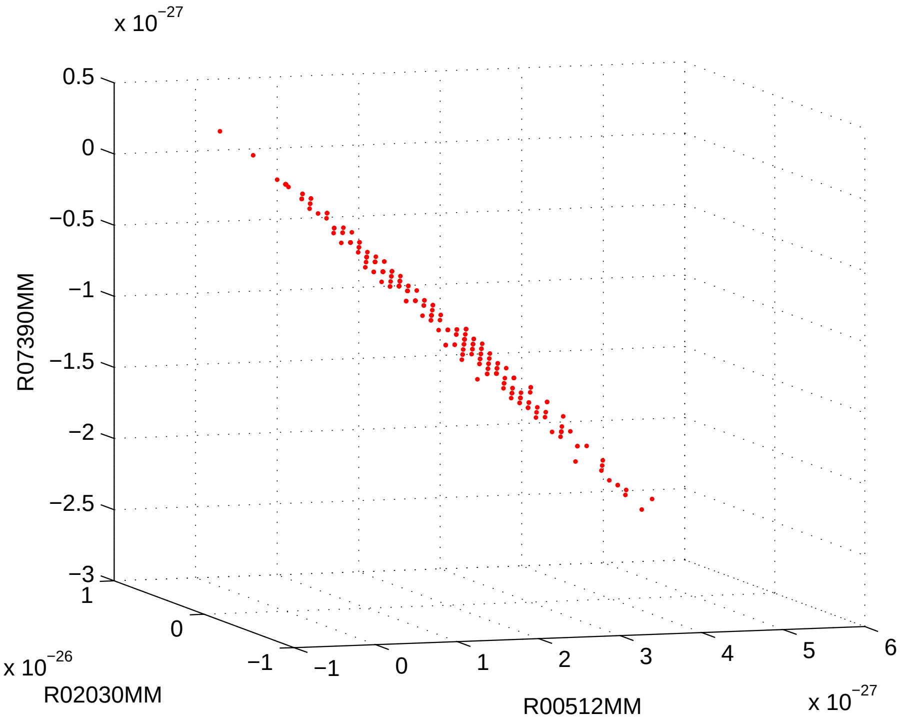

Figure S16. Fumarase deficiency - healthy stage. Functional relation found for the three fluxes R02030MM, R00512MM, and R07390M (x axis) [μmol min^{-1} gDW^{-1}] in the mitochondrial FBA model [2].

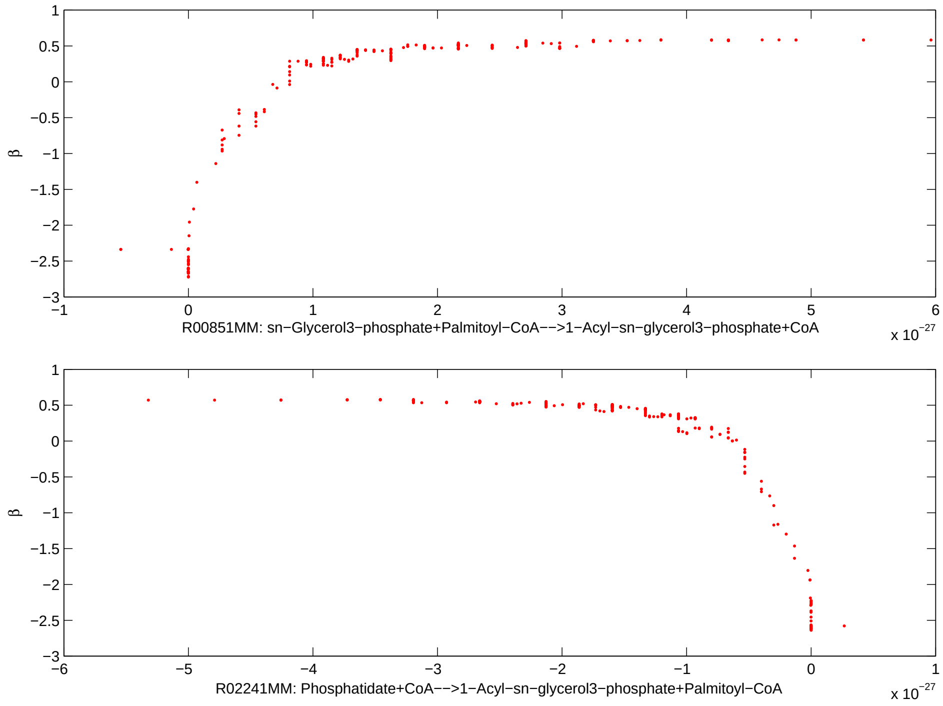

Figure S17. Fumarase deficiency - inflammation stage. Optimal transformations found for the two fluxes R00851MM and R02241MM (x axis) [μmol min^{-1} gDW^{-1}] in the mitochondrial FBA model [2].

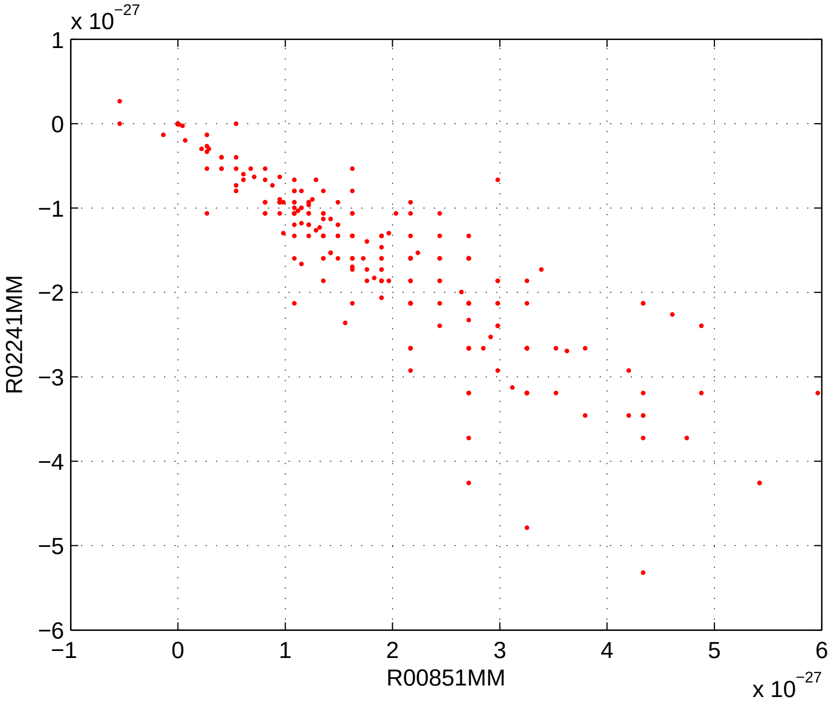

Figure S18. Fumarase deficiency - inflammation stage. Functional relation found for the two fluxes R00851MM and R02241MM (x axis) [μmol min^{-1} gDW^{-1}] in the mitochondrial FBA model [2].

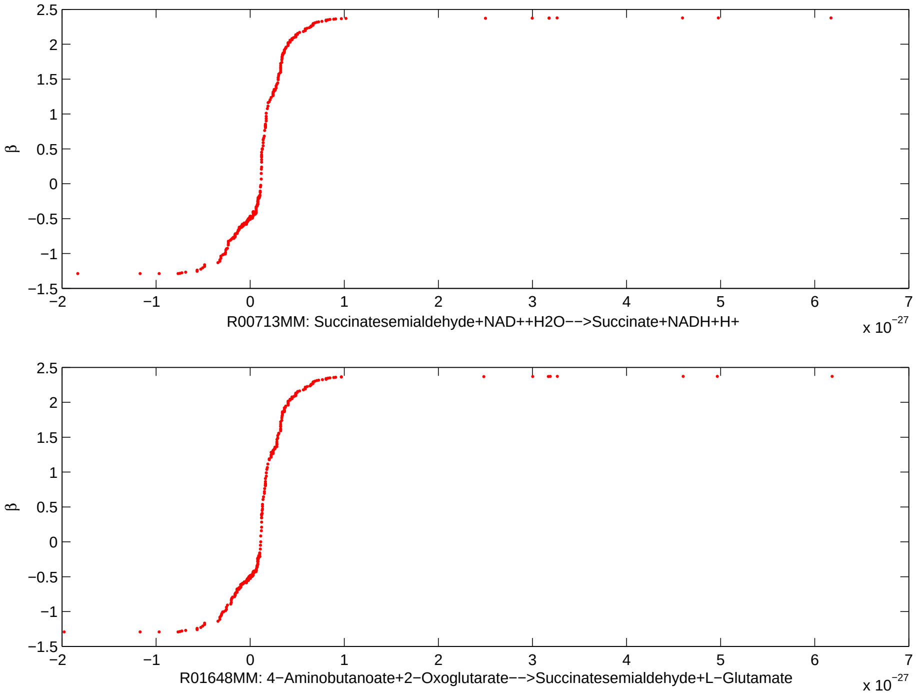

Figure S19. Fumarase deficiency - pathological stage. Optimal transformations found for the two fluxes R00713MM and R01648MM(x axis) [μmol min^{-1} gDW^{-1}] in the mitochondrial FBA model [2].

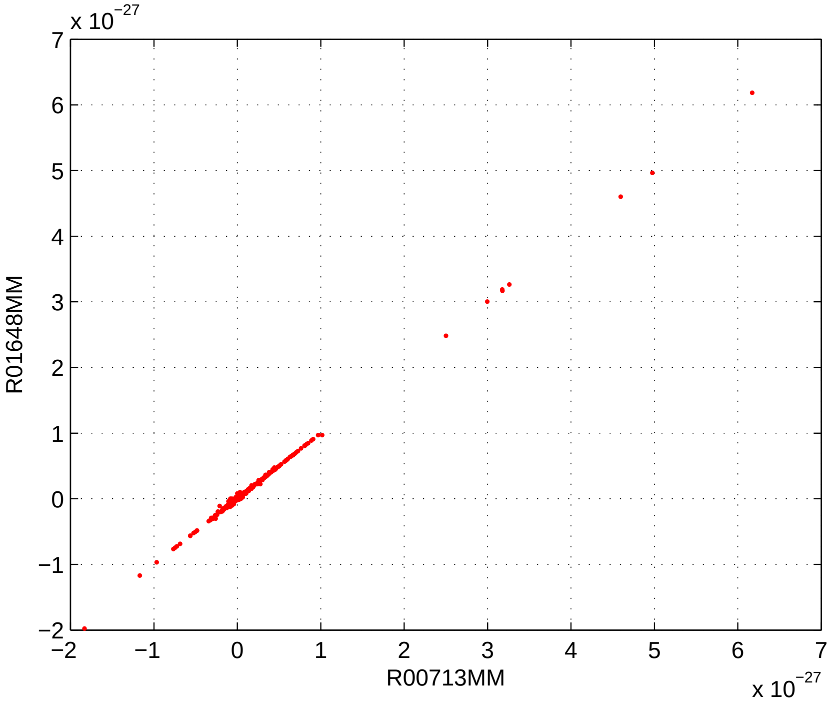

Figure S20. Fumarase deficiency - pathological stage. Functional relation found for the two fluxes R00713MM and R01648MM (x axis) [μmol min^{-1} gDW^{-1}] in the mitochondrial FBA model [2].

Variable	Flux	Flux groups	r^2	cv
x_1	$R00004MM$	$x_1, x_{15}, x_{19}, x_{45}, x_{78}$	n.a.	0.538
x_2	$R00014MM$	$x_2, x_{82}**$	1.000	0.820
x_3	$R00081MM$	x_3	-1.222	0.028
x_4	$R00086MM$	$x_4, x_{13}, x_{27}, x_{45}, x_{65}$	1.000	0.061
x_5	$R00127MM$	x_5*	1.000	0.538
x_6	$R00157MM$	$x_6, x_{88}, x_{114}, x_{116}*$	1.000	72.882
x_7	$R00205MM$	$x_7, x_{18}, x_{71}**$	1.000	2.044
x_8	$R00238MM$	$x_8, x_{21}, x_{37}, x_{57}, x_{73}**$	1.000	0.301
x_9	$R00243MM$	$x_9, x_{33}, x_{42}, x_{55}, x_{135}*$	1.000	3.688
x_{10}	$R00245MM$	$x_4, x_{10}, x_{13}, x_{65}, x_{128}*$	1.000	0.387
x_{11}	$R00256MM$	$x_7, x_{11}, x_{71}*$	0.998	2.585
x_{12}	$R00258MM$	$x_{12}, x_{60}, x_{70}*$	0.996	0.261
x_{13}	$R00275MM$	$x_4, x_{13}, x_{57}, x_{65}, x_{113}*$	1.000	0.110
x_{14}	$R00330MM$	$x_{13}, x_{14}, x_{20}*$	1.000	1.282
x_{15}	$R00342MM$	$x_{15}, x_{16}, x_{27}, x_{45}, x_{66}*$	1.000	0.166
x_{16}	$R00351MM$	$x_4, x_8, x_{16}, x_{27}, x_{57}*$	1.000	0.204
x_{17}	$R00355MM$	x_{17}, x_{74}	0.998	0.030
x_{18}	$R00371MM$	$x_7, x_{18}, x_{71}**$	1.000	2.044
x_{19}	$R00388MM$	$x_{19}, x_{105}, x_{107}, x_{111}, x_{128}*$	1.000	0.373
x_{20}	$R00430MM$	$x_{20}, x_{38}, x_{52}, x_{115}*$	0.999	1.315
x_{21}	$R00432MM$	$x_8, x_{16}, x_{21}, x_{37}, x_{73}*$	1.000	0.205
x_{22}	$R00512MM$	$x_{22}, x_{33}, x_{40}, x_{42}, x_{54}, x_{55}$	1.000	n.a.
x_{23}	$R00551MM$	x_{23}	0.999	0.000
x_{24}	$R00572MM$	$x_{15}, x_{24}, x_{45}, x_{52}, x_{66}$	n.a.	1.315
x_{25}	$R00667MM$	x_{25}	1.000	0.000
x_{26}	$R00705MM$	$x_{26}, x_{131}*$	1.000	30.545
x_{27}	$R00709MM$	$x_{15}, x_{27}, x_{45}, x_{66}, x_{134}*$	1.000	0.204
x_{28}	$R00713MM$	$x_{28}, x_{50}**$	0.999	3.121
x_{29}	$R00716MM$	$x_{19}, x_{29}, x_{37}, x_{59}, x_{69}, x_{128}*$	1.000	1.906
x_{30}	$R00740MM$	$x_{30}, x_{130}*$	1.000	30.545
x_{31}	$R00830MM$	$x_{31}*$	0.996	29.029
x_{32}	$R00833MM$	$x_{32}*$	1.000	0.112
x_{33}	$R00851MM$	$x_{22}, x_{33}, x_{40}, x_{42}, x_{54}, x_{55}$	1.000	n.a.
x_{34}	$R00927MM$	$x_{34}, x_{40}, x_{42}, x_{68}, x_{135}*$	0.997	0.491
x_{35}	$R00941MM$	$x_{22}, x_{35}, x_{54}, x_{130}*$	0.999	2.028
x_{36}	$R00945MM$	$x_{33}, x_{36}, x_{55}, x_{130}, x_{131}*$	1.000	2.028
x_{37}	$R01082MM$	$x_{16}, x_{21}, x_{37}, x_{57}, x_{73}*$	1.000	0.210
x_{38}	$R01175MM$	$x_{38}, x_{87}, x_{89}, x_{90}, x_{99}, x_{118}*$	0.999	0.281
x_{39}	$R01177MM$	$x_{39}, x_{86}, x_{88}, x_{111}, x_{113}*$	1.000	0.281
x_{40}	$R01214MM$	$x_{22}, x_{33}, x_{40}, x_{42}, x_{54}, x_{55}$	1.000	0.000
x_{41}	$R01218MM$	$x_{41}, x_{51}**$	1.000	2.028

Table F. Part I. Healthy stage in the succinate dehydrogenase deficiency. Ten functional groups have been detected by the algorithm.

Variable	Flux	Flux groups	r^2	cv
x_{42}	$R01253MM$	$x_{22}, x_{33}, x_{40}, x_{42}, x_{54}, x_{55}$	1.000	0.000
x_{43}	$R01279MM$	$x_{43}, x_{106}, x_{110}, x_{112}, x_{117}*$	1.000	0.281
x_{44}	$R01280MM$	$x_{44}, x_{96}*$	1.000	18.881
x_{45}	$R01325MM$	$x_{15}, x_{27}, x_{45}, x_{57}, x_{134}*$	1.000	0.204
x_{46}	$R01360MM$	x_{46}	-1.231	0.438
x_{47}	$R01361MM$	x_{47}	n.a.	0.438
x_{48}	$R01624MM$	$x_{10}, x_{48}*$	0.999	18.881
x_{49}	$R01626MM$	$x_{49}, x_{98}, x_{104}*$	0.999	18.881
x_{50}	$R01648MM$	$x_{28}, x_{50}**$	0.999	2.820
x_{51}	$R01655MM$	$x_{41}, x_{51}**$	1.000	2.028
x_{52}	$R01700MM$	$x_{15}, x_{21}, x_{27}, x_{52}, x_{66}*$	1.000	0.205
x_{53}	$R01706MM$	$x_{44}, x_{53}*$	1.000	18.881
x_{54}	$R01799MM$	$x_{22}, x_{33}, x_{40}, x_{42}, x_{54}, x_{55}$	1.000	n.a.
x_{55}	$R01801MM$	$x_{22}, x_{33}, x_{40}, x_{42}, x_{54}, x_{55}$	1.000	n.a.
x_{56}	$R01859MM$	$x_{56}, x_{131}*$	1.000	0.491
x_{57}	$R01900MM$	$x_{8}, x_{16}, x_{57}, x_{58}, x_{114}*$	1.000	0.204
x_{58}	$R01923MM$	$x_{58}, x_{86}, x_{111}, x_{113}, x_{116}*$	1.000	0.281
x_{59}	$R01939MM$	$x_{59}, x_{69}**$	1.000	1.906
x_{60}	$R01940MM$	$x_{60}, x_{70}, x_{74}**$	1.000	0.421
x_{61}	$R01975MM$	$x_{16}, x_{27}, x_{57}, x_{61}, x_{78}*$	1.000	0.273
x_{62}	$R01978MM$	$x_{15}, x_{62}, x_{102}, x_{109}, x_{113}, x_{125}$	n.a.	0.439
x_{63}	$R02030MM$	$x_{22}, x_{33}, x_{40}, x_{42}, x_{54}, x_{63}$	1.000	n.a.
x_{64}	$R02161MM$	x_{64}	n.a.	0.028
x_{65}	$R02163MM$	$x_{4}, x_{8}, x_{13}, x_{24}, x_{58}, x_{65}*$	1.000	0.110
x_{66}	$R02164MM$	$x_{15}, x_{21}, x_{52}, x_{66}, x_{134}*$	1.000	0.210
x_{67}	$R02199MM$	$x_{42}, x_{67}, x_{68}, x_{131}, x_{135}*$	1.000	0.491
x_{68}	$R02241MM$	$x_{22}, x_{33}, x_{40}, x_{42}, x_{54}, x_{68}$	1.000	n.a.
x_{69}	$R02313MM$	$x_{59}, x_{69}**$	1.000	1.906
x_{70}	$R02487MM$	$x_{60}, x_{70}, x_{74}**$	1.000	0.421
x_{71}	$R02529MM$	$x_{7}, x_{18}, x_{71}**$	1.000	2.044
x_{72}	$R02569MM$	$x_{2}, x_{15}, x_{19}, x_{72}, x_{78}, x_{134}$	n.a.	0.029
x_{73}	$R02570MM$	$x_{8}, x_{21}, x_{37}, x_{57}, x_{73}**$	1.000	0.205
x_{74}	$R02571MM$	$x_{60}, x_{70}, x_{74}**$	1.000	0.421
x_{75}	$R02661MM$	x_{40}, x_{75}	0.999	0.000
x_{76}	$R02662MM$	$x_{22}, x_{33}, x_{40}, x_{42}, x_{76}$	1.000	0.000
x_{77}	$R02765MM$	$x_{40}, x_{77}, x_{131}, x_{135}*$	0.999	0.491
x_{78}	$R03026MM$	$x_{19}, x_{45}, x_{61}, x_{78}, x_{128}*$	1.000	0.273
x_{79}	$R03102MM$	$x_{22}, x_{33}, x_{55}, x_{79}, x_{129}*$	0.999	1.906
x_{80}	$R03172MM$	$x_{80}*$	1.000	0.491
x_{81}	$R03174MM$	$x_{33}, x_{81}*$	1.000	0.491
x_{82}	$R03270MM$	$x_{2}, x_{82}**$	1.000	0.820
x_{83}	$R03314MM$	x_{40}, x_{83}	1.000	0.000
x_{84}	$R03381MM$	x_{40}, x_{84}	0.999	0.000
x_{85}	$R03777MM$	$x_{85}, x_{89}, x_{106}, x_{115}, x_{118}*$	1.000	0.281
x_{86}	$R03778MM$	$x_{39}, x_{86}, x_{90}, x_{111}, x_{114}*$	1.000	0.281
x_{87}	$R03857MM$	$x_{43}, x_{87}, x_{88}, x_{108}, x_{118}*$	1.000	0.281
x_{88}	$R03858MM$	$x_{39}, x_{88}, x_{105}, x_{109}, x_{113}*$	1.000	0.281

Table F. Part II. Healthy stage in the succinate dehydrogenase deficiency.

Variable	Flux	Flux groups	r^2	cv
x_{89}	$R03990MM$	$x_{43}, x_{89}, x_{112}, x_{115}, x_{117}$*	1.000	0.281
x_{90}	$R03991MM$	$x_{58}, x_{90}, x_{91}, x_{107}, x_{111}$*	1.000	0.281
x_{91}	$R04170MM$	$x_{58}, x_{86}, x_{91}, x_{105}, x_{116}$*	1.000	0.281
x_{92}	$R04203MM$	$x_{40}, x_{54}, x_{55}, x_{92}$*	1.000	0.491
x_{93}	$R04204MM$	x_{40}, x_{93}*	1.000	0.491
x_{94}	$R04224MM$	x_{42}, x_{94}	0.999	0.000
x_{95}	$R04355MM$	$x_{10}, x_{21}, x_{37}, x_{44}, x_{73}, x_{95}$*	0.998	18.881
x_{96}	$R04428MM$	x_{96}, x_{121}**	1.000	18.881
x_{97}	$R04430MM$	$x_{15}, x_{27}, x_{45}, x_{57}, x_{97}, x_{99}$	-1.115	18.881
x_{98}	$R04433MM$	$x_{38}, x_{98}, x_{105}, x_{113}, x_{114}$*	1.000	0.277
x_{99}	$R04533MM$	x_{21}, x_{99}*	0.999	18.881
x_{100}	$R04536MM$	x_{100}, x_{122}**	1.000	18.881
x_{101}	$R04537MM$	x_{96}, x_{101}*	1.000	18.881
x_{102}	$R04543MM$	x_{102}, x_{125}**	1.000	18.881
x_{103}	$R04544MM$	x_{44}, x_{103}*	1.000	18.881
x_{104}	$R04737MM$	$x_{86}, x_{87}, x_{104}, x_{108}, x_{115}$*	1.000	0.281
x_{105}	$R04738MM$	$x_{43}, x_{86}, x_{88}, x_{105}, x_{113}$*	1.000	0.281
x_{106}	$R04739MM$	$x_{43}, x_{87}, x_{106}, x_{110}, x_{118}$*	1.000	0.281
x_{107}	$R04740MM$	$x_{39}, x_{90}, x_{105}, x_{107}, x_{116}$*	1.000	0.281
x_{108}	$R04741MM$	$x_{43}, x_{104}, x_{108}, x_{112}, x_{118}$*	1.000	0.281
x_{109}	$R04742MM$	$x_{39}, x_{90}, x_{91}, x_{109}, x_{114}$*	1.000	0.281
x_{110}	$R04743MM$	$x_{87}, x_{89}, x_{104}, x_{110}, x_{115}$*	1.000	0.281
x_{111}	$R04744MM$	$x_{88}, x_{111}, x_{113}, x_{114}, x_{116}$*	1.000	0.281
x_{112}	$R04745MM$	$x_{85}, x_{104}, x_{110}, x_{112}, x_{116}$*	1.000	0.281
x_{113}	$R04746MM$	$x_{86}, x_{91}, x_{105}, x_{113}, x_{116}$*	1.000	0.281
x_{114}	$R04747MM$	$x_{39}, x_{90}, x_{91}, x_{111}, x_{114}$*	1.000	0.281
x_{115}	$R04748MM$	$x_{85}, x_{106}, x_{108}, x_{115}, x_{118}$*	1.000	0.281
x_{116}	$R04749MM$	$x_{39}, x_{90}, x_{105}, x_{106}, x_{116}$*	1.000	0.281
x_{117}	$R04751MM$	$x_{43}, x_{87}, x_{112}, x_{117}, x_{118}$*	1.000	0.281
x_{118}	$R04754MM$	$x_{43}, x_{85}, x_{89}, x_{112}, x_{118}$*	1.000	0.281
x_{119}	$R04952MM$	x_{48}, x_{119}*	1.000	18.881
x_{120}	$R04953MM$	x_{102}, x_{120}*	1.000	18.881
x_{121}	$R04954MM$	x_{96}, x_{121}**	1.000	18.881
x_{122}	$R04956MM$	x_{100}, x_{122}**	1.000	18.881
x_{123}	$R04959MM$	x_{120}, x_{123}*	1.000	18.881
x_{124}	$R04968MM$	x_{53}, x_{124}*	1.000	18.881
x_{125}	$R04970MM$	x_{102}, x_{125}**	1.000	18.881
x_{126}	$R05064MM$	x_{42}, x_{126}	0.999	0.000
x_{127}	$R05066MM$	x_{55}, x_{127}	1.000	0.000
x_{128}	$R07162MM$	$x_{19}, x_{90}, x_{91}, x_{114}, x_{128}$*	1.000	0.373
x_{129}	$R07390MM$	$x_{22}, x_{33}, x_{40}, x_{42}, x_{54}, x_{129}$	1.000	n.a.
x_{130}	$R07599MM$	$x_{22}, x_{33}, x_{40}, x_{42}, x_{54}, x_{130}$	1.000	0.000
x_{131}	$R07600MM$	$x_{22}, x_{33}, x_{40}, x_{42}, x_{54}, x_{131}$	1.000	0.000
x_{132}	$R07603MM$	$x_{68}, x_{130}, x_{132}, x_{135}$*	1.000	0.491
x_{133}	$R07604MM$	$x_{63}, x_{68}, x_{129}, x_{133}, x_{135}$*	1.000	0.491
x_{134}	$R07618MM$	$x_{27}, x_{37}, x_{52}, x_{66}, x_{134}$*	1.000	0.168
x_{135}	$R08157MM$	$x_{22}, x_{33}, x_{40}, x_{42}, x_{54}, x_{135}$	1.000	0.000

Table F. Part III. Healthy stage in the succinate dehydrogenase deficiency.

Variable	Flux	Flux groups	r^2	cv
x_1	$R00004MM$	$x_1, x_{44}, x_{53}, x_{103}, x_{124}$**	0.998	13.281
x_2	$R00014MM$	$x_2, x_{33}, x_{41}, x_{82}$*	1.000	1.969
x_3	$R00081MM$	$x_3, x_4, x_{10}, x_{128}$*	1.000	0.116
x_4	$R00086MM$	$x_4, x_{15}, x_{37}, x_{115}$*	1.000	0.113
x_5	$R00127MM$	x_5*	0.984	11.428
x_6	$R00157MM$	x_6*	0.999	11.428
x_7	$R00205MM$	x_7	0.973	0.000
x_8	$R00238MM$	$x_8, x_{39}, x_{43}, x_{90}, x_{91}$*	1.000	0.199
x_9	$R00243MM$	$x_9, x_{22}, x_{26}, x_{29}, x_{30}, x_{31}$	1.000	n.a.
x_{10}	$R00245MM$	$x_3, x_{10}, x_{106}, x_{128}$	1.000	0.032
x_{11}	$R00256MM$	x_{11}	0.995	0.000
x_{12}	$R00258MM$	x_{12}	0.000	0.000
x_{13}	$R00275MM$	$x_4, x_{13}, x_{43}, x_{64}, x_{112}$*	1.000	0.104
x_{14}	$R00330MM$	x_{14}*	1.000	0.250
x_{15}	$R00342MM$	$x_{15}, x_{43}, x_{104}, x_{110}, x_{115}$	1.000	0.082
x_{16}	$R00351MM$	$x_{16}, x_{39}, x_{57}, x_{58}, x_{113}$*	1.000	0.119
x_{17}	$R00355MM$	x_{17}, x_{60}	0.999	0.000
x_{18}	$R00371MM$	x_{18}	0.997	0.000
x_{19}	$R00388MM$	$x_{19}, x_{37}, x_{52}, x_{109}, x_{116}$	1.000	0.048
x_{20}	$R00430MM$	$x_{20}, x_{106}, x_{109}, x_{118}, x_{134}$*	1.000	0.251
x_{21}	$R00432MM$	$x_{21}, x_{39}, x_{57}, x_{91}, x_{111}$*	1.000	0.119
x_{22}	$R00512MM$	$x_9, x_{22}, x_{26}, x_{29}, x_{30}, x_{31}$	1.000	n.a.
x_{23}	$R00551MM$	x_{23}, x_{31}, x_{40}	0.999	0.000
x_{24}	$R00572MM$	$x_{20}, x_{24}, x_{45}, x_{88}$*	1.000	0.251
x_{25}	$R00667MM$	x_{25}, x_{33}, x_{60}	0.999	0.000
x_{26}	$R00705MM$	$x_9, x_{22}, x_{26}, x_{29}, x_{30}, x_{31}$	1.000	n.a.
x_{27}	$R00709MM$	$x_4, x_{27}, x_{87}, x_{106}$*	1.000	0.119
x_{28}	$R00713MM$	x_{28}, x_{50}**	1.000	3.994
x_{29}	$R00716MM$	$x_9, x_{22}, x_{26}, x_{29}, x_{30}, x_{31}$	1.000	0.000
x_{30}	$R00740MM$	$x_9, x_{22}, x_{26}, x_{29}, x_{30}, x_{31}$	1.000	n.a.
x_{31}	$R00830MM$	$x_9, x_{22}, x_{26}, x_{29}, x_{30}, x_{31}$	1.000	n.a.
x_{32}	$R00833MM$	$x_9, x_{22}, x_{26}, x_{29}, x_{32}$	0.999	0.000
x_{33}	$R00851MM$	$x_9, x_{22}, x_{26}, x_{29}, x_{30}, x_{33}$	1.000	n.a.
x_{34}	$R00927MM$	x_{34}, x_{81}*	1.000	6.807
x_{35}	$R00941MM$	$x_9, x_{22}, x_{26}, x_{29}, x_{30}, x_{35}$	1.000	0.000
x_{36}	$R00945MM$	$x_9, x_{22}, x_{26}, x_{29}, x_{30}, x_{36}$	1.000	0.000
x_{37}	$R01082MM$	$x_{19}, x_{37}, x_{57}, x_{116}$*	1.000	0.123
x_{38}	$R01175MM$	$x_{38}, x_{64}, x_{85}, x_{110}, x_{112}$*	1.000	0.217
x_{39}	$R01177MM$	$x_{38}, x_{39}, x_{98}, x_{107}, x_{109}$*	1.000	0.217
x_{40}	$R01214MM$	$x_9, x_{22}, x_{26}, x_{29}, x_{30}, x_{40}$	1.000	0.000
x_{41}	$R01218MM$	$x_9, x_{22}, x_{26}, x_{29}, x_{30}, x_{41}$	1.000	0.000

Table G. Part I. Inflammation stage in the succinate dehydrogenase deficiency. Although there are many functional groups with four or five reactions, only three groups show strong functional relations.

Variable	Flux	Flux groups	r^2	cv
x_{42}	$R01253MM$	$x_9, x_{22}, x_{26}, x_{29}, x_{30}, x_{42}$	1.000	0.000
x_{43}	$R01279MM$	$x_3, x_{43}, x_{66}, x_{108}*$	1.000	0.217
x_{44}	$R01280MM$	$x_1, x_{44}, x_{53}, x_{103}*$	0.999	13.325
x_{45}	$R01325MM$	$x_{19}, x_{43}, x_{45}, x_{61}, x_{87}*$	1.000	0.119
x_{46}	$R01360MM$	$x_{46}, x_{47}, x_{62}**$	1.000	1.614
x_{47}	$R01361MM$	$x_{46}, x_{47}, x_{62}**$	1.000	1.618
x_{48}	$R01624MM$	$x_{48}, x_{95}*$	0.998	7.263
x_{49}	$R01626MM$	$x_{49}, x_{122}*$	0.990	3.750
x_{50}	$R01648MM$	$x_{28}, x_{50}**$	1.000	3.994
x_{51}	$R01655MM$	$x_9, x_{22}, x_{26}, x_{29}, x_{30}, x_{51}$	1.000	0.000
x_{52}	$R01700MM$	$x_3, x_{52}, x_{64}, x_{108}*$	1.000	0.119
x_{53}	$R01706MM$	$x_1, x_{44}, x_{53}, x_{103}, x_{124}**$	0.998	13.285
x_{54}	$R01799MM$	$x_9, x_{22}, x_{26}, x_{29}, x_{30}, x_{54}$	1.000	n.a.
x_{55}	$R01801MM$	$x_9, x_{22}, x_{26}, x_{29}, x_{30}, x_{55}$	1.000	n.a.
x_{56}	$R01859MM$	$x_9, x_{22}, x_{26}, x_{29}, x_{30}, x_{56}$	1.000	n.a.
x_{57}	$R01900MM$	$x_{39}, x_{57}, x_{90}, x_{109}, x_{116}*$	1.000	0.119
x_{58}	$R01923MM$	$x_{16}, x_{37}, x_{38}, x_{58}, x_{90}*$	1.000	0.217
x_{59}	$R01939MM$	$x_{26}, x_{51}, x_{59}, x_{135}$	0.998	0.000
x_{60}	$R01940MM$	$x_9, x_{22}, x_{26}, x_{29}, x_{30}, x_{60}$	1.000	0.000
x_{61}	$R01975MM$	$x_{61}, x_{85}, x_{106}, x_{110}, x_{134}*$	1.000	0.199
x_{62}	$R01978MM$	$x_{46}, x_{47}, x_{62}**$	1.000	1.614
x_{63}	$R02030MM$	$x_9, x_{22}, x_{26}, x_{29}, x_{30}, x_{63}$	1.000	n.a.
x_{64}	$R02161MM$	$x_{10}, x_{64}, x_{89}, x_{104}*$	1.000	0.116
x_{65}	$R02163MM$	$x_{27}, x_{65}, x_{90}, x_{112}, x_{128}*$	1.000	0.104
x_{66}	$R02164MM$	$x_{13}, x_{27}, x_{66}, x_{114}, x_{134}*$	1.000	0.123
x_{67}	$R02199MM$	$x_{67}, x_{132}*$	1.000	6.807
x_{68}	$R02241MM$	$x_9, x_{22}, x_{26}, x_{29}, x_{30}, x_{68}$	1.000	n.a.
x_{69}	$R02313MM$	$x_9, x_{22}, x_{26}, x_{29}, x_{30}, x_{69}$	1.000	0.000
x_{70}	$R02487MM$	x_{51}, x_{70}	1.000	0.000
x_{71}	$R02529MM$	x_{71}	0.989	0.000
x_{72}	$R02569MM$	x_{72}	0.881	0.000
x_{73}	$R02570MM$	$x_{37}, x_{73}, x_{86}, x_{105}, x_{112}*$	1.000	0.119
x_{74}	$R02571MM$	$x_9, x_{22}, x_{26}, x_{29}, x_{74}$	1.000	0.000
x_{75}	$R02661MM$	x_{31}, x_{40}, x_{75}	1.000	0.000
x_{76}	$R02662MM$	$x_9, x_{22}, x_{26}, x_{29}, x_{76}$	1.000	0.000
x_{77}	$R02765MM$	$x_9, x_{22}, x_{26}, x_{29}, x_{30}, x_{77}$	1.000	n.a.
x_{78}	$R03026MM$	$x_{19}, x_{58}, x_{78}, x_{111}, x_{116}*$	1.000	0.199
x_{79}	$R03102MM$	$x_9, x_{22}, x_{26}, x_{29}, x_{79}$	0.996	0.000
x_{80}	$R03172MM$	$x_{80}*$	0.994	n.a.
x_{81}	$R03174MM$	$x_{34}, x_{81}, x_{93}*$	1.000	6.807
x_{82}	$R03270MM$	$x_2, x_9, x_{42}, x_{55}, x_{82}*$	1.000	1.969
x_{83}	$R03314MM$	$x_9, x_{22}, x_{26}, x_{29}, x_{30}, x_{83}$	1.000	0.000
x_{84}	$R03381MM$	x_{51}, x_{83}, x_{84}	1.000	0.000
x_{85}	$R03777MM$	$x_{64}, x_{66}, x_{85}, x_{118}*$	1.000	0.217
x_{86}	$R03778MM$	$x_{64}, x_{73}, x_{86}, x_{109}*$	1.000	0.217
x_{87}	$R03857MM$	$x_{65}, x_{85}, x_{87}, x_{110}*$	1.000	0.217
x_{88}	$R03858MM$	$x_{39}, x_{57}, x_{78}, x_{88}*$	1.000	0.217

Table G. Part II. Inflammation stage in the succinate dehydrogenase deficiency.

Variable	Flux	Flux groups	r^2	cv
x_{89}	$R03990MM$	$x_3, x_{57}, x_{89}, x_{98}, x_{118}$*	1.000	0.217
x_{90}	$R03991MM$	$x_8, x_{39}, x_{57}, x_{90}$*	1.000	0.217
x_{91}	$R04170MM$	$x_{37}, x_{58}, x_{78}, x_{91}$*	1.000	0.217
x_{92}	$R04203MM$	x_{67}, x_{92}, x_{133}*	1.000	6.807
x_{93}	$R04204MM$	x_{81}, x_{93}*	1.000	6.807
x_{94}	$R04224MM$	x_{94}, x_{129}	1.000	0.000
x_{95}	$R04355MM$	x_{95}, x_{101}, x_{123}*	0.998	0.521
x_{96}	$R04428MM$	$x_{96}, x_{101}, x_{121}, x_{123}$*	1.000	7.370
x_{97}	$R04430MM$	$x_{97}, x_{99}, x_{101}, x_{122}, x_{123}$*	0.998	7.370
x_{98}	$R04433MM$	$x_{98}, x_{108}, x_{112}, x_{117}, x_{118}$*	1.000	0.213
x_{99}	$R04533MM$	$x_{49}, x_{97}, x_{99}, x_{119}, x_{122}, x_{123}$*	0.997	7.370
x_{100}	$R04536MM$	$x_{100}, x_{101}, x_{120}, x_{123}$*	1.000	7.380
x_{101}	$R04537MM$	$x_{96}, x_{101}, x_{120}, x_{121}$*	1.000	7.380
x_{102}	$R04543MM$	$x_{100}, x_{102}, x_{103}, x_{124}, x_{125}$*	1.000	13.300
x_{103}	$R04544MM$	$x_{44}, x_{53}, x_{103}, x_{124}, x_{125}$*	0.998	13.300
x_{104}	$R04737MM$	$x_{85}, x_{89}, x_{104}, x_{106}, x_{110}$*	1.000	0.217
x_{105}	$R04738MM$	$x_8, x_{90}, x_{105}, x_{110}$*	1.000	0.217
x_{106}	$R04739MM$	$x_{73}, x_{89}, x_{98}, x_{106}, x_{112}$*	1.000	0.217
x_{107}	$R04740MM$	$x_{91}, x_{105}, x_{106}, x_{107}, x_{116}$*	1.000	0.217
x_{108}	$R04741MM$	$x_{39}, x_{108}, x_{112}, x_{116}, x_{117}$*	1.000	0.217
x_{109}	$R04742MM$	$x_{91}, x_{109}, x_{110}, x_{114}, x_{117}$*	1.000	0.217
x_{110}	$R04743MM$	$x_4, x_{87}, x_{110}, x_{134}$*	1.000	0.217
x_{111}	$R04744MM$	$x_{78}, x_{110}, x_{111}, x_{113}, x_{116}$*	1.000	0.217
x_{112}	$R04745MM$	$x_{65}, x_{66}, x_{98}, x_{112}$*	1.000	0.217
x_{113}	$R04746MM$	$x_{16}, x_{37}, x_{57}, x_{113}$*	1.000	0.217
x_{114}	$R04747MM$	$x_{43}, x_{91}, x_{107}, x_{114}$*	1.000	0.217
x_{115}	$R04748MM$	$x_3, x_{27}, x_{38}, x_{115}$*	1.000	0.217
x_{116}	$R04749MM$	$x_{73}, x_{86}, x_{113}, x_{115}, x_{116}$*	1.000	0.217
x_{117}	$R04751MM$	$x_{38}, x_{45}, x_{98}, x_{115}, x_{117}$*	1.000	0.217
x_{118}	$R04754MM$	$x_3, x_{115}, x_{118}, x_{134}$*	1.000	0.217
x_{119}	$R04952MM$	$x_{96}, x_{101}, x_{119}, x_{121}$**	1.000	7.372
x_{120}	$R04953MM$	$x_{49}, x_{99}, x_{100}, x_{119}, x_{120}, x_{123}$*	0.997	7.375
x_{121}	$R04954MM$	$x_{96}, x_{101}, x_{119}, x_{121}$**	1.000	7.375
x_{122}	$R04956MM$	$x_{97}, x_{120}, x_{122}, x_{123}$*	1.000	7.375
x_{123}	$R04959MM$	$x_{99}, x_{100}, x_{101}, x_{122}, x_{123}$*	1.000	7.380
x_{124}	$R04968MM$	$x_{96}, x_{101}, x_{119}, x_{124}$*	1.000	13.262
x_{125}	$R04970MM$	$x_{44}, x_{102}, x_{103}, x_{122}, x_{125}$*	0.999	13.300
x_{126}	$R05064MM$	x_{126}, x_{131}	1.000	0.000
x_{127}	$R05066MM$	x_{127}	1.000	0.000
x_{128}	$R07162MM$	$x_3, x_{43}, x_{108}, x_{112}, x_{128}$	1.000	0.048
x_{129}	$R07390MM$	$x_9, x_{22}, x_{26}, x_{29}, x_{30}, x_{129}$	1.000	n.a.
x_{130}	$R07599MM$	$x_9, x_{22}, x_{26}, x_{29}, x_{30}, x_{130}$	1.000	0.000
x_{131}	$R07600MM$	$x_9, x_{22}, x_{26}, x_{29}, x_{30}, x_{131}$	1.000	0.000
x_{132}	$R07603MM$	x_{67}, x_{92}, x_{132}*	1.000	6.807
x_{133}	$R07604MM$	$x_{67}, x_{92}, x_{132}, x_{133}$*	1.000	6.807
x_{134}	$R07618MM$	$x_{43}, x_{66}, x_{118}, x_{134}$	1.000	0.082
x_{135}	$R08157MM$	$x_9, x_{22}, x_{26}, x_{29}, x_{30}, x_{135}$	1.000	0.000

Table G. Part III. Inflammation stage in the succinate dehydrogenase deficiency.

Variable	Flux	Flux groups	r^2	cv
x_1	$R00004MM$	$x_1, x_{91}, x_{106}*$	1.000	1.604
x_2	$R00014MM$	$x_2, x_{82}**$	1.000	0.800
x_3	$R00081MM$	$x_3, x_{64}*$	1.000	0.924
x_4	$R00086MM$	$x_4, x_{64}*$	1.000	0.922
x_5	$R00127MM$	$x_5, x_{103}*$	1.000	2.565
x_6	$R00157MM$	$x_6, x_9, x_{26}, x_{55}, x_{77}*$	0.998	2.565
x_7	$R00205MM$	$x_7, x_{40}, x_{42}, x_{131}*$	1.000	0.687
x_8	$R00238MM$	$x_8, x_{61}, x_{78}*$	1.000	14.259
x_9	$R00243MM$	$x_9, x_{26}, x_{30}, x_{55}, x_{56}, x_{77}$	1.000	n.a.
x_{10}	$R00245MM$	$x_{10}, x_{61}, x_{128}*$	1.000	1.127
x_{11}	$R00256MM$	$x_{11}*$	0.999	0.687
x_{12}	$R00258MM$	$x_{12}, x_{16}, x_{20}, x_{21}, x_{57}$	n.a.	0.687
x_{13}	$R00275MM$	$x_{13}, x_{65}*$	1.000	0.897
x_{14}	$R00330MM$	$x_{14}, x_{27}, x_{45}*$	1.000	8.066
x_{15}	$R00342MM$	$x_{15}, x_{134}**$	1.000	0.764
x_{16}	$R00351MM$	$x_{16}, x_{19}, x_{21}, x_{57}, x_{73}*$	1.000	0.941
x_{17}	$R00355MM$	$x_{17}, x_{19}*$	1.000	0.689
x_{18}	$R00371MM$	$x_9, x_{18}, x_{35}, x_{77}, x_{94}$	0.366	0.687
x_{19}	$R00388MM$	$x_{19}, x_{57}, x_{73}, x_{134}*$	1.000	0.706
x_{20}	$R00430MM$	$x_{20}, x_{86}, x_{105}, x_{113}*$	1.000	8.212
x_{21}	$R00432MM$	$x_{21}, x_{57}*$	1.000	0.941
x_{22}	$R00512MM$	$x_{22}, x_{54}*$	1.000	1.604
x_{23}	$R00551MM$	$x_8, x_{23}, x_{25}, x_{78}*$	0.999	0.687
x_{24}	$R00572MM$	$x_{20}, x_{24}*$	1.000	1.986
x_{25}	$R00667MM$	$x_{23}, x_{25}, x_{61}, x_{88}*$	1.000	0.687
x_{26}	$R00705MM$	$x_9, x_{26}, x_{30}, x_{55}, x_{56}, x_{77}$	1.000	n.a.
x_{27}	$R00709MM$	$x_{27}, x_{45}**$	1.000	0.941
x_{28}	$R00713MM$	$x_{28}, x_{50}**$	1.000	11.963
x_{29}	$R00716MM$	$x_9, x_{29}, x_{30}, x_{56}*$	1.000	0.687
x_{30}	$R00740MM$	$x_9, x_{26}, x_{30}, x_{55}, x_{56}, x_{77}$	1.000	n.a.
x_{31}	$R00830MM$	$x_{31}, x_{37}, x_{57}, x_{73}, x_{123}, x_{128}*$	0.998	17.980
x_{32}	$R00833MM$	$x_9, x_{30}, x_{32}, x_{55}*$	0.999	0.687
x_{33}	$R00851MM$	$x_{22}, x_{33}, x_{63}*$	1.000	1.604
x_{34}	$R00927MM$	$x_{34}*$	0.998	36.368
x_{35}	$R00941MM$	$x_{26}, x_{30}, x_{35}, x_{77}*$	0.999	0.687
x_{36}	$R00945MM$	$x_{30}, x_{36}*$	1.000	0.687
x_{37}	$R01082MM$	$x_{16}, x_{19}, x_{37}, x_{73}*$	1.000	0.983
x_{38}	$R01175MM$	$x_{38}, x_{43}, x_{87}, x_{117}, x_{118}*$	1.000	1.833
x_{39}	$R01177MM$	$x_{39}, x_{86}, x_{90}, x_{91}, x_{107}*$	1.000	8.340
x_{40}	$R01214MM$	$x_{30}, x_{40}, x_{55}*$	1.000	0.687
x_{41}	$R01218MM$	$x_{41}, x_{56}*$	1.000	1.453

Table H. Part I. Pathological stage in the succinate dehydrogenase deficiency. There are nine strong functional groups. Interestingly, the group (x_{28}, x_{50}) has been detected in all the three stages of the disease.

Variable	Flux	Flux groups	r^2	cv
x_{42}	$R01253MM$	$x_{26}, x_{42}, x_{55}, x_{77}$*	0.999	0.687
x_{43}	$R01279MM$	$x_{38}, x_{43}, x_{85}, x_{118}$*	1.000	1.833
x_{44}	$R01280MM$	$x_{44}, x_{53}, x_{103}, x_{124}$**	1.000	21.878
x_{45}	$R01325MM$	x_{27}, x_{45}**	1.000	0.941
x_{46}	$R01360MM$	x_{46}*	0.999	13.394
x_{47}	$R01361MM$	x_{47}*	0.998	13.394
x_{48}	$R01624MM$	x_{48}, x_{96}*	1.000	21.941
x_{49}	$R01626MM$	x_{49}, x_{96}*	1.000	21.897
x_{50}	$R01648MM$	x_{28}, x_{50}**	1.000	11.963
x_{51}	$R01655MM$	$x_{26}, x_{30}, x_{51}, x_{56}$*	0.999	0.687
x_{52}	$R01700MM$	x_{27}, x_{52}*	1.000	0.944
x_{53}	$R01706MM$	$x_{44}, x_{53}, x_{103}, x_{124}$**	1.000	21.878
x_{54}	$R01799MM$	x_{22}, x_{33}, x_{54}*	1.000	1.604
x_{55}	$R01801MM$	$x_9, x_{26}, x_{30}, x_{55}, x_{56}, x_{77}$	1.000	n.a.
x_{56}	$R01859MM$	$x_9, x_{26}, x_{30}, x_{55}, x_{56}, x_{77}$	1.000	n.a.
x_{57}	$R01900MM$	x_{16}, x_{57}*	1.000	0.941
x_{58}	$R01923MM$	x_{10}, x_{58}*	1.000	1.833
x_{59}	$R01939MM$	x_{59}, x_{70}*	1.000	0.687
x_{60}	$R01940MM$	$x_9, x_{30}, x_{60}, x_{77}$*	0.999	0.687
x_{61}	$R01975MM$	x_8, x_{61}*	1.000	14.259
x_{62}	$R01978MM$	x_{62}*	0.997	13.394
x_{63}	$R02030MM$	x_{33}, x_{54}, x_{63}*	1.000	1.604
x_{64}	$R02161MM$	x_3, x_4, x_{19}, x_{64}*	1.000	0.924
x_{65}	$R02163MM$	$x_4, x_{13}, x_{27}, x_{64}, x_{65}$*	1.000	0.897
x_{66}	$R02164MM$	x_{52}, x_{66}*	1.000	0.983
x_{67}	$R02199MM$	x_{67}*	0.999	33.271
x_{68}	$R02241MM$	x_{68}, x_{129}**	1.000	1.604
x_{69}	$R02313MM$	x_{26}, x_{69}*	1.000	0.687
x_{70}	$R02487MM$	$x_9, x_{26}, x_{52}, x_{55}, x_{70}, x_{77}$*	0.998	0.687
x_{71}	$R02529MM$	$x_{40}, x_{56}, x_{71}, x_{75}, x_{77}$*	1.000	0.687
x_{72}	$R02569MM$	$x_{19}, x_{27}, x_{45}, x_{52}, x_{72}$	-1.261	0.688
x_{73}	$R02570MM$	x_{21}, x_{73}*	1.000	0.944
x_{74}	$R02571MM$	x_{74}*	0.999	0.687
x_{75}	$R02661MM$	$x_{26}, x_{55}, x_{75}, x_{77}$*	0.999	0.687
x_{76}	$R02662MM$	x_{55}, x_{76}*	1.000	0.687
x_{77}	$R02765MM$	$x_9, x_{26}, x_{30}, x_{55}, x_{56}, x_{77}$	1.000	n.a.
x_{78}	$R03026MM$	$x_8, x_{61}, x_{78}, x_{107}$*	1.000	14.259
x_{79}	$R03102MM$	$x_{26}, x_{30}, x_{55}, x_{79}$*	0.999	0.687
x_{80}	$R03172MM$	x_{80}	-1.396	28.449
x_{81}	$R03174MM$	x_{81}*	0.998	35.829
x_{82}	$R03270MM$	x_2, x_{82}**	1.000	0.800
x_{83}	$R03314MM$	$x_9, x_{55}, x_{56}, x_{83}$*	1.000	0.687
x_{84}	$R03381MM$	$x_{30}, x_{55}, x_{77}, x_{84}$*	0.999	0.687
x_{85}	$R03777MM$	x_{85}, x_{117}*	1.000	1.833
x_{86}	$R03778MM$	$x_{39}, x_{86}, x_{88}, x_{91}, x_{109}$*	1.000	8.340
x_{87}	$R03857MM$	x_{43}, x_{87}, x_{117}*	1.000	1.833
x_{88}	$R03858MM$	$x_{39}, x_{88}, x_{90}, x_{107}, x_{111}$*	1.000	8.340

Table H. Part II. Pathological stage in the succinate dehydrogenase deficiency.

Variable	Flux	Flux groups	r^2	cv
x_{89}	$R03990MM$	x_{38}, x_{89}*	1.000	1.833
x_{90}	$R03991MM$	$x_{90}, x_{91}, x_{105}, x_{115}$*	1.000	8.340
x_{91}	$R04170MM$	$x_{39}, x_{91}, x_{109}, x_{111}, x_{114}$*	1.000	8.340
x_{92}	$R04203MM$	x_{92}*	1.000	37.606
x_{93}	$R04204MM$	x_{93}*	0.998	38.184
x_{94}	$R04224MM$	$x_{9}, x_{30}, x_{56}, x_{94}$*	0.999	0.687
x_{95}	$R04355MM$	x_{19}, x_{95}*	0.999	3.124
x_{96}	$R04428MM$	x_{96}, x_{119}**	1.000	21.945
x_{97}	$R04430MM$	$x_{97}, x_{100}, x_{120}, x_{122}, x_{123}$**	1.000	21.944
x_{98}	$R04433MM$	x_{15}, x_{98}*	1.000	1.646
x_{99}	$R04533MM$	$x_{97}, x_{99}, x_{120}, x_{122}, x_{123}$**	1.000	21.945
x_{100}	$R04536MM$	$x_{97}, x_{99}, x_{100}, x_{120}, x_{122}$*	1.000	21.942
x_{101}	$R04537MM$	$x_{48}, x_{96}, x_{101}, x_{119}, x_{121}$**	1.000	21.944
x_{102}	$R04543MM$	x_{102}, x_{125}**	1.000	21.877
x_{103}	$R04544MM$	x_{44}, x_{103}*	1.000	21.880
x_{104}	$R04737MM$	$x_{104}, x_{106}, x_{110}, x_{112}, x_{115}$*	1.000	8.340
x_{105}	$R04738MM$	x_{39}, x_{105}*	1.000	8.340
x_{106}	$R04739MM$	$x_{104}, x_{106}, x_{116}$*	1.000	8.340
x_{107}	$R04740MM$	x_{105}, x_{107}*	1.000	8.340
x_{108}	$R04741MM$	$x_{104}, x_{108}, x_{110}, x_{116}$*	1.000	8.340
x_{109}	$R04742MM$	$x_{86}, x_{90}, x_{105}, x_{109}, x_{116}$*	1.000	8.340
x_{110}	$R04743MM$	$x_{104}, x_{106}, x_{108}, x_{110}, x_{115}$*	1.000	8.340
x_{111}	$R04744MM$	$x_{91}, x_{107}, x_{111}, x_{114}, x_{116}$*	1.000	8.340
x_{112}	$R04745MM$	$x_{104}, x_{106}, x_{110}, x_{112}$*	1.000	8.340
x_{113}	$R04746MM$	$x_{88}, x_{107}, x_{109}, x_{111}, x_{113}$*	1.000	8.340
x_{114}	$R04747MM$	$x_{90}, x_{107}, x_{109}, x_{111}, x_{114}$*	1.000	8.340
x_{115}	$R04748MM$	$x_{104}, x_{108}, x_{110}, x_{112}, x_{115}$*	1.000	8.340
x_{116}	$R04749MM$	$x_{39}, x_{105}, x_{107}, x_{114}, x_{116}$*	1.000	8.340
x_{117}	$R04751MM$	x_{43}, x_{117}*	1.000	1.833
x_{118}	$R04754MM$	x_{87}, x_{117}, x_{118}*	1.000	1.833
x_{119}	$R04952MM$	x_{96}, x_{119}**	1.000	21.942
x_{120}	$R04953MM$	$x_{97}, x_{99}, x_{120}, x_{122}, x_{123}$**	1.000	21.942
x_{121}	$R04954MM$	$x_{48}, x_{96}, x_{101}, x_{119}, x_{121}$**	1.000	21.944
x_{122}	$R04956MM$	$x_{97}, x_{100}, x_{120}, x_{122}, x_{123}$**	1.000	21.944
x_{123}	$R04959MM$	$x_{99}, x_{100}, x_{120}, x_{122}, x_{123}$*	1.000	21.944
x_{124}	$R04968MM$	x_{103}, x_{124}*	1.000	21.881
x_{125}	$R04970MM$	x_{102}, x_{125}**	1.000	21.880
x_{126}	$R05064MM$	x_{30}, x_{126}*	1.000	0.687
x_{127}	$R05066MM$	$x_{9}, x_{30}, x_{77}, x_{127}$*	0.999	0.687
x_{128}	$R07162MM$	$x_{13}, x_{15}, x_{65}, x_{128}, x_{134}$*	1.000	0.706
x_{129}	$R07390MM$	x_{68}, x_{129}**	1.000	1.604
x_{130}	$R07599MM$	$x_{26}, x_{30}, x_{55}, x_{130}$*	0.999	0.687
x_{131}	$R07600MM$	x_{77}, x_{131}*	1.000	0.687
x_{132}	$R07603MM$	x_{132}*	0.999	33.521
x_{133}	$R07604MM$	x_{132}, x_{133}*	1.000	33.448
x_{134}	$R07618MM$	x_{15}, x_{134}**	1.000	0.758
x_{135}	$R08157MM$	x_{9}, x_{26}, x_{135}*	1.000	0.687

Table H. Part III. Pathological stage in the succinate dehydrogenase deficiency.

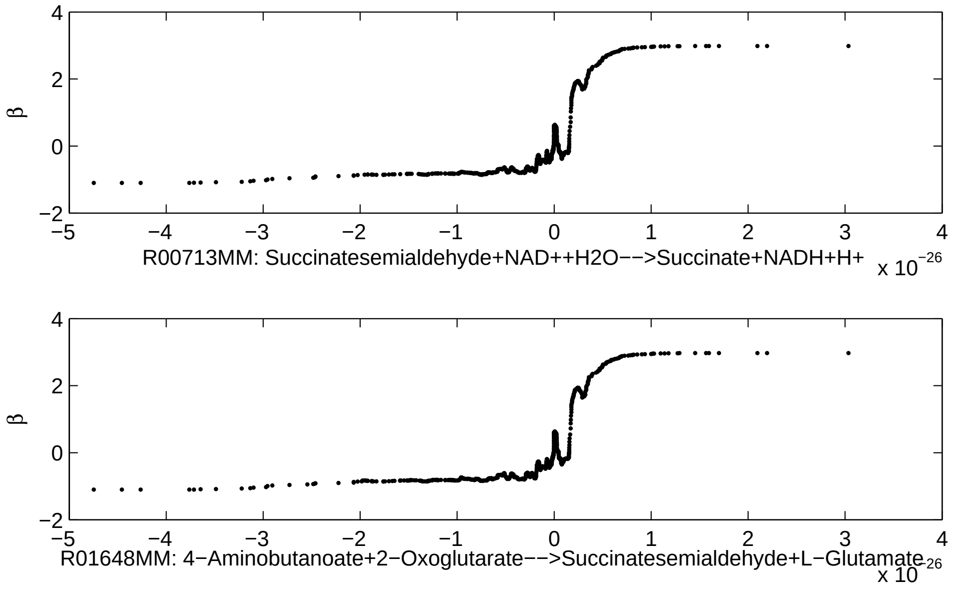

Figure S21. Succinate dehydrogenase deficiency - healthy stage. Optimal transformations β (y axis) found for the two fluxes R00713MM and R01648MM (x axis) [μmol min^{-1} gDW^{-1}] in the mitochondrial FBA model [2].

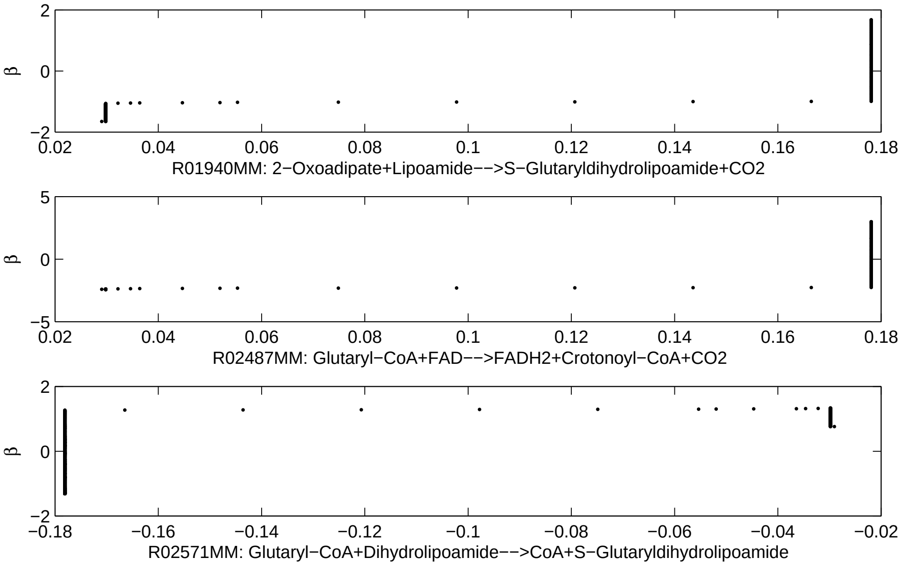

Figure S22. Succinate dehydrogenase deficiency - healthy stage. Optimal transformations β (y axis) found for the three fluxes R01940MM, R02487MM, and R02571MM (x axis) [μmol min^{-1} gDW^{-1}] in the mitochondrial FBA model [2].

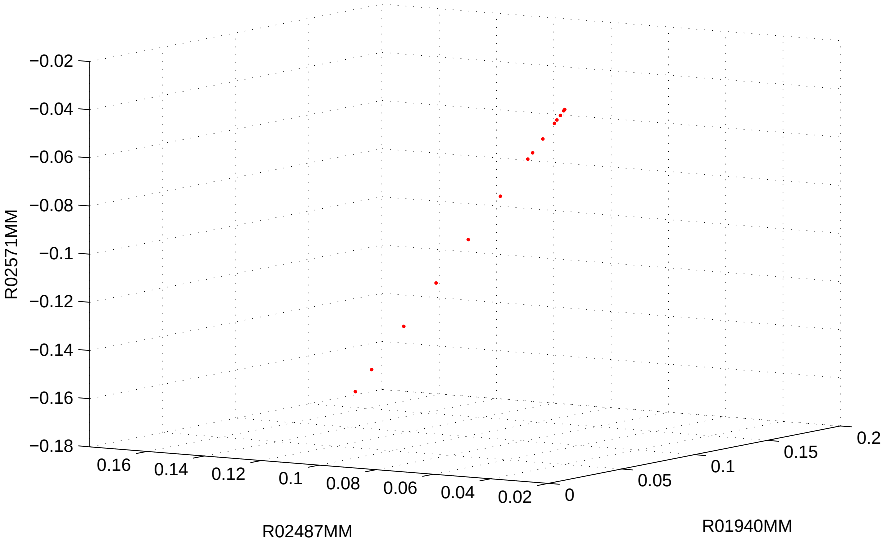

Figure S23. Succinate dehydrogenase deficiency - healthy stage. Functional relation β (y axis) found for the three fluxes R01940MM, R02487MM, and R02571MM (x axis) [μmol min^{-1} gDW^{-1}] in the mitochondrial FBA model [2].

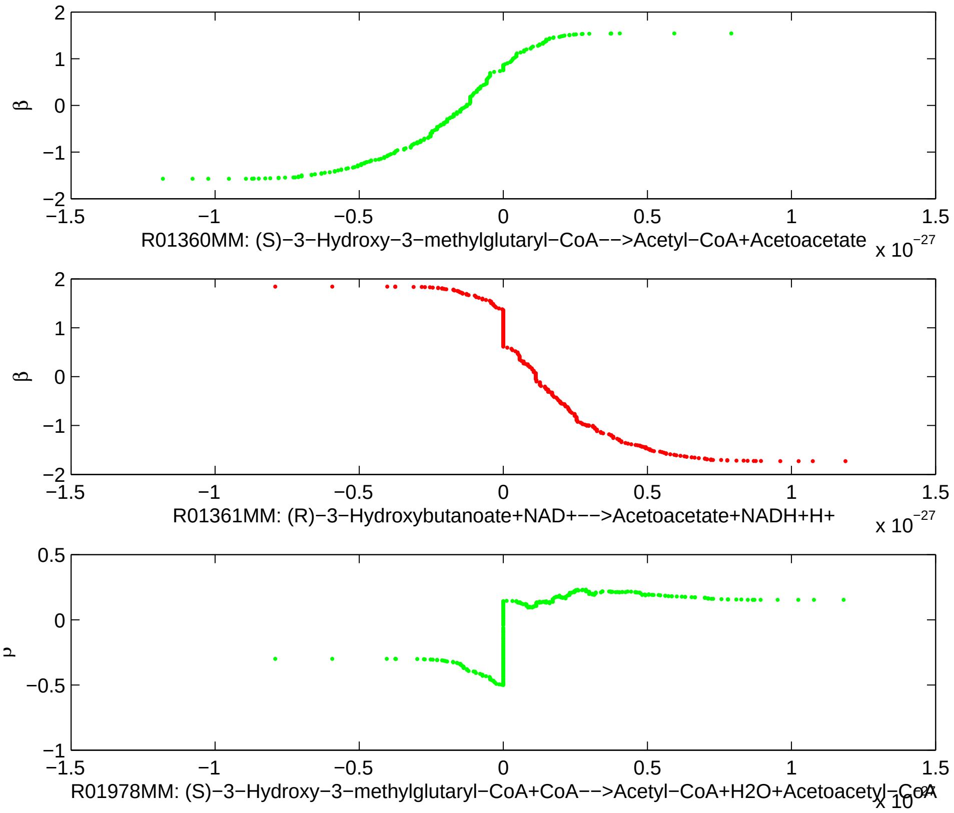

Figure S24. Succinate dehydrogenase deficiency - inflammation stage. Optimal transformations β (y axis) found for the three fluxes R01360MM, R01361MM, and R01978MM (x axis) [μmol min^{-1} gDW^{-1}] in the mitochondrial FBA model [2].

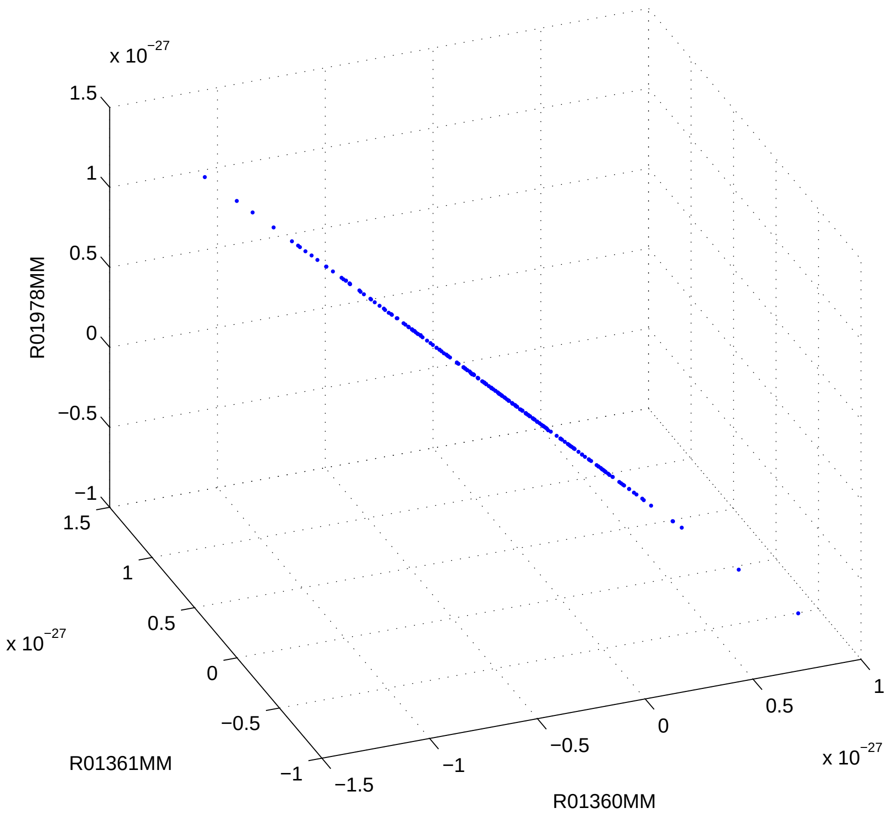

Figure S25. Succinate dehydrogenase deficiency - inflammation stage. Functional relation β (y axis) found for the three fluxes R01360MM, R01361MM, and R01978MM (x axis) [μmol min^{-1} gDW^{-1}] in the mitochondrial FBA model [2].

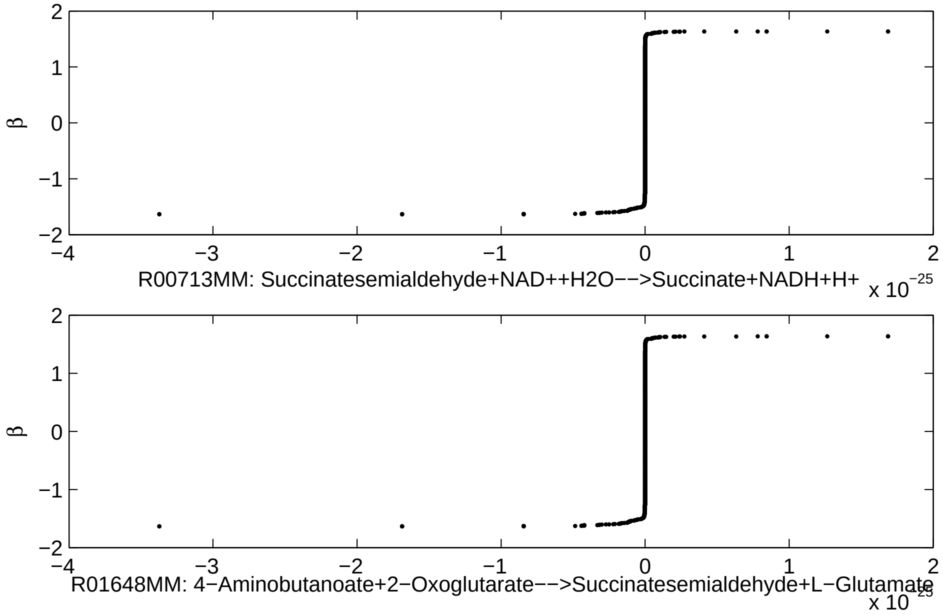

Figure S26. Succinate dehydrogenase deficiency - pathological stage. Optimal transformations β (y axis) found for the two fluxes R00713MM and R01648MM (x axis) [μmol min^{-1} gDW^{-1}] in the mitochondrial FBA model [2].

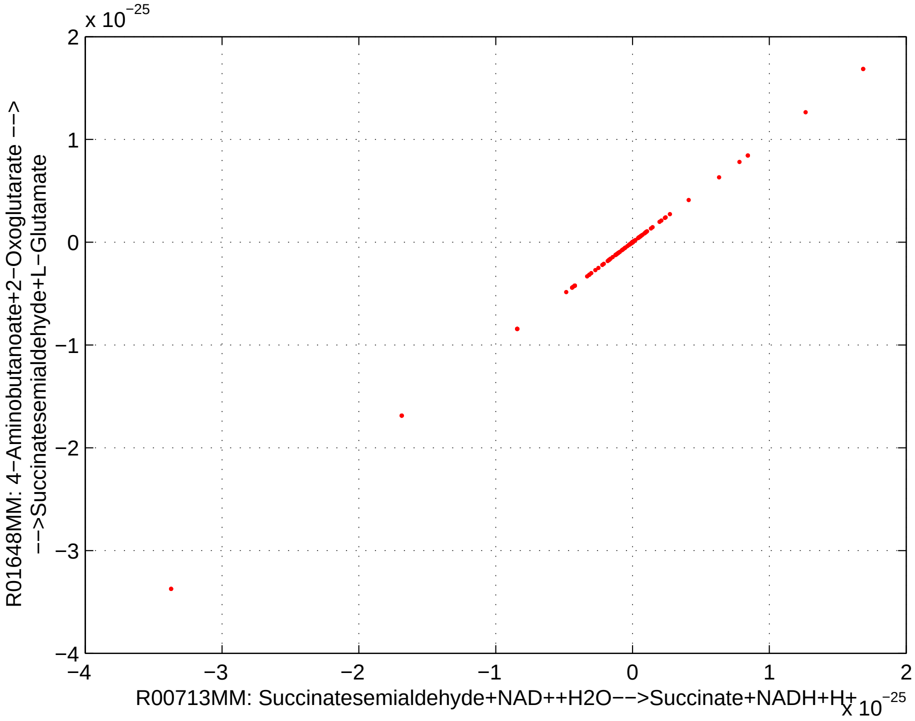

Figure S27. Succinate dehydrogenase deficiency - pathological stage. Functional relation β (y axis) found for the two fluxes R00713MM and R01648MM (x axis) [μmol min^{-1} gDW^{-1}] in the mitochondrial FBA model [2].

Variable	Flux	Flux groups	r^2	cv
x_1	$R00004MM$	x_1, x_5**	1.000	2.453
x_2	$R00014MM$	$x_2, x_{12}, x_{72}, x_{82}$	1.000	0.007
x_3	$R00081MM$	$x_3, x_6, x_{20}, x_{22}, x_{24}$	1.000	0.000
x_4	$R00086MM$	$x_4, x_{86}, x_{107}, x_{113}$	0.999	0.009
x_5	$R00127MM$	x_1, x_5**	1.000	2.453
x_6	$R00157MM$	$x_6, x_{20}, x_{22}, x_{24}, x_{26}, x_{30}$	1.000	n.a.
x_7	$R00205MM$	x_7, x_{18}, x_{71}**	1.000	2.953
x_8	$R00238MM$	$x_8, x_{12}, x_{78}, x_{82}$	1.000	0.059
x_9	$R00243MM$	$x_2, x_9, x_{11}, x_{12}, x_{72}, x_{82}$*	1.000	0.416
x_{10}	$R00245MM$	$x_{10}, x_{77}, x_{81}, x_{93}$*	1.000	0.655
x_{11}	$R00256MM$	$x_2, x_{11}, x_{12}, x_{61}, x_{72}, x_{82}$*	1.000	0.416
x_{12}	$R00258MM$	$x_2, x_{11}, x_{12}, x_{82}$*	1.000	1.827
x_{13}	$R00275MM$	x_8, x_{13}	0.999	0.016
x_{14}	$R00330MM$	$x_{11}, x_{12}, x_{14}, x_{21}, x_{78}$	1.000	0.038
x_{15}	$R00342MM$	x_{15}, x_{66}	1.000	0.031
x_{16}	$R00351MM$	x_{16}, x_{57}	1.000	0.034
x_{17}	$R00355MM$	x_{17}	1.000	0.026
x_{18}	$R00371MM$	x_7, x_{18}, x_{71}**	1.000	2.953
x_{19}	$R00388MM$	x_{19}, x_{61}	0.999	0.016
x_{20}	$R00430MM$	$x_6, x_{20}, x_{22}, x_{24}, x_{26}, x_{30}$	1.000	n.a.
x_{21}	$R00432MM$	$x_{11}, x_{12}, x_{14}, x_{21}, x_{61}, x_{78}$	1.000	0.038
x_{22}	$R00512MM$	$x_6, x_{20}, x_{22}, x_{24}, x_{26}, x_{30}$	1.000	n.a.
x_{23}	$R00551MM$	x_{23}, x_{68}	1.000	0.000
x_{24}	$R00572MM$	$x_6, x_{20}, x_{22}, x_{24}, x_{26}, x_{30}$	1.000	n.a.
x_{25}	$R00667MM$	x_{25}, x_{31}	1.000	0.000
x_{26}	$R00705MM$	$x_6, x_{20}, x_{22}, x_{24}, x_{26}, x_{30}$	1.000	n.a.
x_{27}	$R00709MM$	x_{27}, x_{45}	1.000	0.034
x_{28}	$R00713MM$	x_{28}, x_{50}**	1.000	n.a.
x_{29}	$R00716MM$	x_{25}, x_{26}, x_{29}*	1.000	0.409
x_{30}	$R00740MM$	$x_6, x_{20}, x_{22}, x_{24}, x_{26}, x_{30}$	1.000	n.a.
x_{31}	$R00830MM$	$x_6, x_{20}, x_{22}, x_{24}, x_{26}, x_{31}$	1.000	n.a.
x_{32}	$R00833MM$	x_{32}, x_{132}*	1.000	0.427
x_{33}	$R00851MM$	$x_6, x_{20}, x_{22}, x_{24}, x_{26}, x_{33}$	1.000	n.a.
x_{34}	$R00927MM$	$x_{20}, x_{30}, x_{34}, x_{46}, x_{54}$*	1.000	0.429
x_{35}	$R00941MM$	$x_{35}, x_{36}, x_{41}, x_{51}$**	1.000	0.424
x_{36}	$R00945MM$	$x_{35}, x_{36}, x_{41}, x_{51}$**	1.000	0.424
x_{37}	$R01082MM$	x_{37}, x_{66}	0.999	0.041
x_{38}	$R01175MM$	x_{38}, x_{115}	1.000	0.050
x_{39}	$R01177MM$	x_{39}, x_{114}	1.000	0.047
x_{40}	$R01214MM$	x_{40}, x_{75}, x_{131}*	1.000	0.427
x_{41}	$R01218MM$	$x_{35}, x_{36}, x_{41}, x_{51}$**	1.000	0.424

Table I. Part I. Healthy stage in the α-ketoglutarate dehydrogenase deficiency. The algorithm has detected five functional relations among the fluxes.

Name	Formula
R00004MM	Pyrophosphate+H2O ⟶ 2Orthophosphate
R00014MM	Pyruvate+Thiam ⟶ 2-(alpha-Hydroxyethyl)thiaminediphosphate+CO2
R00081MM	Oxygen+4Ferrocytochromec ⟶ 4Ferricytochromec+2H2O
R00086MM	ADP+Orthophosphate+4H+ ⟶ ATP+H2O
R00127MM	ATP+AMP ⟶ 2ADP
R00157MM	UTP+AMP ⟶ UDP+ADP
R00205MM	Methylglyoxal+NADP++H2O ⟶ Pyruvate+NADPH+H+
R00238MM	2Acetyl-CoA ⟶ CoA+Acetoacetyl-CoA
R00243MM	L-Glutamate+NAD++H2O ⟶ 2-Oxoglutarate+NH3+NADH+H+
R00245MM	L-Glutamate5-semialdehyde+NAD++H2O ⟶ L-Glutamate+NADH+H+
R00256MM	L-Glutamine+H2O ⟶ L-Glutamate+NH3
R00258MM	L-Alanine+2-Oxoglutarate ⟶ Pyruvate+L-Glutamate
R00275MM	2O2.-+2H+ ⟶ H2O2+Oxygen
R00330MM	ATP+GDP ⟶ ADP+GTP
R00342MM	(S)-Malate+NAD+ ⟶ Oxaloacetate+NADH+H+
R00351MM	Citrate+CoA ⟶ Acetyl-CoA+H2O+Oxaloacetate
R00355MM	L-Aspartate+2-Oxoglutarate ⟶ Oxaloacetate+L-Glutamate
R00371MM	Acetyl-CoA+Glycine ⟶ CoA+L-2-Amino-3-oxobutanoicacid
R00388MM	Acyl-CoA+Oxygen ⟶ trans-2,3-Dehydroacyl-CoA+H2O2
R00430MM	GTP+Pyruvate ⟶ GDP+Phosphoenolpyruvate
R00432MM	GTP+Succinate+CoA ⟶ GDP+Orthophosphate+Succinyl-CoA
R00512MM	ATP+CMP ⟶ ADP+CDP
R00551MM	L-Arginine+H2O ⟶ L-Ornithine+Urea
R00572MM	CTP+Pyruvate ⟶ CDP+Phosphoenolpyruvate
R00667MM	L-Ornithine+2-Oxoglutarate ⟶ L-Glutamate5-semialdehyde+L-Glutamate
R00705MM	3-Oxopropanoate+CoA+NAD+ ⟶ Acetyl-CoA+CO2+NADH+H+
R00709MM	Isocitrate+NAD+ ⟶ 2-Oxoglutarate+CO2+NADH+H+
R00713MM	Succinatesemialdehyde+NAD++H2O ⟶ Succinate+NADH+H+
R00716MM	N6-(L-1,3-Dicarboxypropyl)-L-lysine+NADP++H2O ⟶ L-Lysine+2-Oxoglutarate+NADPH+H+
R00740MM	3-Oxopropanoate+CoA+NADP+ ⟶ Malonyl-CoA+NADPH+H+
R00830MM	Succinyl-CoA+Glycine ⟶ 5-Aminolevulinate+CoA+CO2
R00833MM	(R)-2-Methyl-3-oxopropanoyl-CoA ⟶ Succinyl-CoA
R00851MM	sn-Glycerol3-phosphate+Palmitoyl-CoA ⟶ 1-Acyl-sn-glycerol3-phosphate+CoA
R00927MM	Propanoyl-CoA+Acetyl-CoA ⟶ CoA+2-Methylacetoacetyl-CoA

R00941MM	10-Formyltetrahydrofolate+NADP++H2O ⟶ Tetrahydrofolate+CO2+NADPH+H+
R00945MM	5,10-Methylenetetrahydrofolate+Glycine+H2O ⟶ Tetrahydrofolate+L-Serine
R01082MM	(S)-Malate ⟶ Fumarate+H2O
R01175MM	Butanoyl-CoA+FAD ⟶ FADH2+Crotonoyl-CoA
R01177MM	Acetyl-CoA+Butanoyl-CoA ⟶ CoA+3-Oxohexanoyl-CoA
R01214MM	L-Valine+2-Oxoglutarate ⟶ 3-Methyl-2-oxobutanoicacid+L-Glutamate
R01218MM	5,10-Methylenetetrahydrofolate+NAD+ ⟶ 5,10-Methenyltetrahydrofolate+NADH+H+
R01253MM	L-Proline+FAD ⟶ (S)-1-Pyrroline-5-carboxylate+FADH2
R01279MM	Palmitoyl-CoA+FAD ⟶ trans-Hexadec-2-enoyl-CoA+FADH2
R01280MM	ATP+Hexadecanoicacid+CoA ⟶ AMP+Palmitoyl-CoA+Pyrophosphate
R01325MM	Citrate ⟶ cis-Aconitate+H2O
R01360MM	(S)-3-Hydroxy-3-methylglutaryl-CoA ⟶ Acetyl-CoA+Acetoacetate
R01361MM	(R)-3-Hydroxybutanoate+NAD+ ⟶ Acetoacetate+NADH+H+
R01624MM	Acetyl-CoA+Acyl-carrierprotein ⟶ CoA+Acetyl-[acyl-carrierprotein]
R01626MM	Malonyl-CoA+Acyl-carrierprotein ⟶ CoA+Malonyl-[acyl-carrierprotein]
R01648MM	4-Aminobutanoate+2-Oxoglutarate ⟶ Succinatesemialdehyde+L-Glutamate
R01655MM	5,10-Methenyltetrahydrofolate+H2O ⟶ 10-Formyltetrahydrofolate+H+
R01700MM	2-Oxoglutarate+EnzymeN6-(lipoyl)lysine ⟶ [Dhlps-residuesuccinyltransferase]S-succinyldihydrolipoyllysine+CO2
R01706MM	Hexadecanoyl-[acp]+H2O ⟶ Acyl-carrierprotein+Hexadecanoicacid
R01799MM	CTP+Phosphatidate ⟶ Pyrophosphate+CDP-diacylglycerol
R01801MM	CDP-diacylglycerol+sn-Glycerol3-phosphate ⟶ CMP+Phosphatidylglycerophosphate
R01859MM	ATP+Propanoyl-CoA+HCO3- ⟶ ADP+Orthophosphate+(S)-2-Methyl-3-oxopropanoyl-CoA
R01900MM	Isocitrate ⟶ cis-Aconitate+H2O
R01923MM	Palmitoyl-CoA+L-Carnitine ⟶ CoA+L-Palmitoylcarnitine
R01939MM	L-2-Aminoadipate+2-Oxoglutarate ⟶ 2-Oxoadipate+L-Glutamate
R01940MM	2-Oxoadipate+Lipoamide ⟶ S-Glutaryldihydrolipoamide+CO2
R01975MM	(S)-3-Hydroxybutanoyl-CoA+NAD+ ⟶ Acetoacetyl-CoA+NADH+H+
R01978MM	(S)-3-Hydroxy-3-methylglutaryl-CoA+CoA ⟶ Acetyl-CoA+H2O+Acetoacetyl-CoA
R02030MM	Phosphatidylglycerol+CDP-diacylglycerol ⟶ Cardiolipin+CMP
R02161MM	Ubiquinol+2Ferricytochromec ⟶ Ubiquinone+2Ferrocytochromec
R02163MM	Ubiquinone+NADH ⟶ Ubiquinol+NAD+4H+0.002O2.-
R02164MM	Ubiquinone+Succinate ⟶ Ubiquinol+Fumarate
R02199MM	L-Isoleucine+2-Oxoglutarate ⟶ (S)-3-Methyl-2-oxopentanoicacid+L-Glutamate
R02241MM	Phosphatidate+CoA ⟶ 1-Acyl-sn-glycerol3-phosphate+Palmitoyl-CoA
R02313MM	N6-(L-1,3-Dicarboxypropyl)-L-lysine+NAD++H2O ⟶ L-Glutamate+L-2-Aminoadipate6-semialdehyde+NADH+H+
R02487MM	Glutaryl-CoA+FAD ⟶ FADH2+Crotonoyl-CoA+CO2
R02529MM	Aminoacetone+H2O+Oxygen ⟶ Methylglyoxal+NH3+H2O2
R02569MM	Acetyl-CoA+EnzymeN6-(dihydrolipoyl)lysine ⟶ CoA+[Dhlps-residueacetyltransferase]S-acetyldihydrolipoyllysine
R02570MM	Succinyl-CoA+EnzymeN6-(dihydrolipoyl)lysine ⟶ CoA+[Dhlps-residuesuccinyltransferase]S-succinyldihydrolipoyllysine
R02571MM	Glutaryl-CoA+Dihydrolipoamide ⟶ CoA+S-Glutaryldihydrolipoamide
R02661MM	2-Methylpropanoyl-CoA+FAD ⟶ 2-Methylprop-2-enoyl-CoA+FADH2
R02662MM	2-Methylpropanoyl-CoA+EnzymeN6-(dihydrolipoyl)lysine ⟶ CoA+[Dhlps-residue(2-methylpropanoyl)transferase]S-(2-methylpropanoyl)Dhlps
R02765MM	(R)-Methylmalonyl-CoA ⟶ (S)-Methylmalonyl-CoA
R03026MM	(S)-3-Hydroxybutanoyl-CoA ⟶ Crotonoyl-CoA+H2O
R03102MM	L-2-Aminoadipate6-semialdehyde+NAD++H2O ⟶ L-2-Aminoadipate+NADH+H+
R03172MM	(S)-2-Methylbutanoyl-CoA+FAD ⟶ 2-Methylbut-2-enoyl-CoA+FADH2
R03174MM	(S)-2-Methylbutanoyl-CoA+EnzymeN6-(dihydrolipoyl)lysine ⟶ CoA+[Dhlps-residue(2-methylpropanoyl)transferase]S-(2-methylbutanoyl)Dhlps
R03270MM	2-(alpha-Hydroxyethyl)thiaminediphosphate+EnzymeN6-(lipoyl)lysine ⟶ [Dhlps-residueacetyltransferase]S-acetyldihydrolipoyllysine+Thiam
R03314MM	L-Glutamate5-semialdehyde ⟶ (S)-1-Pyrroline-5-carboxylate+H2O
R03381MM	(S)-Methylmalonatesemialdehyde+CoA+NAD+ ⟶ (R)-2-Methyl-3-oxopropanoyl-CoA+NADH+H+
R03777MM	Octanoyl-CoA+FAD ⟶ trans-Oct-2-enoyl-CoA+FADH2
R03778MM	Octanoyl-CoA+Acetyl-CoA ⟶ CoA+3-Oxodecanoyl-CoA
R03857MM	Lauroyl-CoA+FAD ⟶ 2-trans-Dodecenoyl-CoA+FADH2
R03858MM	Lauroyl-CoA+Acetyl-CoA ⟶ CoA+3-Oxotetradecanoyl-CoA
R03990MM	Tetradecanoyl-CoA+FAD ⟶ trans-Tetradec-2-enoyl-CoA+FADH2
R03991MM	Tetradecanoyl-CoA+Acetyl-CoA ⟶ CoA+3-Oxopalmitoyl-CoA
R04170MM	(S)-3-Hydroxydodecanoyl-CoA ⟶ 2-trans-Dodecenoyl-CoA+H2O
R04203MM	(2S,3S)-3-Hydroxy-2-methylbutanoyl-CoA+NAD+ ⟶ 2-Methylacetoacetyl-CoA+NADH+H+
R04204MM	(2S,3S)-3-Hydroxy-2-methylbutanoyl-CoA ⟶ 2-Methylbut-2-enoyl-CoA+H2O
R04224MM	2-Methylprop-2-enoyl-CoA+H2O ⟶ (S)-3-Hydroxyisobutyryl-CoA
R04355MM	Acetyl-[acyl-carrierprotein]+Malonyl-[acyl-carrierprotein] ⟶ Acetoacetyl-[acp]+CO2+Acyl-carrierprotein
R04428MM	(3R)-3-Hydroxybutanoyl-[acyl-carrierprotein] ⟶ But-2-enoyl-[acyl-carrierprotein]+H2O
R04430MM	Butyryl-[acp]+NADP+ ⟶ But-2-enoyl-[acyl-carrierprotein]+NADPH+H+
R04433MM	Ubiquinone+FADH2 ⟶ Ubiquinol+FAD+
R04533MM	(3R)-3-Hydroxybutanoyl-[acyl-carrierprotein]+NADP+ ⟶ Acetoacetyl-[acp]+NADPH+H+
R04536MM	(3R)-3-Hydroxyoctanoyl-[acyl-carrierprotein]+NADP+ ⟶ 3-Oxooctanoyl-[acp]+NADPH+H+
R04537MM	(3R)-3-Hydroxyoctanoyl-[acyl-carrierprotein] ⟶ trans-Oct-2-enoyl-[acp]+H2O
R04543MM	(3R)-3-Hydroxypalmitoyl-[acyl-carrierprotein]+NADP+ ⟶ 3-Oxohexadecanoyl-[acp]+NADPH+H+
R04544MM	(3R)-3-Hydroxypalmitoyl-[acyl-carrierprotein] ⟶ trans-Hexadec-2-enoyl-[acp]+H2O
R04737MM	(S)-3-Hydroxyhexadecanoyl-CoA+NAD+ ⟶ 3-Oxopalmitoyl-CoA+NADH+H+
R04738MM	(S)-3-Hydroxyhexadecanoyl-CoA ⟶ trans-Hexadec-2-enoyl-CoA+H2O
R04739MM	(S)-3-Hydroxytetradecanoyl-CoA+NAD+ ⟶ 3-Oxotetradecanoyl-CoA+NADH
R04740MM	(S)-3-Hydroxytetradecanoyl-CoA ⟶ trans-Tetradec-2-enoyl-CoA+H2O
R04741MM	(S)-3-Hydroxydodecanoyl-CoA+NAD+ ⟶ 3-Oxododecanoyl-CoA+NADH+H+
R04742MM	Decanoyl-CoA+Acetyl-CoA ⟶ CoA+3-Oxododecanoyl-CoA
R04743MM	(S)-Hydroxydecanoyl-CoA+NAD+ ⟶ 3-Oxodecanoyl-CoA+NADH+H+
R04744MM	(S)-Hydroxydecanoyl-CoA ⟶ trans-Dec-2-enoyl-CoA+H2O
R04745MM	(S)-Hydroxyoctanoyl-CoA+NAD+ ⟶ 3-Oxooctanoyl-CoA+NADH+H+
R04746MM	(S)-Hydroxyoctanoyl-CoA ⟶ trans-Oct-2-enoyl-CoA+H2O
R04747MM	Hexanoyl-CoA+Acetyl-CoA ⟶ CoA+3-Oxooctanoyl-CoA
R04748MM	(S)-Hydroxyhexanoyl-CoA+NAD+ ⟶ 3-Oxohexanoyl-CoA+NADH+H+
R04749MM	(S)-Hydroxyhexanoyl-CoA ⟶ trans-Hex-2-enoyl-CoA+H2O
R04751MM	Hexanoyl-CoA+FAD ⟶ trans-Hex-2-enoyl-CoA+FADH2
R04754MM	Decanoyl-CoA+FAD ⟶ trans-Dec-2-enoyl-CoA+FADH2
R04952MM	Butyryl-[acp]+Malonyl-[acyl-carrierprotein] ⟶ 3-Oxohexanoyl-[acp]+CO2+Acyl-carrierprotein
R04953MM	(R)-3-Hydroxyhexanoyl-[acp]+NADP+ ⟶ 3-Oxohexanoyl-[acp]+NADPH+H+
R04954MM	(R)-3-Hydroxyhexanoyl-[acp] ⟶ trans-Hex-2-enoyl-[acp]+H2O
R04956MM	Hexanoyl-[acp]+NADP+ ⟶ trans-Hex-2-enoyl-[acp]+NADPH+H+
R04959MM	Octanoyl-[acp]+NADP+ ⟶ trans-Oct-2-enoyl-[acp]+NADPH+H+
R04968MM	Tetradecanoyl-[acp]+Malonyl-[acyl-carrierprotein] ⟶ 3-Oxohexadecanoyl-[acp]+CO2+Acyl-carrierprotein
R04970MM	Hexadecanoyl-[acp]+NADP+ ⟶ trans-Hexadec-2-enoyl-[acp]+NADPH+H+
R05064MM	(S)-3-Hydroxyisobutyryl-CoA+H2O ⟶ CoA+(S)-3-Hydroxyisobutyrate
R05066MM	(S)-3-Hydroxyisobutyrate+NAD+ ⟶ (S)-Methylmalonatesemialdehyde+NADH+H+
R07162MM	Acyl-CoA+NADP+ ⟶ trans-2,3-Dehydroacyl-CoA+NADPH+H+
R07390MM	2Phosphatidylglycerol ⟶ Cardiolipin+Glycerol
R07599MM	3-Methyl-2-oxobutanoicacid+Thiam ⟶ 2-Methyl-1-hydroxypropyl-ThPP+CO2
R07600MM	2-Methyl-1-hydroxypropyl-ThPP+EnzymeN6-(lipoyl)lysine ⟶ [Dhlps-residue(2-methylpropanoyl)transferase]S-(2-methylpropanoyl)Dhlps+Thiam
R07603MM	(S)-3-Methyl-2-oxopentanoicacid+Thiam ⟶ 2-Methyl-1-hydroxybutyl-ThPP+CO2
R07604MM	2-Methyl-1-hydroxybutyl-ThPP+EnzymeN6-(lipoyl)lysine ⟶ [Dhlps-residue(2-methylpropanoyl)transferase]S-(2-methylbutanoyl)Dhlps+Thiam
R07618MM	EnzymeN6-(dihydrolipoyl)lysine+NAD+ ⟶ EnzymeN6-(lipoyl)lysine+NADH+H+
R08157MM	Octanoyl-[acp]+H2O ⟶ Acyl-carrierprotein+Octanoicacid

Table L. Reaction stoichiometry for the 135 reactions in the mitochondrial matrix considered for the IA. Abbreviations: Dhlps = Dihydrolipoyllysine, Thiam = Thiamindiphosphate.

Variable	Flux	Flux groups	r^2	cv
x_{42}	$R01253MM$	$x_6, x_{20}, x_{22}, x_{24}, x_{26}, x_{42}$	1.000	0.000
x_{43}	$R01279MM$	$x_{11}, x_{43}, x_{85}, x_{89}, x_{90}, x_{118}$	1.000	0.047
x_{44}	$R01280MM$	$x_{44}, x_{53}, x_{103}, x_{124}*$	1.000	4.912
x_{45}	$R01325MM$	x_{27}, x_{45}	1.000	0.034
x_{46}	$R01360MM$	$x_6, x_{20}, x_{22}, x_{24}, x_{26}, x_{46}$	1.000	0.000
x_{47}	$R01361MM$	$x_6, x_{20}, x_{22}, x_{24}, x_{26}, x_{47}$	1.000	0.000
x_{48}	$R01624MM$	$x_{48}, x_{96}, x_{101}, x_{103}, x_{124}*$	1.000	0.210
x_{49}	$R01626MM$	$x_{44}, x_{49}, x_{101}, x_{121}, x_{124}*$	1.000	0.520
x_{50}	$R01648MM$	$x_{28}, x_{50}**$	1.000	n.a.
x_{51}	$R01655MM$	$x_{41}, x_{51}*$	1.000	0.424
x_{52}	$R01700MM$	$x_8, x_{11}, x_{14}, x_{52}, x_{61}, x_{78}*$	1.000	0.578
x_{53}	$R01706MM$	$x_{44}, x_{53}, x_{96}, x_{101}, x_{121}*$	0.999	4.911
x_{54}	$R01799MM$	$x_6, x_{20}, x_{22}, x_{24}, x_{26}, x_{54}$	1.000	n.a.
x_{55}	$R01801MM$	$x_6, x_{20}, x_{22}, x_{24}, x_{26}, x_{55}$	1.000	n.a.
x_{56}	$R01859MM$	$x_6, x_{22}, x_{42}, x_{56}, x_{63}*$	0.998	0.429
x_{57}	$R01900MM$	x_{16}, x_{57}	1.000	0.034
x_{58}	$R01923MM$	$x_{58}, x_{88}, x_{90}, x_{105}, x_{109}$	1.000	0.047
x_{59}	$R01939MM$	$x_{59}, x_{69}*$	1.000	0.409
x_{60}	$R01940MM$	$x_{60}*$	1.000	0.962
x_{61}	$R01975MM$	$x_8, x_{11}, x_{12}, x_{61}, x_{72}, x_{78}*$	1.000	0.136
x_{62}	$R01978MM$	$x_{33}, x_{42}, x_{55}, x_{62}, x_{129}$	1.000	0.008
x_{63}	$R02030MM$	$x_6, x_{20}, x_{22}, x_{24}, x_{26}, x_{63}$	1.000	n.a.
x_{64}	$R02161MM$	$x_6, x_{20}, x_{22}, x_{24}, x_{64}$	-1.145	0.000
x_{65}	$R02163MM$	x_{61}, x_{65}	0.999	0.016
x_{66}	$R02164MM$	x_{15}, x_{37}, x_{66}	1.000	0.041
x_{67}	$R02199MM$	$x_{22}, x_{26}, x_{31}, x_{67}, x_{83}*$	0.998	0.429
x_{68}	$R02241MM$	$x_6, x_{20}, x_{22}, x_{24}, x_{26}, x_{68}$	1.000	n.a.
x_{69}	$R02313MM$	$x_{69}, x_{79}**$	1.000	0.409
x_{70}	$R02487MM$	$x_{70}*$	0.982	0.962
x_{71}	$R02529MM$	$x_7, x_{18}, x_{71}**$	1.000	2.953
x_{72}	$R02569MM$	x_2, x_{72}	1.000	0.007
x_{73}	$R02570MM$	x_8, x_9, x_{73}, x_{78}	1.000	0.039
x_{74}	$R02571MM$	$x_{74}*$	0.999	0.962
x_{75}	$R02661MM$	$x_{75}, x_{126}*$	1.000	0.427
x_{76}	$R02662MM$	$x_{46}, x_{54}, x_{76}, x_{83}, x_{129}*$	1.000	0.427
x_{77}	$R02765MM$	$x_{42}, x_{47}, x_{68}, x_{77}, x_{135}*$	1.000	0.429
x_{78}	$R03026MM$	$x_8, x_9, x_{61}, x_{78}*$	1.000	0.136
x_{79}	$R03102MM$	$x_{69}, x_{79}**$	1.000	0.409
x_{80}	$R03172MM$	$x_{24}, x_{80}*$	1.000	0.429
x_{81}	$R03174MM$	$x_{31}, x_{81}*$	1.000	0.429
x_{82}	$R03270MM$	$x_2, x_{11}, x_{12}, x_{72}, x_{78}, x_{82}$	1.000	0.007
x_{83}	$R03314MM$	$x_6, x_{20}, x_{22}, x_{24}, x_{26}, x_{83}$	1.000	0.000
x_{84}	$R03381MM$	$x_{75}, x_{84}, x_{126}, x_{127}*$	1.000	0.427
x_{85}	$R03777MM$	$x_{58}, x_{85}, x_{87}, x_{89}, x_{90}$	1.000	0.047
x_{86}	$R03778MM$	$x_{86}, x_{89}, x_{109}, x_{113}, x_{114}$	1.000	0.047
x_{87}	$R03857MM$	$x_{87}, x_{89}, x_{114}, x_{118}$	1.000	0.047
x_{88}	$R03858MM$	$x_{11}, x_{58}, x_{88}, x_{90}, x_{105}, x_{109}$	1.000	0.047

Table I. Part II. Healthy stage in the α-ketoglutarate dehydrogenase deficiency.

Variable	Flux	Flux groups	r^2	cv
x_{89}	$R03990MM$	$x_{58}, x_{85}, x_{89}, x_{104}, x_{114}$	1.000	0.047
x_{90}	$R03991MM$	$x_{12}, x_{39}, x_{86}, x_{88}, x_{90}, x_{105}$	1.000	0.047
x_{91}	$R04170MM$	$x_{9}, x_{39}, x_{58}, x_{90}, x_{91}, x_{114}$	1.000	0.047
x_{92}	$R04203MM$	$x_{26}, x_{30}, x_{54}, x_{68}, x_{92}*$	0.998	0.429
x_{93}	$R04204MM$	$x_{47}, x_{93}*$	1.000	0.429
x_{94}	$R04224MM$	$x_{94}, x_{126}*$	1.000	0.427
x_{95}	$R04355MM$	$x_{95}*$	0.977	0.210
x_{96}	$R04428MM$	$x_{48}, x_{96}, x_{102}, x_{121}, x_{124}*$	1.000	0.210
x_{97}	$R04430MM$	$x_{48}, x_{97}, x_{99}, x_{102}, x_{124}*$	1.000	0.210
x_{98}	$R04433MM$	$x_{43}, x_{90}, x_{98}, x_{112}, x_{114}$	1.000	0.058
x_{99}	$R04533MM$	$x_{44}, x_{97}, x_{99}, x_{102}, x_{125}*$	1.000	0.210
x_{100}	$R04536MM$	$x_{48}, x_{100}, x_{102}, x_{120}, x_{125}**$	1.000	0.210
x_{101}	$R04537MM$	$x_{48}, x_{96}, x_{101}, x_{102}, x_{124}*$	1.000	0.210
x_{102}	$R04543MM$	$x_{97}, x_{102}, x_{103}, x_{120}, x_{125}*$	1.000	4.912
x_{103}	$R04544MM$	$x_{44}, x_{100}, x_{103}, x_{124}, x_{125}*$	1.000	4.912
x_{104}	$R04737MM$	$x_{11}, x_{43}, x_{85}, x_{87}, x_{89}, x_{104}$	1.000	0.047
x_{105}	$R04738MM$	$x_{9}, x_{39}, x_{58}, x_{90}, x_{105}, x_{114}$	1.000	0.047
x_{106}	$R04739MM$	$x_{9}, x_{87}, x_{89}, x_{105}, x_{106}, x_{108}$	1.000	0.047
x_{107}	$R04740MM$	x_{107}, x_{109}	1.000	0.047
x_{108}	$R04741MM$	$x_{11}, x_{89}, x_{90}, x_{104}, x_{108}, x_{118}$	1.000	0.047
x_{109}	$R04742MM$	$x_{12}, x_{39}, x_{58}, x_{86}, x_{88}, x_{109}$	1.000	0.047
x_{110}	$R04743MM$	$x_{39}, x_{72}, x_{87}, x_{89}, x_{106}, x_{110}$	1.000	0.047
x_{111}	$R04744MM$	$x_{12}, x_{58}, x_{86}, x_{91}, x_{105}, x_{111}$	1.000	0.047
x_{112}	$R04745MM$	$x_{11}, x_{87}, x_{89}, x_{109}, x_{112}, x_{118}$	1.000	0.047
x_{113}	$R04746MM$	$x_{9}, x_{58}, x_{86}, x_{88}, x_{105}, x_{113}$	1.000	0.047
x_{114}	$R04747MM$	$x_{86}, x_{88}, x_{90}, x_{109}, x_{114}$	1.000	0.047
x_{115}	$R04748MM$	x_{115}, x_{118}	1.000	0.047
x_{116}	$R04749MM$	$x_{2}, x_{58}, x_{88}, x_{90}, x_{113}, x_{116}$	1.000	0.047
x_{117}	$R04751MM$	x_{89}, x_{117}	1.000	0.047
x_{118}	$R04754MM$	$x_{11}, x_{87}, x_{89}, x_{109}, x_{117}, x_{118}$	1.000	0.047
x_{119}	$R04952MM$	$x_{1}, x_{119}*$	0.999	0.210
x_{120}	$R04953MM$	$x_{48}, x_{100}, x_{102}, x_{120}, x_{125}**$	1.000	0.210
x_{121}	$R04954MM$	$x_{101}, x_{103}, x_{121}, x_{124}, x_{125}*$	1.000	0.210
x_{122}	$R04956MM$	$x_{48}, x_{102}, x_{120}, x_{122}, x_{124}*$	1.000	0.210
x_{123}	$R04959MM$	$x_{48}, x_{100}, x_{102}, x_{123}, x_{124}*$	1.000	0.210
x_{124}	$R04968MM$	$x_{44}, x_{48}, x_{96}, x_{101}, x_{124}*$	1.000	4.912
x_{125}	$R04970MM$	$x_{102}, x_{103}, x_{123}, x_{124}, x_{125}*$	1.000	4.912
x_{126}	$R05064MM$	$x_{84}, x_{126}*$	1.000	0.427
x_{127}	$R05066MM$	$x_{40}, x_{126}, x_{127}, x_{130}, x_{131}*$	1.000	0.427
x_{128}	$R07162MM$	x_{78}, x_{128}	0.999	0.016
x_{129}	$R07390MM$	$x_{6}, x_{20}, x_{22}, x_{24}, x_{26}, x_{129}$	1.000	n.a.
x_{130}	$R07599MM$	$x_{33}, x_{130}*$	1.000	0.427
x_{131}	$R07600MM$	$x_{94}, x_{131}*$	1.000	0.427
x_{132}	$R07603MM$	$x_{6}, x_{132}*$	1.000	0.429
x_{133}	$R07604MM$	$x_{31}, x_{133}*$	1.000	0.429
x_{134}	$R07618MM$	x_{116}, x_{134}	0.999	0.033
x_{135}	$R08157MM$	$x_{6}, x_{20}, x_{22}, x_{24}, x_{26}, x_{135}$	1.000	0.000

Table I. Part III. Healthy stage in the α-ketoglutarate dehydrogenase deficiency.

Variable	Flux	Flux groups	r^2	cv
x_1	$R00004MM$	x_1	0.999	0.000
x_2	$R00014MM$	$x_2, x_{11}, x_{18}, x_{71}$	0.997	0.000
x_3	$R00081MM$	$x_3, x_6, x_9, x_{20}, x_{22}, x_{23}$	1.000	0.000
x_4	$R00086MM$	$x_4, x_{58}, x_{91}, x_{113}$	1.000	0.024
x_5	$R00127MM$	$x_3, x_5, x_6, x_9, x_{20}$	0.994	0.000
x_6	$R00157MM$	$x_3, x_6, x_9, x_{20}, x_{22}, x_{23}$	1.000	n.a.
x_7	$R00205MM$	$x_7, x_{11}, x_{12}, x_{18}, x_{71}, x_{74}$**	1.000	1.289
x_8	$R00238MM$	$x_8, x_{14}, x_{16}, x_{128}$	1.000	0.047
x_9	$R00243MM$	$x_3, x_6, x_9, x_{20}, x_{22}, x_{23}$	1.000	n.a.
x_{10}	$R00245MM$	$x_{10}, x_{21}, x_{39}, x_{109}$	1.000	0.029
x_{11}	$R00256MM$	$x_7, x_{11}, x_{12}, x_{18}, x_{71}, x_{74}$**	0.997	1.289
x_{12}	$R00258MM$	$x_7, x_{11}, x_{12}, x_{18}, x_{70}, x_{71}$	1.000	0.000
x_{13}	$R00275MM$	$x_{13}, x_{57}, x_{65}, x_{78}, x_{107}$	1.000	0.042
x_{14}	$R00330MM$	$x_4, x_{14}, x_{107}, x_{128}$	1.000	0.062
x_{15}	$R00342MM$	$x_{15}, x_{61}, x_{87}, x_{110}$	1.000	0.049
x_{16}	$R00351MM$	$x_{16}, x_{21}, x_{88}, x_{109}$	1.000	0.062
x_{17}	$R00355MM$	$x_{11}, x_{17}, x_{60}, x_{70}, x_{72}, x_{74}$	0.998	0.000
x_{18}	$R00371MM$	$x_7, x_{11}, x_{18}, x_{71}$*	1.000	1.289
x_{19}	$R00388MM$	$x_{19}, x_{43}, x_{89}, x_{108}$	1.000	0.042
x_{20}	$R00430MM$	$x_3, x_6, x_9, x_{20}, x_{22}, x_{23}$	1.000	n.a.
x_{21}	$R00432MM$	$x_{21}, x_{37}, x_{91}, x_{105}$	1.000	0.062
x_{22}	$R00512MM$	$x_3, x_6, x_9, x_{20}, x_{22}, x_{23}$	1.000	n.a.
x_{23}	$R00551MM$	$x_3, x_6, x_9, x_{20}, x_{22}, x_{23}$	1.000	0.000
x_{24}	$R00572MM$	$x_3, x_6, x_9, x_{20}, x_{22}, x_{24}$	1.000	n.a.
x_{25}	$R00667MM$	$x_{25}, x_{29}, x_{41}, x_{51}$**	1.000	0.000
x_{26}	$R00705MM$	$x_3, x_6, x_9, x_{20}, x_{22}, x_{26}$	1.000	n.a.
x_{27}	$R00709MM$	$x_{27}, x_{61}, x_{87}, x_{98}, x_{107}$	1.000	0.062
x_{28}	$R00713MM$	x_{28}, x_{50}**	1.000	15.444
x_{29}	$R00716MM$	x_{29}, x_{41}, x_{93}*	1.000	16.882
x_{30}	$R00740MM$	$x_3, x_6, x_9, x_{20}, x_{22}, x_{30}$	1.000	n.a.
x_{31}	$R00830MM$	$x_3, x_6, x_9, x_{20}, x_{22}, x_{31}$	1.000	n.a.
x_{32}	$R00833MM$	$x_{32}, x_{84}, x_{126}, x_{127}, x_{131}$*	1.000	16.882
x_{33}	$R00851MM$	$x_3, x_6, x_9, x_{20}, x_{22}, x_{33}$	1.000	n.a.
x_{34}	$R00927MM$	$x_3, x_6, x_9, x_{20}, x_{22}, x_{34}$	1.000	n.a.
x_{35}	$R00941MM$	$x_3, x_6, x_9, x_{20}, x_{22}, x_{35}$	1.000	n.a.
x_{36}	$R00945MM$	x_{36}, x_{46}*	1.000	16.882
x_{37}	$R01082MM$	$x_{37}, x_{73}, x_{105}, x_{116}, x_{128}$	1.000	0.062
x_{38}	$R01175MM$	$x_{19}, x_{38}, x_{61}, x_{104}, x_{117}$*	1.000	0.111
x_{39}	$R01177MM$	$x_{10}, x_{19}, x_{39}, x_{57}, x_{105}$*	1.000	0.112
x_{40}	$R01214MM$	$x_{32}, x_{40}, x_{94}, x_{126}, x_{131}$**	1.000	16.882
x_{41}	$R01218MM$	$x_{25}, x_{29}, x_{41}, x_{51}$**	1.000	16.882

Table J. Part I. Inflammation stage in the α-ketoglutarate dehydrogenase deficiency. There are many groups with four or five fluxes linked by a functional relation, and four functional groups linked by a strong relation.

Variable	Flux	Flux groups	r^2	cv
x_{42}	$R01253MM$	$x_3, x_6, x_9, x_{20}, x_{22}, x_{42}$	1.000	0.000
x_{43}	$R01279MM$	$x_{15}, x_{43}, x_{108}, x_{115}$*	1.000	0.112
x_{44}	$R01280MM$	$x_{44}, x_{53}, x_{102}, x_{103}$*	1.000	0.335
x_{45}	$R01325MM$	$x_{19}, x_{45}, x_{90}, x_{110}$	1.000	0.062
x_{46}	$R01360MM$	$x_3, x_6, x_9, x_{20}, x_{22}, x_{46}$	1.000	0.000
x_{47}	$R01361MM$	$x_3, x_6, x_9, x_{20}, x_{22}, x_{47}$	1.000	0.000
x_{48}	$R01624MM$	$x_{48}, x_{96}, x_{101}, x_{121}, x_{125}$*	0.997	2.986
x_{49}	$R01626MM$	$x_{49}, x_{96}, x_{101}, x_{121}, x_{123}$*	0.997	0.641
x_{50}	$R01648MM$	x_{28}, x_{50}**	1.000	15.444
x_{51}	$R01655MM$	$x_{25}, x_{29}, x_{41}, x_{51}$**	1.000	16.882
x_{52}	$R01700MM$	$x_{52}, x_{88}, x_{89}, x_{104}, x_{115}$	1.000	0.062
x_{53}	$R01706MM$	$x_{44}, x_{53}, x_{103}, x_{124}$*	1.000	0.335
x_{54}	$R01799MM$	$x_3, x_6, x_9, x_{20}, x_{22}, x_{54}$	1.000	n.a.
x_{55}	$R01801MM$	$x_3, x_6, x_9, x_{20}, x_{22}, x_{55}$	1.000	n.a.
x_{56}	$R01859MM$	$x_3, x_6, x_9, x_{20}, x_{22}, x_{56}$	1.000	n.a.
x_{57}	$R01900MM$	$x_4, x_8, x_{10}, x_{13}, x_{57}$	1.000	0.062
x_{58}	$R01923MM$	$x_{15}, x_{58}, x_{91}, x_{108}, x_{128}$*	1.000	0.112
x_{59}	$R01939MM$	x_{59}	0.652	2.070
x_{60}	$R01940MM$	$x_{60}, x_{70}, x_{71}, x_{74}$	0.998	0.000
x_{61}	$R01975MM$	$x_{61}, x_{89}, x_{90}, x_{104}, x_{108}$	1.000	0.088
x_{62}	$R01978MM$	$x_3, x_6, x_9, x_{20}, x_{22}, x_{62}$	1.000	0.000
x_{63}	$R02030MM$	$x_3, x_6, x_9, x_{20}, x_{22}, x_{63}$	1.000	n.a.
x_{64}	$R02161MM$	$x_3, x_6, x_9, x_{20}, x_{64}$	n.a.	0.000
x_{65}	$R02163MM$	$x_{14}, x_{65}, x_{86}, x_{116}, x_{117}$	1.000	0.042
x_{66}	$R02164MM$	$x_{38}, x_{66}, x_{85}, x_{98}, x_{106}$	1.000	0.062
x_{67}	$R02199MM$	$x_3, x_6, x_9, x_{20}, x_{22}, x_{67}$	1.000	n.a.
x_{68}	$R02241MM$	$x_3, x_6, x_9, x_{20}, x_{22}, x_{68}$	1.000	n.a.
x_{69}	$R02313MM$	x_{26}, x_{69}*	1.000	16.854
x_{70}	$R02487MM$	$x_7, x_{18}, x_{60}, x_{70}, x_{71}, x_{74}$	0.993	0.000
x_{71}	$R02529MM$	$x_7, x_{11}, x_{12}, x_{18}, x_{70}, x_{71}$**	1.000	1.289
x_{72}	$R02569MM$	x_{11}, x_{71}, x_{72}	0.997	0.000
x_{73}	$R02570MM$	$x_{73}, x_{86}, x_{88}, x_{112}, x_{114}$	1.000	0.062
x_{74}	$R02571MM$	$x_{11}, x_{17}, x_{18}, x_{60}, x_{70}, x_{74}$	0.998	0.000
x_{75}	$R02661MM$	x_{75}	0.767	17.569
x_{76}	$R02662MM$	x_{33}, x_{76}, x_{79}*	1.000	16.882
x_{77}	$R02765MM$	$x_3, x_6, x_9, x_{20}, x_{22}, x_{77}$	1.000	n.a.
x_{78}	$R03026MM$	$x_{13}, x_{15}, x_{78}, x_{90}, x_{91}$	1.000	0.088
x_{79}	$R03102MM$	$x_3, x_6, x_9, x_{20}, x_{22}, x_{79}$	1.000	n.a.
x_{80}	$R03172MM$	$x_3, x_6, x_9, x_{20}, x_{22}, x_{80}$	1.000	n.a.
x_{81}	$R03174MM$	$x_3, x_6, x_9, x_{20}, x_{22}, x_{81}$	1.000	n.a.
x_{82}	$R03270MM$	$x_{11}, x_{18}, x_{71}, x_{82}$	0.997	0.000
x_{83}	$R03314MM$	$x_3, x_6, x_9, x_{20}, x_{22}, x_{83}$	1.000	0.000
x_{84}	$R03381MM$	$x_{32}, x_{40}, x_{84}, x_{126}, x_{127}$*	1.000	16.882
x_{85}	$R03777MM$	$x_{19}, x_{38}, x_{85}, x_{98}, x_{108}$*	1.000	0.112
x_{86}	$R03778MM$	$x_{16}, x_{57}, x_{86}, x_{107}, x_{111}$*	1.000	0.112
x_{87}	$R03857MM$	$x_{10}, x_{38}, x_{43}, x_{87}, x_{106}$*	1.000	0.112
x_{88}	$R03858MM$	$x_{66}, x_{88}, x_{107}, x_{116}$*	1.000	0.112

Table J. Part II. Inflammation stage in the α-ketoglutarate dehydrogenase deficiency.

Variable	Flux	Flux groups	r^2	cv
x_{89}	$R03990MM$	$x_{15}, x_{66}, x_{89}, x_{134}$*	1.000	0.112
x_{90}	$R03991MM$	$x_{14}, x_{16}, x_{39}, x_{45}, x_{90}$*	1.000	0.112
x_{91}	$R04170MM$	$x_{91}, x_{111}, x_{114}, x_{128}$*	1.000	0.112
x_{92}	$R04203MM$	$x_{3}, x_{6}, x_{9}, x_{20}, x_{22}, x_{92}$	1.000	n.a.
x_{93}	$R04204MM$	$x_{3}, x_{6}, x_{9}, x_{20}, x_{22}, x_{93}$	1.000	n.a.
x_{94}	$R04224MM$	$x_{32}, x_{84}, x_{94}, x_{126}, x_{127}$*	1.000	16.882
x_{95}	$R04355MM$	x_{95}*	0.946	2.978
x_{96}	$R04428MM$	$x_{48}, x_{96}, x_{99}, x_{101}, x_{121}$*	1.000	2.986
x_{97}	$R04430MM$	$x_{48}, x_{97}, x_{99}, x_{120}, x_{122}$*	0.997	2.986
x_{98}	$R04433MM$	$x_{19}, x_{27}, x_{89}, x_{98}, x_{112}$*	1.000	0.108
x_{99}	$R04533MM$	$x_{48}, x_{97}, x_{99}, x_{122}$*	0.999	2.986
x_{100}	$R04536MM$	$x_{100}, x_{120}, x_{121}, x_{122}$*	1.000	2.986
x_{101}	$R04537MM$	$x_{100}, x_{101}, x_{103}, x_{121}$*	1.000	2.986
x_{102}	$R04543MM$	$x_{100}, x_{102}, x_{103}, x_{120}, x_{125}$*	0.999	0.335
x_{103}	$R04544MM$	$x_{102}, x_{103}, x_{124}, x_{125}$*	1.000	0.335
x_{104}	$R04737MM$	$x_{27}, x_{43}, x_{45}, x_{104}, x_{112}$*	1.000	0.112
x_{105}	$R04738MM$	$x_{65}, x_{86}, x_{90}, x_{91}, x_{105}$*	1.000	0.112
x_{106}	$R04739MM$	$x_{38}, x_{87}, x_{98}, x_{106}, x_{110}$*	1.000	0.112
x_{107}	$R04740MM$	$x_{10}, x_{16}, x_{37}, x_{107}, x_{109}$*	1.000	0.112
x_{108}	$R04741MM$	$x_{19}, x_{89}, x_{106}, x_{108}$*	1.000	0.112
x_{109}	$R04742MM$	$x_{10}, x_{39}, x_{61}, x_{88}, x_{109}$*	1.000	0.112
x_{110}	$R04743MM$	$x_{15}, x_{27}, x_{110}, x_{134}$*	1.000	0.112
x_{111}	$R04744MM$	$x_{57}, x_{58}, x_{86}, x_{90}, x_{111}$*	1.000	0.112
x_{112}	$R04745MM$	$x_{45}, x_{85}, x_{91}, x_{107}, x_{112}$*	1.000	0.112
x_{113}	$R04746MM$	$x_{10}, x_{58}, x_{88}, x_{112}, x_{113}$*	1.000	0.112
x_{114}	$R04747MM$	$x_{13}, x_{21}, x_{87}, x_{114}, x_{116}$*	1.000	0.112
x_{115}	$R04748MM$	$x_{15}, x_{89}, x_{104}, x_{115}$*	1.000	0.112
x_{116}	$R04749MM$	$x_{13}, x_{16}, x_{65}, x_{114}, x_{116}$*	1.000	0.112
x_{117}	$R04751MM$	$x_{43}, x_{45}, x_{104}, x_{106}, x_{117}$*	1.000	0.112
x_{118}	$R04754MM$	$x_{19}, x_{61}, x_{115}, x_{117}, x_{118}$*	1.000	0.112
x_{119}	$R04952MM$	x_{119}*	0.999	2.976
x_{120}	$R04953MM$	$x_{97}, x_{100}, x_{120}, x_{122}$*	1.000	2.986
x_{121}	$R04954MM$	$x_{96}, x_{97}, x_{101}, x_{121}, x_{125}$*	1.000	2.986
x_{122}	$R04956MM$	$x_{100}, x_{120}, x_{122}, x_{123}$*	1.000	2.986
x_{123}	$R04959MM$	$x_{100}, x_{101}, x_{120}, x_{122}, x_{123}$*	0.998	2.986
x_{124}	$R04968MM$	$x_{101}, x_{102}, x_{103}, x_{123}, x_{124}$*	1.000	0.335
x_{125}	$R04970MM$	$x_{97}, x_{99}, x_{102}, x_{103}, x_{125}$*	0.998	0.335
x_{126}	$R05064MM$	$x_{32}, x_{40}, x_{94}, x_{126}, x_{131}$**	1.000	16.882
x_{127}	$R05066MM$	$x_{32}, x_{40}, x_{126}, x_{127}, x_{131}$*	1.000	16.882
x_{128}	$R07162MM$	$x_{13}, x_{14}, x_{39}, x_{86}, x_{128}$	1.000	0.042
x_{129}	$R07390MM$	$x_{3}, x_{6}, x_{9}, x_{20}, x_{22}, x_{129}$	1.000	n.a.
x_{130}	$R07599MM$	$x_{3}, x_{6}, x_{9}, x_{20}, x_{22}, x_{130}$	1.000	n.a.
x_{131}	$R07600MM$	$x_{32}, x_{40}, x_{84}, x_{126}, x_{131}$*	1.000	16.882
x_{132}	$R07603MM$	$x_{3}, x_{6}, x_{9}, x_{20}, x_{22}, x_{132}$	1.000	n.a.
x_{133}	$R07604MM$	$x_{3}, x_{6}, x_{9}, x_{20}, x_{22}, x_{133}$	1.000	n.a.
x_{134}	$R07618MM$	$x_{52}, x_{73}, x_{106}, x_{134}$	1.000	0.049
x_{135}	$R08157MM$	$x_{3}, x_{6}, x_{9}, x_{20}, x_{22}, x_{135}$	1.000	0.000

Table J. Part III. Inflammation stage in the α-ketoglutarate dehydrogenase deficiency.

Variable	Flux	Flux groups	r^2	cv
x_1	$R00004MM$	x_1, x_{42}	1.000	0.000
x_2	$R00014MM$	x_2, x_{72}, x_{82}	1.000	0.000
x_3	$R00081MM$	$x_3, x_6, x_9, x_{23}, x_{26}$	1.000	0.000
x_4	$R00086MM$	$x_4, x_{44}, x_{57}, x_{65}, x_{78}, x_{87}$	1.000	0.024
x_5	$R00127MM$	$x_5, x_6, x_9, x_{23}, x_{26}$	0.999	0.000
x_6	$R00157MM$	$x_6, x_9, x_{23}, x_{26}, x_{30}, x_{31}$	1.000	n.a.
x_7	$R00205MM$	$x_7, x_{11}, x_{17}, x_{18}, x_{71}, x_{74}$*	1.000	1.622
x_8	$R00238MM$	$x_8, x_{39}, x_{44}, x_{73}, x_{85}, x_{107}$	1.000	0.038
x_9	$R00243MM$	$x_6, x_9, x_{23}, x_{26}, x_{30}, x_{31}$	1.000	n.a.
x_{10}	$R00245MM$	$x_8, x_{10}, x_{44}, x_{91}, x_{105}, x_{114}$	1.000	0.031
x_{11}	$R00256MM$	$x_7, x_{11}, x_{18}, x_{60}, x_{71}, x_{74}$*	1.000	1.622
x_{12}	$R00258MM$	$x_{12}, x_{60}, x_{70}, x_{74}$	0.990	0.006
x_{13}	$R00275MM$	$x_{13}, x_{53}, x_{57}, x_{73}, x_{104}, x_{107}$	1.000	0.046
x_{14}	$R00330MM$	$x_{14}, x_{89}, x_{105}, x_{114}$*	1.000	1.043
x_{15}	$R00342MM$	$x_{15}, x_{38}, x_{110}, x_{111}, x_{134}$	1.000	0.040
x_{16}	$R00351MM$	$x_{16}, x_{39}, x_{44}, x_{90}, x_{107}, x_{116}$	1.000	0.048
x_{17}	$R00355MM$	$x_{12}, x_{17}, x_{60}, x_{70}, x_{74}$	0.999	0.000
x_{18}	$R00371MM$	$x_7, x_{11}, x_{18}, x_{70}, x_{71}, x_{74}$*	1.000	1.622
x_{19}	$R00388MM$	$x_{19}, x_{37}, x_{44}, x_{85}, x_{98}, x_{118}$	1.000	0.046
x_{20}	$R00430MM$	x_{20}, x_{24}**	1.000	1.068
x_{21}	$R00432MM$	$x_{21}, x_{53}, x_{58}, x_{73}, x_{78}, x_{88}$	1.000	0.048
x_{22}	$R00512MM$	x_{22}, x_{63}**	1.000	5.598
x_{23}	$R00551MM$	$x_6, x_9, x_{23}, x_{26}, x_{30}, x_{31}$	1.000	0.000
x_{24}	$R00572MM$	x_{20}, x_{24}**	1.000	1.068
x_{25}	$R00667MM$	x_{25}, x_{35}, x_{47}	1.000	0.000
x_{26}	$R00705MM$	$x_6, x_9, x_{23}, x_{26}, x_{30}, x_{31}$	1.000	n.a.
x_{27}	$R00709MM$	$x_{27}, x_{43}, x_{53}, x_{61}, x_{89}, x_{108}$	1.000	0.048
x_{28}	$R00713MM$	x_{28}, x_{50}**	1.000	2.148
x_{29}	$R00716MM$	x_{29}*	0.999	1.829
x_{30}	$R00740MM$	$x_6, x_9, x_{23}, x_{26}, x_{30}, x_{31}$	1.000	n.a.
x_{31}	$R00830MM$	$x_6, x_9, x_{23}, x_{26}, x_{30}, x_{31}$	1.000	n.a.
x_{32}	$R00833MM$	$x_{32}, x_{84}, x_{94}, x_{127}$*	1.000	9.574
x_{33}	$R00851MM$	x_{33}, x_{54}*	1.000	5.611
x_{34}	$R00927MM$	$x_{34}, x_{77}, x_{81}, x_{93}$**	1.000	16.340
x_{35}	$R00941MM$	$x_6, x_9, x_{23}, x_{26}, x_{30}, x_{35}$	1.000	n.a.
x_{36}	$R00945MM$	$x_6, x_9, x_{23}, x_{26}, x_{30}, x_{36}$	1.000	n.a.
x_{37}	$R01082MM$	$x_{10}, x_{37}, x_{107}, x_{116}$	1.000	0.048
x_{38}	$R01175MM$	$x_{38}, x_{44}, x_{61}, x_{78}, x_{87}, x_{98}$	1.000	0.076
x_{39}	$R01177MM$	$x_8, x_{21}, x_{37}, x_{39}, x_{44}, x_{90}$	1.000	0.076
x_{40}	$R01214MM$	x_{40}, x_{130}, x_{131}**	1.000	9.241
x_{41}	$R01218MM$	$x_6, x_9, x_{23}, x_{26}, x_{30}, x_{41}$	1.000	n.a.

Table K. Part I. Pathological stage in the α-ketoglutarate dehydrogenase deficiency. Six groups have been detected by the algorithm in this stage of the disease. Furthermore, there are numerous groups involving few fluxes.

Variable	Flux	Flux groups	r^2	cv
x_{42}	$R01253MM$	$x_6, x_9, x_{23}, x_{26}, x_{30}, x_{42}$	1.000	0.000
x_{43}	$R01279MM$	$x_4, x_{38}, x_{43}, x_{53}, x_{85}, x_{118}$	1.000	0.076
x_{44}	$R01280MM$	x_{44}, x_{98}*	0.998	1.466
x_{45}	$R01325MM$	$x_{38}, x_{45}, x_{53}, x_{89}, x_{98}, x_{118}$	1.000	0.048
x_{46}	$R01360MM$	x_{46}	1.000	0.000
x_{47}	$R01361MM$	$x_6, x_9, x_{23}, x_{26}, x_{30}, x_{47}$	1.000	0.000
x_{48}	$R01624MM$	x_{48}, x_{121}*	1.000	0.685
x_{49}	$R01626MM$	x_{49}*	0.997	4.235
x_{50}	$R01648MM$	x_{28}, x_{50}**	1.000	2.148
x_{51}	$R01655MM$	$x_6, x_9, x_{23}, x_{26}, x_{30}, x_{51}$	1.000	n.a.
x_{52}	$R01700MM$	$x_{27}, x_{52}, x_{53}, x_{85}, x_{108}, x_{118}$	1.000	0.048
x_{53}	$R01706MM$	x_{44}, x_{53}*	1.000	1.466
x_{54}	$R01799MM$	x_{22}, x_{54}, x_{63}*	1.000	5.608
x_{55}	$R01801MM$	$x_6, x_9, x_{23}, x_{26}, x_{30}, x_{55}$	1.000	n.a.
x_{56}	$R01859MM$	$x_{31}, x_{56}, x_{132}, x_{133}$*	1.000	16.340
x_{57}	$R01900MM$	$x_{15}, x_{39}, x_{53}, x_{57}, x_{90}, x_{107}$	1.000	0.048
x_{58}	$R01923MM$	$x_{58}, x_{88}, x_{90}, x_{109}, x_{128}$	1.000	0.076
x_{59}	$R01939MM$	x_{59}*	0.999	5.480
x_{60}	$R01940MM$	$x_{12}, x_{17}, x_{60}, x_{70}, x_{74}$	0.999	0.005
x_{61}	$R01975MM$	$x_{19}, x_{21}, x_{44}, x_{61}, x_{85}, x_{134}$	1.000	0.064
x_{62}	$R01978MM$	$x_6, x_9, x_{23}, x_{26}, x_{62}$	0.999	0.000
x_{63}	$R02030MM$	x_{22}, x_{63}**	1.000	5.590
x_{64}	$R02161MM$	$x_6, x_9, x_{23}, x_{26}, x_{64}$	n.a.	0.000
x_{65}	$R02163MM$	$x_{53}, x_{65}, x_{85}, x_{88}, x_{90}, x_{109}$	1.000	0.046
x_{66}	$R02164MM$	$x_{27}, x_{44}, x_{66}, x_{85}, x_{89}, x_{117}$	1.000	0.048
x_{67}	$R02199MM$	x_{30}, x_{67}, x_{133}*	1.000	16.340
x_{68}	$R02241MM$	x_{68}, x_{129}**	1.000	5.608
x_{69}	$R02313MM$	x_{69}*	0.999	1.829
x_{70}	$R02487MM$	$x_{17}, x_{60}, x_{70}, x_{74}$	1.000	0.005
x_{71}	$R02529MM$	$x_7, x_{11}, x_{18}, x_{71}, x_{74}$*	1.000	1.622
x_{72}	$R02569MM$	x_2, x_{72}, x_{82}	1.000	0.000
x_{73}	$R02570MM$	$x_{53}, x_{73}, x_{111}, x_{114}, x_{116}, x_{128}$	1.000	0.048
x_{74}	$R02571MM$	$x_{17}, x_{60}, x_{70}, x_{74}$	1.000	0.005
x_{75}	$R02661MM$	x_{75}*	1.000	9.241
x_{76}	$R02662MM$	x_{76}*	0.998	9.241
x_{77}	$R02765MM$	$x_{34}, x_{77}, x_{81}, x_{93}$**	1.000	16.340
x_{78}	$R03026MM$	$x_4, x_8, x_{78}, x_{114}$	1.000	0.064
x_{79}	$R03102MM$	$x_6, x_9, x_{23}, x_{26}, x_{30}, x_{79}$	1.000	n.a.
x_{80}	$R03172MM$	x_{52}, x_{80}*	0.996	16.340
x_{81}	$R03174MM$	$x_{34}, x_{77}, x_{81}, x_{93}$**	1.000	16.340
x_{82}	$R03270MM$	x_2, x_{72}, x_{82}	1.000	0.000
x_{83}	$R03314MM$	$x_6, x_9, x_{23}, x_{26}, x_{83}$	1.000	0.000
x_{84}	$R03381MM$	$x_{32}, x_{84}, x_{126}, x_{127}$*	1.000	9.241
x_{85}	$R03777MM$	$x_{15}, x_{38}, x_{44}, x_{85}, x_{89}, x_{108}$	1.000	0.076
x_{86}	$R03778MM$	$x_4, x_{53}, x_{65}, x_{86}, x_{114}, x_{134}$	1.000	0.076
x_{87}	$R03857MM$	$x_{43}, x_{44}, x_{66}, x_{87}, x_{98}, x_{112}$	1.000	0.076
x_{88}	$R03858MM$	$x_{53}, x_{57}, x_{78}, x_{88}, x_{105}, x_{116}$	1.000	0.076

Table K. Part II. Pathological stage in the α-ketoglutarate dehydrogenase deficiency.

Variable	Flux	Flux groups	r^2	cv
x_{89}	$R03990MM$	$x_{19}, x_{27}, x_{87}, x_{89}, x_{115}$	1.000	0.076
x_{90}	$R03991MM$	$x_{37}, x_{73}, x_{90}, x_{116}$	1.000	0.076
x_{91}	$R04170MM$	$x_{4}, x_{57}, x_{65}, x_{91}, x_{105}$	1.000	0.076
x_{92}	$R04203MM$	$x_{47}, x_{92}, x_{132}, x_{133}$*	1.000	16.340
x_{93}	$R04204MM$	$x_{9}, x_{34}, x_{77}, x_{93}, x_{135}$*	0.999	16.340
x_{94}	$R04224MM$	x_{84}, x_{94}, x_{126}*	1.000	9.241
x_{95}	$R04355MM$	x_{95}*	1.000	0.685
x_{96}	$R04428MM$	x_{48}, x_{96}*	1.000	0.685
x_{97}	$R04430MM$	x_{97}, x_{100}*	1.000	0.685
x_{98}	$R04433MM$	$x_{27}, x_{52}, x_{98}, x_{112}$	1.000	0.074
x_{99}	$R04533MM$	$x_{97}, x_{99}, x_{100}, x_{120}$*	1.000	0.685
x_{100}	$R04536MM$	x_{100}, x_{123}*	1.000	0.685
x_{101}	$R04537MM$	x_{96}, x_{101}*	1.000	0.685
x_{102}	$R04543MM$	$x_{102}, x_{124}, x_{125}$*	1.000	1.466
x_{103}	$R04544MM$	x_{103}, x_{124}**	1.000	1.466
x_{104}	$R04737MM$	$x_{44}, x_{45}, x_{61}, x_{104}, x_{107}, x_{118}$	1.000	0.076
x_{105}	$R04738MM$	$x_{10}, x_{39}, x_{44}, x_{73}, x_{91}, x_{105}$	1.000	0.076
x_{106}	$R04739MM$	$x_{53}, x_{61}, x_{89}, x_{106}, x_{118}, x_{134}$	1.000	0.076
x_{107}	$R04740MM$	$x_{13}, x_{53}, x_{65}, x_{86}, x_{90}, x_{107}$	1.000	0.076
x_{108}	$R04741MM$	$x_{38}, x_{53}, x_{89}, x_{98}, x_{104}, x_{108}$	1.000	0.076
x_{109}	$R04742MM$	$x_{39}, x_{44}, x_{65}, x_{90}, x_{109}, x_{113}$	1.000	0.076
x_{110}	$R04743MM$	$x_{44}, x_{61}, x_{89}, x_{98}, x_{106}, x_{110}$	1.000	0.076
x_{111}	$R04744MM$	$x_{10}, x_{44}, x_{57}, x_{86}, x_{91}, x_{111}$	1.000	0.076
x_{112}	$R04745MM$	$x_{52}, x_{53}, x_{85}, x_{110}, x_{112}, x_{118}$	1.000	0.076
x_{113}	$R04746MM$	$x_{44}, x_{58}, x_{78}, x_{90}, x_{105}, x_{113}$	1.000	0.076
x_{114}	$R04747MM$	$x_{53}, x_{78}, x_{89}, x_{105}, x_{113}, x_{114}$	1.000	0.076
x_{115}	$R04748MM$	$x_{52}, x_{53}, x_{88}, x_{108}, x_{115}, x_{118}$	1.000	0.076
x_{116}	$R04749MM$	$x_{13}, x_{16}, x_{53}, x_{66}, x_{113}, x_{116}$	1.000	0.076
x_{117}	$R04751MM$	$x_{66}, x_{80}, x_{87}, x_{98}, x_{117}, x_{118}$	1.000	0.076
x_{118}	$R04754MM$	$x_{19}, x_{45}, x_{53}, x_{85}, x_{89}, x_{118}$	1.000	0.076
x_{119}	$R04952MM$	$x_{9}, x_{41}, x_{79}, x_{119}$*	0.998	0.685
x_{120}	$R04953MM$	$x_{100}, x_{120}, x_{122}$*	1.000	0.685
x_{121}	$R04954MM$	x_{96}, x_{101}, x_{121}*	1.000	0.685
x_{122}	$R04956MM$	x_{120}, x_{122}*	1.000	0.685
x_{123}	$R04959MM$	x_{97}, x_{100}, x_{123}*	1.000	0.685
x_{124}	$R04968MM$	x_{103}, x_{124}**	1.000	1.466
x_{125}	$R04970MM$	$x_{102}, x_{103}, x_{125}$*	1.000	1.466
x_{126}	$R05064MM$	x_{32}, x_{126}*	1.000	9.241
x_{127}	$R05066MM$	x_{94}, x_{127}*	1.000	9.241
x_{128}	$R07162MM$	$x_{10}, x_{16}, x_{88}, x_{128}$	1.000	0.046
x_{129}	$R07390MM$	x_{68}, x_{129}**	1.000	5.590
x_{130}	$R07599MM$	x_{130}, x_{131}*	1.000	9.241
x_{131}	$R07600MM$	x_{40}, x_{130}, x_{131}**	1.000	9.241
x_{132}	$R07603MM$	$x_{56}, x_{67}, x_{92}, x_{132}, x_{133}$*	1.000	16.340
x_{133}	$R07604MM$	$x_{56}, x_{92}, x_{132}, x_{133}$*	1.000	16.340
x_{134}	$R07618MM$	$x_{53}, x_{61}, x_{85}, x_{108}, x_{115}, x_{134}$	1.000	0.039
x_{135}	$R08157MM$	$x_{6}, x_{9}, x_{23}, x_{26}, x_{30}, x_{135}$	1.000	0.000

Table K. Part III. Pathological stage in the α-ketoglutarate dehydrogenase deficiency.

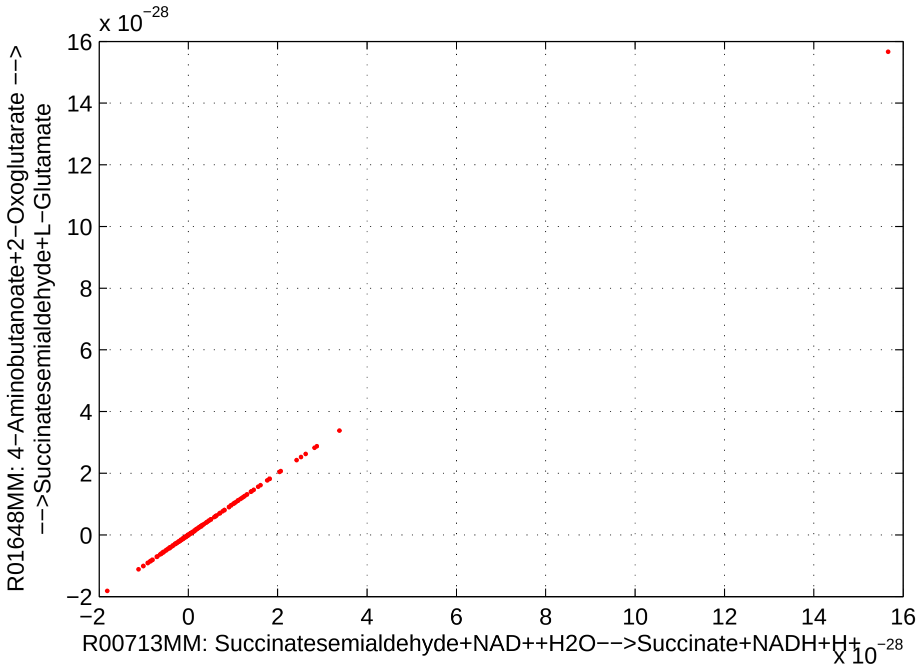

Figure S28. α-ketoglutarate dehydrogenase deficiency - healthy stage. Functional relation β (y axis) found for the two fluxes R00713MM and R01648MM (x axis) [μmol min^{-1} gDW^{-1}] in the mitochondrial FBA model [2].

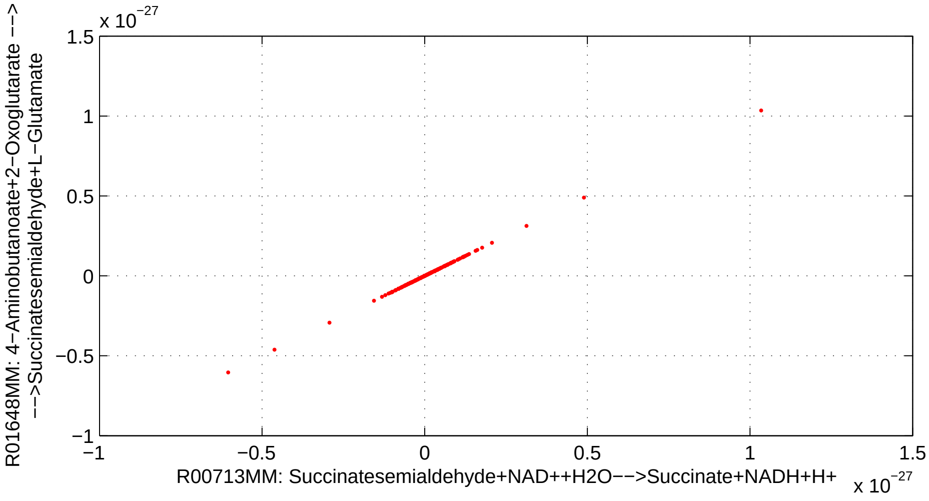

Figure S29. α-ketoglutarate dehydrogenase deficiency - inflammation stage. Functional relation β (y axis) found for the two fluxes R00713MM and R01648MM (x axis) [μmol min^{-1} gDW^{-1}] in the mitochondrial FBA model [2].

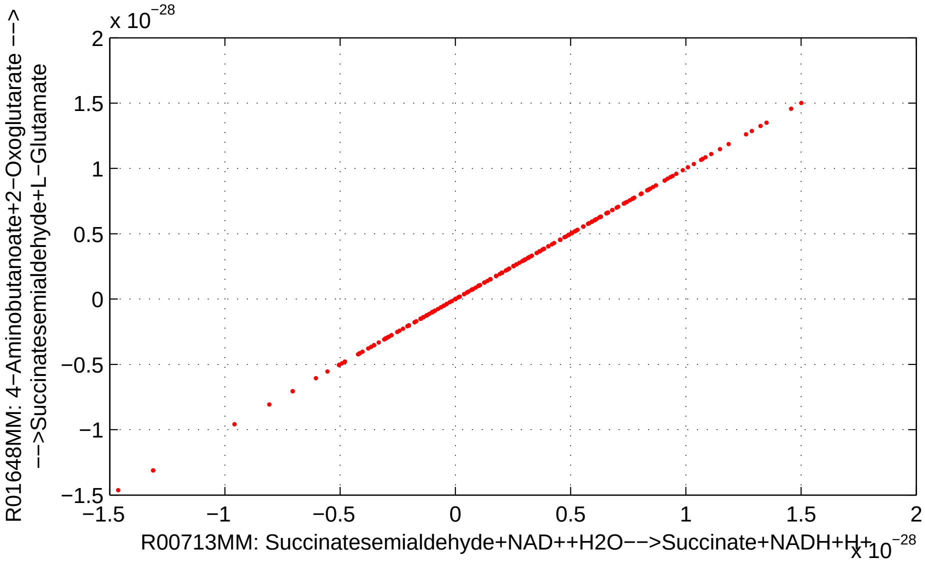

Figure S30. α-ketoglutarate dehydrogenase deficiency - pathological stage. Functional relation β (y axis) found for the two fluxes R00713MM and R01648MM (x axis) [μmol min^{-1} gDW^{-1}] in the mitochondrial FBA model [2]. The linear functional relation is conserved across stages.

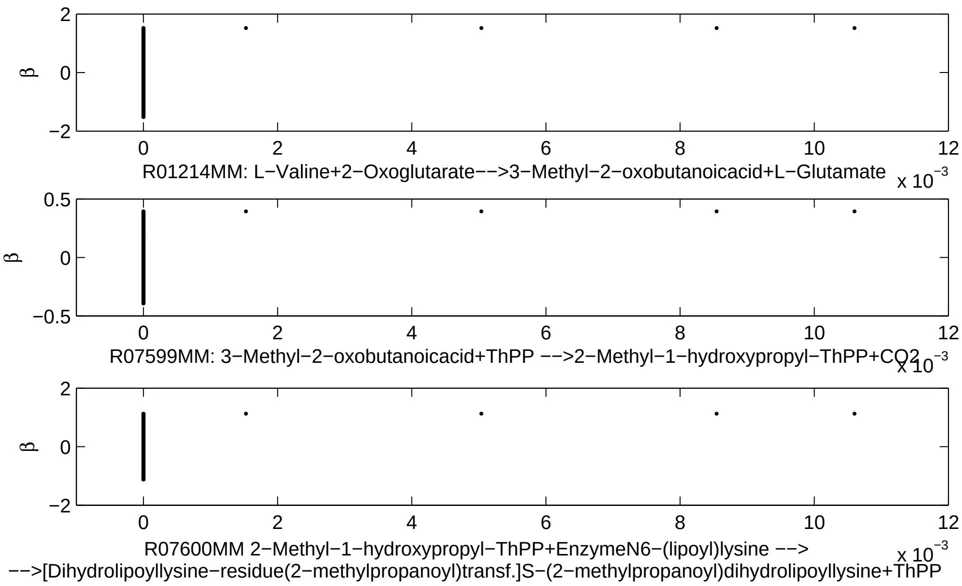

Figure S31. α-ketoglutarate dehydrogenase deficiency - pathological stage. Optimal transformations β (y axis) found for the three fluxes R01214MM, R07599MM, and R07600MM (x axis) [μmol min^{-1} gDW^{-1}] in the mitochondrial FBA model [2]. The order in which we observe variations in the optimal transformation is R01214MM > R07600MM > R07599MM.

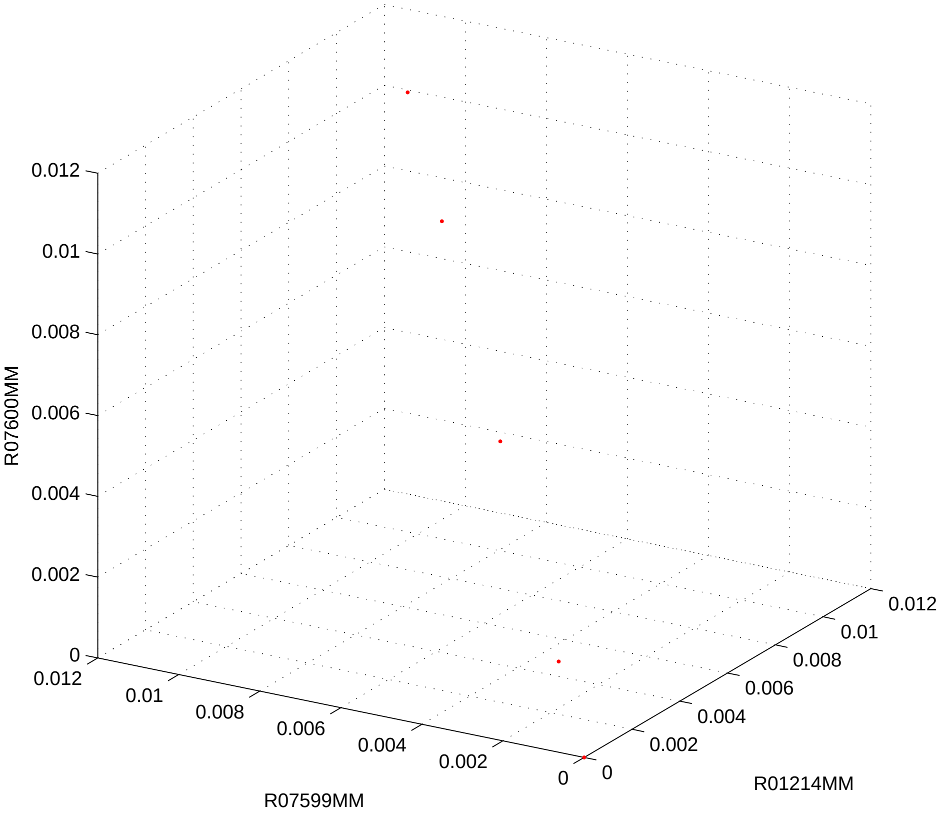

Figure S32. α-ketoglutarate dehydrogenase deficiency - pathological stage. Functional relation β (y axis) found for the three fluxes R01214MM, R07599MM, and R07600MM (x axis) [μmol min^{-1} gDW^{-1}] in the mitochondrial FBA model [2].

References

1. Chang R, Ghamsari L, Manichaikul A, Hom E, Balaji S, et al. (2011) Metabolic network reconstruction of chlamydomonas offers insight into light-driven algal metabolism. Molecular systems biology 7: 518.

2. Smith A, Robinson A (2011) A metabolic model of the mitochondrion and its use in modelling diseases of the tricarboxylic acid cycle. BMC systems biology 5: 102.
