# Supplementary material for: Multi-Target Analysis and Design of Mitochondrial Metabolism
Source: PLoS One. 2015 Sep 16;10(9):e0133825. doi: 10.1371/journal.pone.0133825 (PMC4574446; doi:10.1371/journal.pone.0133825)
Supplement: S1 Code — (ZIP) [file pone.0133825.s004.zip › source code for publication/Identifiability Analysis on monogenic diseases/Alfa-ketoglutarate dehydrogenase deficiency/tabella latex - pathological.docx]

| $x_1$ | & | $ | R00004MM | $ | & | $ | x_{1},x_{42 | } |  | $ | & | 1.000 | & | 0.000 | \\ |
| --- | --- | --- | --- | --- | --- | --- | --- | --- | --- | --- | --- | --- | --- | --- | --- |
| $x_2$ | & | $ | R00014MM | $ | & | $ | x_{2},x_{72},x_{82 | } |  | $ | & | 1.000 | & | 0.000 | \\ |
| $x_3$ | & | $ | R00081MM | $ | & | $ | x_{3},x_{6},x_{9},x_{23},x_{26 | } |  | $ | & | 1.000 | & | 0.000 | \\ |
| $x_4$ | & | $ | R00086MM | $ | & | $ | x_{4},x_{44},x_{57},x_{65},x_{78},x_{87 | } |  | $ | & | 1.000 | & | 0.024 | \\ |
| $x_5$ | & | $ | R00127MM | $ | & | $ | x_{5},x_{6},x_{9},x_{23},x_{26 | } |  | $ | & | 0.999 | & | 0.000 | \\ |
| $x_6$ | & | $ | R00157MM | $ | & | $ | x_{6},x_{9},x_{23},x_{26},x_{30},x_{31 | } |  | $ | & | 1.000 | & | NaN | \\ |
| $x_7$ | & | $ | R00205MM | $ | & | $ | x_{7},x_{11},x_{17},x_{18},x_{71},x_{74 | } | * | $ | & | 1.000 | & | 1.622 | \\ |
| $x_8$ | & | $ | R00238MM | $ | & | $ | x_{8},x_{39},x_{44},x_{73},x_{85},x_{107 | } |  | $ | & | 1.000 | & | 0.038 | \\ |
| $x_9$ | & | $ | R00243MM | $ | & | $ | x_{6},x_{9},x_{23},x_{26},x_{30},x_{31 | } |  | $ | & | 1.000 | & | NaN | \\ |
| $x_{10}$ | & | $ | R00245MM | $ | & | $ | x_{8},x_{10},x_{44},x_{91},x_{105},x_{114 | } |  | $ | & | 1.000 | & | 0.031 | \\ |
| $x_{11}$ | & | $ | R00256MM | $ | & | $ | x_{7},x_{11},x_{18},x_{60},x_{71},x_{74 | } | * | $ | & | 1.000 | & | 1.622 | \\ |
| $x_{12}$ | & | $ | R00258MM | $ | & | $ | x_{12},x_{60},x_{70},x_{74 | } |  | $ | & | 0.990 | & | 0.006 | \\ |
| $x_{13}$ | & | $ | R00275MM | $ | & | $ | x_{13},x_{53},x_{57},x_{73},x_{104},x_{107 | } |  | $ | & | 1.000 | & | 0.046 | \\ |
| $x_{14}$ | & | $ | R00330MM | $ | & | $ | x_{14},x_{89},x_{105},x_{114 | } | * | $ | & | 1.000 | & | 1.043 | \\ |
| $x_{15}$ | & | $ | R00342MM | $ | & | $ | x_{15},x_{38},x_{110},x_{111},x_{134 | } |  | $ | & | 1.000 | & | 0.040 | \\ |
| $x_{16}$ | & | $ | R00351MM | $ | & | $ | x_{16},x_{39},x_{44},x_{90},x_{107},x_{116 | } |  | $ | & | 1.000 | & | 0.048 | \\ |
| $x_{17}$ | & | $ | R00355MM | $ | & | $ | x_{12},x_{17},x_{60},x_{70},x_{74 | } |  | $ | & | 0.999 | & | 0.000 | \\ |
| $x_{18}$ | & | $ | R00371MM | $ | & | $ | x_{7},x_{11},x_{18},x_{70},x_{71},x_{74 | } | * | $ | & | 1.000 | & | 1.622 | \\ |
| $x_{19}$ | & | $ | R00388MM | $ | & | $ | x_{19},x_{37},x_{44},x_{85},x_{98},x_{118 | } |  | $ | & | 1.000 | & | 0.046 | \\ |
| $x_{20}$ | & | $ | R00430MM | $ | & | $ | x_{20},x_{24 | } | ** | $ | & | 1.000 | & | 1.068 | \\ |
| $x_{21}$ | & | $ | R00432MM | $ | & | $ | x_{21},x_{53},x_{58},x_{73},x_{78},x_{88 | } |  | $ | & | 1.000 | & | 0.048 | \\ |
| $x_{22}$ | & | $ | R00512MM | $ | & | $ | x_{22},x_{63 | } | ** | $ | & | 1.000 | & | 5.598 | \\ |
| $x_{23}$ | & | $ | R00551MM | $ | & | $ | x_{6},x_{9},x_{23},x_{26},x_{30},x_{31 | } |  | $ | & | 1.000 | & | 0.000 | \\ |
| $x_{24}$ | & | $ | R00572MM | $ | & | $ | x_{20},x_{24 | } | ** | $ | & | 1.000 | & | 1.068 | \\ |
| $x_{25}$ | & | $ | R00667MM | $ | & | $ | x_{25},x_{35},x_{47 | } |  | $ | & | 1.000 | & | 0.000 | \\ |
| $x_{26}$ | & | $ | R00705MM | $ | & | $ | x_{6},x_{9},x_{23},x_{26},x_{30},x_{31 | } |  | $ | & | 1.000 | & | NaN | \\ |
| $x_{27}$ | & | $ | R00709MM | $ | & | $ | x_{27},x_{43},x_{53},x_{61},x_{89},x_{108 | } |  | $ | & | 1.000 | & | 0.048 | \\ |
| $x_{28}$ | & | $ | R00713MM | $ | & | $ | x_{28},x_{50 | } | ** | $ | & | 1.000 | & | 2.148 | \\ |
| $x_{29}$ | & | $ | R00716MM | $ | & | $ | x_{29 | } | * | $ | & | 0.999 | & | 1.829 | \\ |
| $x_{30}$ | & | $ | R00740MM | $ | & | $ | x_{6},x_{9},x_{23},x_{26},x_{30},x_{31 | } |  | $ | & | 1.000 | & | NaN | \\ |
| $x_{31}$ | & | $ | R00830MM | $ | & | $ | x_{6},x_{9},x_{23},x_{26},x_{30},x_{31 | } |  | $ | & | 1.000 | & | NaN | \\ |
| $x_{32}$ | & | $ | R00833MM | $ | & | $ | x_{32},x_{84},x_{94},x_{127 | } | * | $ | & | 1.000 | & | 9.574 | \\ |
| $x_{33}$ | & | $ | R00851MM | $ | & | $ | x_{33},x_{54 | } | * | $ | & | 1.000 | & | 5.611 | \\ |
| $x_{34}$ | & | $ | R00927MM | $ | & | $ | x_{34},x_{77},x_{81},x_{93 | } | ** | $ | & | 1.000 | & | 16.340 | \\ |
| $x_{35}$ | & | $ | R00941MM | $ | & | $ | x_{6},x_{9},x_{23},x_{26},x_{30},x_{35 | } |  | $ | & | 1.000 | & | NaN | \\ |
| $x_{36}$ | & | $ | R00945MM | $ | & | $ | x_{6},x_{9},x_{23},x_{26},x_{30},x_{36 | } |  | $ | & | 1.000 | & | NaN | \\ |
| $x_{37}$ | & | $ | R01082MM | $ | & | $ | x_{10},x_{37},x_{107},x_{116 | } |  | $ | & | 1.000 | & | 0.048 | \\ |
| $x_{38}$ | & | $ | R01175MM | $ | & | $ | x_{38},x_{44},x_{61},x_{78},x_{87},x_{98 | } |  | $ | & | 1.000 | & | 0.076 | \\ |
| $x_{39}$ | & | $ | R01177MM | $ | & | $ | x_{8},x_{21},x_{37},x_{39},x_{44},x_{90 | } |  | $ | & | 1.000 | & | 0.076 | \\ |
| $x_{40}$ | & | $ | R01214MM | $ | & | $ | x_{40},x_{130},x_{131 | } | ** | $ | & | 1.000 | & | 9.241 | \\ |
| $x_{41}$ | & | $ | R01218MM | $ | & | $ | x_{6},x_{9},x_{23},x_{26},x_{30},x_{41 | } |  | $ | & | 1.000 | & | NaN | \\ |
| $x_{42}$ | & | $ | R01253MM | $ | & | $ | x_{6},x_{9},x_{23},x_{26},x_{30},x_{42 | } |  | $ | & | 1.000 | & | 0.000 | \\ |
| $x_{43}$ | & | $ | R01279MM | $ | & | $ | x_{4},x_{38},x_{43},x_{53},x_{85},x_{118 | } |  | $ | & | 1.000 | & | 0.076 | \\ |
| $x_{44}$ | & | $ | R01280MM | $ | & | $ | x_{44},x_{98 | } | * | $ | & | 0.998 | & | 1.466 | \\ |
| $x_{45}$ | & | $ | R01325MM | $ | & | $ | x_{38},x_{45},x_{53},x_{89},x_{98},x_{118 | } |  | $ | & | 1.000 | & | 0.048 | \\ |
| $x_{46}$ | & | $ | R01360MM | $ | & | $ | x_{46 | } |  | $ | & | 1.000 | & | 0.000 | \\ |
| $x_{47}$ | & | $ | R01361MM | $ | & | $ | x_{6},x_{9},x_{23},x_{26},x_{30},x_{47 | } |  | $ | & | 1.000 | & | 0.000 | \\ |
| $x_{48}$ | & | $ | R01624MM | $ | & | $ | x_{48},x_{121 | } | * | $ | & | 1.000 | & | 0.685 | \\ |
| $x_{49}$ | & | $ | R01626MM | $ | & | $ | x_{49 | } | * | $ | & | 0.997 | & | 4.235 | \\ |
| $x_{50}$ | & | $ | R01648MM | $ | & | $ | x_{28},x_{50 | } | ** | $ | & | 1.000 | & | 2.148 | \\ |
| $x_{51}$ | & | $ | R01655MM | $ | & | $ | x_{6},x_{9},x_{23},x_{26},x_{30},x_{51 | } |  | $ | & | 1.000 | & | NaN | \\ |
| $x_{52}$ | & | $ | R01700MM | $ | & | $ | x_{27},x_{52},x_{53},x_{85},x_{108},x_{118 | } |  | $ | & | 1.000 | & | 0.048 | \\ |
| $x_{53}$ | & | $ | R01706MM | $ | & | $ | x_{44},x_{53 | } | * | $ | & | 1.000 | & | 1.466 | \\ |
| $x_{54}$ | & | $ | R01799MM | $ | & | $ | x_{22},x_{54},x_{63 | } | * | $ | & | 1.000 | & | 5.608 | \\ |
| $x_{55}$ | & | $ | R01801MM | $ | & | $ | x_{6},x_{9},x_{23},x_{26},x_{30},x_{55 | } |  | $ | & | 1.000 | & | NaN | \\ |
| $x_{56}$ | & | $ | R01859MM | $ | & | $ | x_{31},x_{56},x_{132},x_{133 | } | * | $ | & | 1.000 | & | 16.340 | \\ |
| $x_{57}$ | & | $ | R01900MM | $ | & | $ | x_{15},x_{39},x_{53},x_{57},x_{90},x_{107 | } |  | $ | & | 1.000 | & | 0.048 | \\ |
| $x_{58}$ | & | $ | R01923MM | $ | & | $ | x_{58},x_{88},x_{90},x_{109},x_{128 | } |  | $ | & | 1.000 | & | 0.076 | \\ |
| $x_{59}$ | & | $ | R01939MM | $ | & | $ | x_{59 | } | * | $ | & | 0.999 | & | 5.480 | \\ |
| $x_{60}$ | & | $ | R01940MM | $ | & | $ | x_{12},x_{17},x_{60},x_{70},x_{74 | } |  | $ | & | 0.999 | & | 0.005 | \\ |
| $x_{61}$ | & | $ | R01975MM | $ | & | $ | x_{19},x_{21},x_{44},x_{61},x_{85},x_{134 | } |  | $ | & | 1.000 | & | 0.064 | \\ |
| $x_{62}$ | & | $ | R01978MM | $ | & | $ | x_{6},x_{9},x_{23},x_{26},x_{62 | } |  | $ | & | 0.999 | & | 0.000 | \\ |
| $x_{63}$ | & | $ | R02030MM | $ | & | $ | x_{22},x_{63 | } | ** | $ | & | 1.000 | & | 5.590 | \\ |
| $x_{64}$ | & | $ | R02161MM | $ | & | $ | x_{6},x_{9},x_{23},x_{26},x_{64 | } |  | $ | & | -0.832 | & | 0.000 | \\ |
| $x_{65}$ | & | $ | R02163MM | $ | & | $ | x_{53},x_{65},x_{85},x_{88},x_{90},x_{109 | } |  | $ | & | 1.000 | & | 0.046 | \\ |
| $x_{66}$ | & | $ | R02164MM | $ | & | $ | x_{27},x_{44},x_{66},x_{85},x_{89},x_{117 | } |  | $ | & | 1.000 | & | 0.048 | \\ |
| $x_{67}$ | & | $ | R02199MM | $ | & | $ | x_{30},x_{67},x_{133 | } | * | $ | & | 1.000 | & | 16.340 | \\ |
| $x_{68}$ | & | $ | R02241MM | $ | & | $ | x_{68},x_{129 | } | ** | $ | & | 1.000 | & | 5.608 | \\ |
| $x_{69}$ | & | $ | R02313MM | $ | & | $ | x_{69 | } | * | $ | & | 0.999 | & | 1.829 | \\ |
| $x_{70}$ | & | $ | R02487MM | $ | & | $ | x_{17},x_{60},x_{70},x_{74 | } |  | $ | & | 1.000 | & | 0.005 | \\ |
| $x_{71}$ | & | $ | R02529MM | $ | & | $ | x_{7},x_{11},x_{18},x_{71},x_{74 | } | * | $ | & | 1.000 | & | 1.622 | \\ |
| $x_{72}$ | & | $ | R02569MM | $ | & | $ | x_{2},x_{72},x_{82 | } |  | $ | & | 1.000 | & | 0.000 | \\ |
| $x_{73}$ | & | $ | R02570MM | $ | & | $ | x_{53},x_{73},x_{111},x_{114},x_{116},x_{128 | } |  | $ | & | 1.000 | & | 0.048 | \\ |
| $x_{74}$ | & | $ | R02571MM | $ | & | $ | x_{17},x_{60},x_{70},x_{74 | } |  | $ | & | 1.000 | & | 0.005 | \\ |
| $x_{75}$ | & | $ | R02661MM | $ | & | $ | x_{75 | } | * | $ | & | 1.000 | & | 9.241 | \\ |
| $x_{76}$ | & | $ | R02662MM | $ | & | $ | x_{76 | } | * | $ | & | 0.998 | & | 9.241 | \\ |
| $x_{77}$ | & | $ | R02765MM | $ | & | $ | x_{34},x_{77},x_{81},x_{93 | } | ** | $ | & | 1.000 | & | 16.340 | \\ |
| $x_{78}$ | & | $ | R03026MM | $ | & | $ | x_{4},x_{8},x_{78},x_{114 | } |  | $ | & | 1.000 | & | 0.064 | \\ |
| $x_{79}$ | & | $ | R03102MM | $ | & | $ | x_{6},x_{9},x_{23},x_{26},x_{30},x_{79 | } |  | $ | & | 1.000 | & | NaN | \\ |
| $x_{80}$ | & | $ | R03172MM | $ | & | $ | x_{52},x_{80 | } | * | $ | & | 0.996 | & | 16.340 | \\ |
| $x_{81}$ | & | $ | R03174MM | $ | & | $ | x_{34},x_{77},x_{81},x_{93 | } | ** | $ | & | 1.000 | & | 16.340 | \\ |
| $x_{82}$ | & | $ | R03270MM | $ | & | $ | x_{2},x_{72},x_{82 | } |  | $ | & | 1.000 | & | 0.000 | \\ |
| $x_{83}$ | & | $ | R03314MM | $ | & | $ | x_{6},x_{9},x_{23},x_{26},x_{83 | } |  | $ | & | 1.000 | & | 0.000 | \\ |
| $x_{84}$ | & | $ | R03381MM | $ | & | $ | x_{32},x_{84},x_{126},x_{127 | } | * | $ | & | 1.000 | & | 9.241 | \\ |
| $x_{85}$ | & | $ | R03777MM | $ | & | $ | x_{15},x_{38},x_{44},x_{85},x_{89},x_{108 | } |  | $ | & | 1.000 | & | 0.076 | \\ |
| $x_{86}$ | & | $ | R03778MM | $ | & | $ | x_{4},x_{53},x_{65},x_{86},x_{114},x_{134 | } |  | $ | & | 1.000 | & | 0.076 | \\ |
| $x_{87}$ | & | $ | R03857MM | $ | & | $ | x_{43},x_{44},x_{66},x_{87},x_{98},x_{112 | } |  | $ | & | 1.000 | & | 0.076 | \\ |
| $x_{88}$ | & | $ | R03858MM | $ | & | $ | x_{53},x_{57},x_{78},x_{88},x_{105},x_{116 | } |  | $ | & | 1.000 | & | 0.076 | \\ |
| $x_{89}$ | & | $ | R03990MM | $ | & | $ | x_{19},x_{27},x_{87},x_{89},x_{115 | } |  | $ | & | 1.000 | & | 0.076 | \\ |
| $x_{90}$ | & | $ | R03991MM | $ | & | $ | x_{37},x_{73},x_{90},x_{116 | } |  | $ | & | 1.000 | & | 0.076 | \\ |
| $x_{91}$ | & | $ | R04170MM | $ | & | $ | x_{4},x_{57},x_{65},x_{91},x_{105 | } |  | $ | & | 1.000 | & | 0.076 | \\ |
| $x_{92}$ | & | $ | R04203MM | $ | & | $ | x_{47},x_{92},x_{132},x_{133 | } | * | $ | & | 1.000 | & | 16.340 | \\ |
| $x_{93}$ | & | $ | R04204MM | $ | & | $ | x_{9},x_{34},x_{77},x_{93},x_{135 | } | * | $ | & | 0.999 | & | 16.340 | \\ |
| $x_{94}$ | & | $ | R04224MM | $ | & | $ | x_{84},x_{94},x_{126 | } | * | $ | & | 1.000 | & | 9.241 | \\ |
| $x_{95}$ | & | $ | R04355MM | $ | & | $ | x_{95 | } | * | $ | & | 1.000 | & | 0.685 | \\ |
| $x_{96}$ | & | $ | R04428MM | $ | & | $ | x_{48},x_{96 | } | * | $ | & | 1.000 | & | 0.685 | \\ |
| $x_{97}$ | & | $ | R04430MM | $ | & | $ | x_{97},x_{100 | } | * | $ | & | 1.000 | & | 0.685 | \\ |
| $x_{98}$ | & | $ | R04433MM | $ | & | $ | x_{27},x_{52},x_{98},x_{112 | } |  | $ | & | 1.000 | & | 0.074 | \\ |
| $x_{99}$ | & | $ | R04533MM | $ | & | $ | x_{97},x_{99},x_{100},x_{120 | } | * | $ | & | 1.000 | & | 0.685 | \\ |
| $x_{100}$ | & | $ | R04536MM | $ | & | $ | x_{100},x_{123 | } | * | $ | & | 1.000 | & | 0.685 | \\ |
| $x_{101}$ | & | $ | R04537MM | $ | & | $ | x_{96},x_{101 | } | * | $ | & | 1.000 | & | 0.685 | \\ |
| $x_{102}$ | & | $ | R04543MM | $ | & | $ | x_{102},x_{124},x_{125 | } | * | $ | & | 1.000 | & | 1.466 | \\ |
| $x_{103}$ | & | $ | R04544MM | $ | & | $ | x_{103},x_{124 | } | ** | $ | & | 1.000 | & | 1.466 | \\ |
| $x_{104}$ | & | $ | R04737MM | $ | & | $ | x_{44},x_{45},x_{61},x_{104},x_{107},x_{118 | } |  | $ | & | 1.000 | & | 0.076 | \\ |
| $x_{105}$ | & | $ | R04738MM | $ | & | $ | x_{10},x_{39},x_{44},x_{73},x_{91},x_{105 | } |  | $ | & | 1.000 | & | 0.076 | \\ |
| $x_{106}$ | & | $ | R04739MM | $ | & | $ | x_{53},x_{61},x_{89},x_{106},x_{118},x_{134 | } |  | $ | & | 1.000 | & | 0.076 | \\ |
| $x_{107}$ | & | $ | R04740MM | $ | & | $ | x_{13},x_{53},x_{65},x_{86},x_{90},x_{107 | } |  | $ | & | 1.000 | & | 0.076 | \\ |
| $x_{108}$ | & | $ | R04741MM | $ | & | $ | x_{38},x_{53},x_{89},x_{98},x_{104},x_{108 | } |  | $ | & | 1.000 | & | 0.076 | \\ |
| $x_{109}$ | & | $ | R04742MM | $ | & | $ | x_{39},x_{44},x_{65},x_{90},x_{109},x_{113 | } |  | $ | & | 1.000 | & | 0.076 | \\ |
| $x_{110}$ | & | $ | R04743MM | $ | & | $ | x_{44},x_{61},x_{89},x_{98},x_{106},x_{110 | } |  | $ | & | 1.000 | & | 0.076 | \\ |
| $x_{111}$ | & | $ | R04744MM | $ | & | $ | x_{10},x_{44},x_{57},x_{86},x_{91},x_{111 | } |  | $ | & | 1.000 | & | 0.076 | \\ |
| $x_{112}$ | & | $ | R04745MM | $ | & | $ | x_{52},x_{53},x_{85},x_{110},x_{112},x_{118 | } |  | $ | & | 1.000 | & | 0.076 | \\ |
| $x_{113}$ | & | $ | R04746MM | $ | & | $ | x_{44},x_{58},x_{78},x_{90},x_{105},x_{113 | } |  | $ | & | 1.000 | & | 0.076 | \\ |
| $x_{114}$ | & | $ | R04747MM | $ | & | $ | x_{53},x_{78},x_{89},x_{105},x_{113},x_{114 | } |  | $ | & | 1.000 | & | 0.076 | \\ |
| $x_{115}$ | & | $ | R04748MM | $ | & | $ | x_{52},x_{53},x_{88},x_{108},x_{115},x_{118 | } |  | $ | & | 1.000 | & | 0.076 | \\ |
| $x_{116}$ | & | $ | R04749MM | $ | & | $ | x_{13},x_{16},x_{53},x_{66},x_{113},x_{116 | } |  | $ | & | 1.000 | & | 0.076 | \\ |
| $x_{117}$ | & | $ | R04751MM | $ | & | $ | x_{66},x_{80},x_{87},x_{98},x_{117},x_{118 | } |  | $ | & | 1.000 | & | 0.076 | \\ |
| $x_{118}$ | & | $ | R04754MM | $ | & | $ | x_{19},x_{45},x_{53},x_{85},x_{89},x_{118 | } |  | $ | & | 1.000 | & | 0.076 | \\ |
| $x_{119}$ | & | $ | R04952MM | $ | & | $ | x_{9},x_{41},x_{79},x_{119 | } | * | $ | & | 0.998 | & | 0.685 | \\ |
| $x_{120}$ | & | $ | R04953MM | $ | & | $ | x_{100},x_{120},x_{122 | } | * | $ | & | 1.000 | & | 0.685 | \\ |
| $x_{121}$ | & | $ | R04954MM | $ | & | $ | x_{96},x_{101},x_{121 | } | * | $ | & | 1.000 | & | 0.685 | \\ |
| $x_{122}$ | & | $ | R04956MM | $ | & | $ | x_{120},x_{122 | } | * | $ | & | 1.000 | & | 0.685 | \\ |
| $x_{123}$ | & | $ | R04959MM | $ | & | $ | x_{97},x_{100},x_{123 | } | * | $ | & | 1.000 | & | 0.685 | \\ |
| $x_{124}$ | & | $ | R04968MM | $ | & | $ | x_{103},x_{124 | } | ** | $ | & | 1.000 | & | 1.466 | \\ |
| $x_{125}$ | & | $ | R04970MM | $ | & | $ | x_{102},x_{103},x_{125 | } | * | $ | & | 1.000 | & | 1.466 | \\ |
| $x_{126}$ | & | $ | R05064MM | $ | & | $ | x_{32},x_{126 | } | * | $ | & | 1.000 | & | 9.241 | \\ |
| $x_{127}$ | & | $ | R05066MM | $ | & | $ | x_{94},x_{127 | } | * | $ | & | 1.000 | & | 9.241 | \\ |
| $x_{128}$ | & | $ | R07162MM | $ | & | $ | x_{10},x_{16},x_{88},x_{128 | } |  | $ | & | 1.000 | & | 0.046 | \\ |
| $x_{129}$ | & | $ | R07390MM | $ | & | $ | x_{68},x_{129 | } | ** | $ | & | 1.000 | & | 5.590 | \\ |
| $x_{130}$ | & | $ | R07599MM | $ | & | $ | x_{130},x_{131 | } | * | $ | & | 1.000 | & | 9.241 | \\ |
| $x_{131}$ | & | $ | R07600MM | $ | & | $ | x_{40},x_{130},x_{131 | } | ** | $ | & | 1.000 | & | 9.241 | \\ |
| $x_{132}$ | & | $ | R07603MM | $ | & | $ | x_{56},x_{67},x_{92},x_{132},x_{133 | } | * | $ | & | 1.000 | & | 16.340 | \\ |
| $x_{133}$ | & | $ | R07604MM | $ | & | $ | x_{56},x_{92},x_{132},x_{133 | } | * | $ | & | 1.000 | & | 16.340 | \\ |
| $x_{134}$ | & | $ | R07618MM | $ | & | $ | x_{53},x_{61},x_{85},x_{108},x_{115},x_{134 | } |  | $ | & | 1.000 | & | 0.039 | \\ |
| $x_{135}$ | & | $ | R08157MM | $ | & | $ | x_{6},x_{9},x_{23},x_{26},x_{30},x_{135 | } |  | $ | & | 1.000 | & | 0.000 | \\ |
