# Supplementary material for: Multi-Target Analysis and Design of Mitochondrial Metabolism
Source: PLoS One. 2015 Sep 16;10(9):e0133825. doi: 10.1371/journal.pone.0133825 (PMC4574446; doi:10.1371/journal.pone.0133825)
Supplement: S1 Code — (ZIP) [file pone.0133825.s004.zip › source code for publication/Identifiability Analysis on monogenic diseases/MOTA/mota-manual.pdf]

Quickstart to the MOTA-Software

Stefan Hengl
hengl@fdm.uni-freiburg.de

November 28, 2007

The *Mean Optimal Transformation Approach* (MOTA) was developed for identifiability analysis of nonlinear models [1]. Since non-identifiable parameters are functionally related with other non-identifiable parameters, we can map the problem of an analytical analysis of the model structure onto the problem of detecting functional relations of parameter estimates.

Installation

MOTA should run on Windows, Linux, and Mac OS. However, it was mainly tested on Windows. Mex-files for Linux and Mac OS were kindly provided by users. To install, just copy all files in a directory of your choice. You have to set the path for this directory in MATLAB. With MATLAB open, select File from the menu-bar: *File > Set Path.... > Add with Subfolders*. Now browse for the directory, finished.

Requirements

The mean optimal transformation approach (MOTA) requires a (n x p) Matrix K as input, where n denotes the number of estimates for each of the p parameters. K is ,e.g. the result of n fits of a model with p parameters to data. Any tool capable

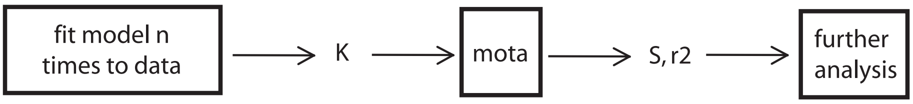

of fitting your model to data will work, just use your favorite. It is crucial that all estimates which are taken to populate the matrix K have a comparable χ^2-value; this is just another way to define non-identifiability. We recommend our developed MATLAB multi-experiment fitting tool PottersWheel to produce a proper Matrix K. It can be downloaded at www.potterswheel.de. An interface-function *motaGetK.p* is included in the MOTA-package (see below).

The number of fits is somehow arbitrary, but experience has shown that $n > 100$ is a good choice. Note, it depends on the functional relation and the data how many estimates are actually required. A straight line as the functional relation of two parameters certainly requires far less estimates than a fast oscillating sine.

Quickstart

Once a proper Matrix K has been created, our algorithm can be called from the *command window* in MATLAB like follows:

$$>> [S, r2] = mota(K)$$

The matrix S contains ones and zeros indicating functional relations between the parameters. The order corresponds to the order given by K. The matrix r2 contains for each parameter the multiple r^2 calculated every time a new parameter has been added during the algorithm. In order to investigate the results, combine $S, r2$, and K to an object of class *motaR*:

$$>> h = motaR(K, S, r2)$$

Following commands are available to investigate the output of *MOTA*:

>> *print(h)* lists S and r2 in tabular form with additional information (see Figure 1)

>> *plot(h,ix)* produces scatter-plots and ACE-plots of a detected functional relation.

Extended Options

MOTA

[S,r2]=mota(K,IO,threshold1,threshold2,threshold3,sampleSize,numOfBootsamp, MAXNOP).

[IO]

0: no text output during the call (default)
1: text output in command line during the call. Loop procedure is displayed
2: calculation of multiple r2 is displayed as well

[thresholdX]

X=1: threshold1=0.01(default). Determines the value of the test-function **below** which no funtional relation between the parameters in the current set is assigned.
X=2: threshold2=0.07(default). Determines the value of the test-function **above** which a funtional relation between the parameters in the current set is assigned.

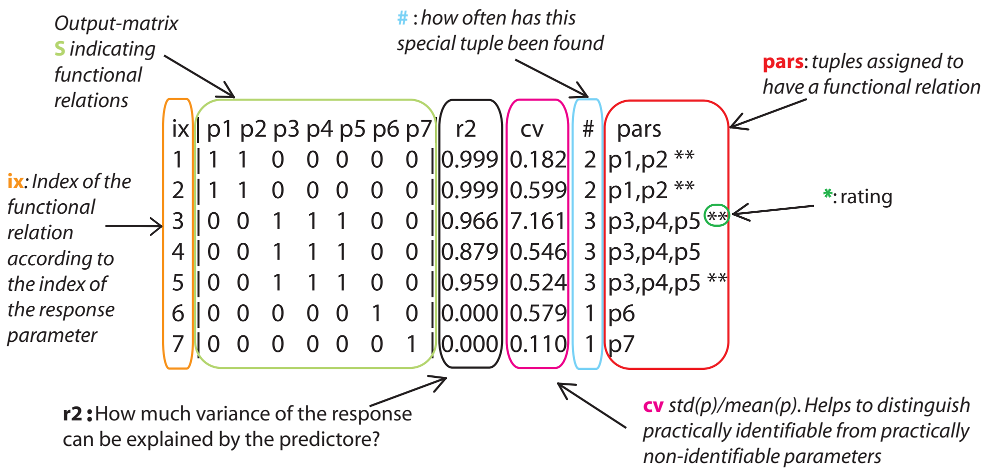

Figure 1: Output of the command >>*print(h) for an arbitrary example*

X=3: threshold3=0.08(default). Determines the value of the test-function **above** which additional parameters are accepted even if the test-function did not improve with the recently added parameter. This threshold is not neccessary for the functionality of the algorithm, but it slightly improves the performance.

[sampleSize]

Size of the bootstrap samples drawn from the matrix K (default=[N/2]).

[numOfBootsamp]

Number of the bootstrap samples drawn from the matrix K (default=35).

[MAXNOP]

Maximal number of parameters which are allowed to comprise a functional relation. The default value equals, of course, the number of model parameters. However, a lower value of MAXNOP reduces the computational effort tremendously and saves time. So, if the researcher is only interested in functional relations of for exmample 3 parameters, he/she could force the algorithm to do so by setting MAXNOP appropriately.

print
print(h,'options',exportToLaTeXCode=0)
sorts the table according to options. Allowed values for *options* are (see Figure 1)

org (default)
cv
#

exportToLaTeXCode is a boolean variable indicating whether the table should be printed in a .txt file with LaTeX syntax. If set to *exportToLaTeXCode=1* (not default) , the second argument is obligatory.

plot
plot(h,ix,'options')
plots the functional relation of the row *ix*. Allowed values for *options* are

scatter: scatter plots
ace: aceplots

Currently scatter plots can only be produced for two and three parameter relations. The option *ace* is not restricted.

Test-Examples

The file *motaExamples* contains a few examples of nonlinearly related parameters. For every sample, a matrix K, equally distributed on an interval I, is drawn and functional relations are introduced as indicated. Real data is always corrupted with observational noise, therefore we add Gaussian noise to the left hand side terms. The function *motaExamples(j)* returns the matrix K for the example j.

$$>> K = motaExamples(j)$$

In order to apply MOTA to the test-example, type

$$>> [S, r2] = mota(K)$$

PottersWheel Interface

An interface-function *motaGetK.p* for PottersWheel is included in the MOTA-package. After a fitting-sequence has been conducted with PottersWheel, type

$$>> K = motaGetK(percentage, IO = 0)$$

percentage denotes the percentage of the best fits taken to populate the matrix K. A MATLAB-figure pops up, showing the distribution of χ^2-values (if IO is set to $IO = 1$, the figure is suppressed). Accepted fits are encircled in red. All accepted fits should have comparable χ^2-values.
MOTA has also been implemented directly in PottersWheel as PlugIn. Thanks to Thomas Maiwald, MOTA may now easily be accessed within the PottersWheel GUI (see www.potterswheel.de).

Recommendations

Here we list some of our experiences which should help to benefit from the result. We suggest to rank the functional relation according to following criteria:

1. The more often a functional relation has been found (#) the better.
2. The more variance of the response can be explained by the predictors (r^2), the larger the effect of the fixation of parameters on the standard deviations of the fitted parameters. Values of $r^2 > 0.9$ are very good.
3. The MOT-approach is scale free, i.e. functional relations are detected regardless of the relative variation of the parameters. It may happen, that even practically identifiable parameters comprise functional relations. We suggest not to take functional relations of practical identifiable parameters into account. The decision which parameters are practically identifiable depends on the observational noise.

We included these recommendations in form the asterisks ($*$) attached to the table-output of *(*print). Functional relations with $r^2 > 0.9$ and $cv > 0.1$ are given one ($*$). If, additionally, the functional relation has been found more than once, another ($*$) is assigned.

Remarks

In principle, a functional relation consisting of k parameters is tested k times during the algorithm, since all parameters are once taken as response variable. However, for real models and real data it is unlikely that all related parameters are detected for every response variable. It depends on the contribution strength of a predictor to the response if a parameter is detected or not. This causes an output-Matrix S, which does not look uniform anymore.

References

[1] S. Hengl, C. Kreutz, J. Timmer, and T. Maiwald. Data-based identifiability analysis of non-linear dynamical models. *Bioinformatics*, 23(19):2612–2618, Oct 2007.
