# Supplementary material for: Multi-Target Analysis and Design of Mitochondrial Metabolism
Source: PLoS One. 2015 Sep 16;10(9):e0133825. doi: 10.1371/journal.pone.0133825 (PMC4574446; doi:10.1371/journal.pone.0133825)
Supplement: S1 Code — (ZIP) [file pone.0133825.s004.zip › source code for publication/Identifiability Analysis on monogenic diseases/Succinate dehydrogenase deficiency/tabella latex - inflammation.docx]

| $x_1$ | & | $ | R00004MM | $ | & | $ | x_{1},x_{44},x_{53},x_{103},x_{124 | } | ** | $ | & | 0.998 | & | 13.281 | \\ |
| --- | --- | --- | --- | --- | --- | --- | --- | --- | --- | --- | --- | --- | --- | --- | --- |
| $x_2$ | & | $ | R00014MM | $ | & | $ | x_{2},x_{33},x_{41},x_{82 | } | * | $ | & | 1.000 | & | 1.969 | \\ |
| $x_3$ | & | $ | R00081MM | $ | & | $ | x_{3},x_{4},x_{10},x_{128 | } | * | $ | & | 1.000 | & | 0.116 | \\ |
| $x_4$ | & | $ | R00086MM | $ | & | $ | x_{4},x_{15},x_{37},x_{115 | } | * | $ | & | 1.000 | & | 0.113 | \\ |
| $x_5$ | & | $ | R00127MM | $ | & | $ | x_{5 | } | * | $ | & | 0.984 | & | 11.428 | \\ |
| $x_6$ | & | $ | R00157MM | $ | & | $ | x_{6 | } | * | $ | & | 0.999 | & | 11.428 | \\ |
| $x_7$ | & | $ | R00205MM | $ | & | $ | x_{7 | } |  | $ | & | 0.973 | & | 0.000 | \\ |
| $x_8$ | & | $ | R00238MM | $ | & | $ | x_{8},x_{39},x_{43},x_{90},x_{91 | } | * | $ | & | 1.000 | & | 0.199 | \\ |
| $x_9$ | & | $ | R00243MM | $ | & | $ | x_{9},x_{22},x_{26},x_{29},x_{30},x_{31 | } |  | $ | & | 1.000 | & | NaN | \\ |
| $x_{10}$ | & | $ | R00245MM | $ | & | $ | x_{3},x_{10},x_{106},x_{128 | } |  | $ | & | 1.000 | & | 0.032 | \\ |
| $x_{11}$ | & | $ | R00256MM | $ | & | $ | x_{11 | } |  | $ | & | 0.995 | & | 0.000 | \\ |
| $x_{12}$ | & | $ | R00258MM | $ | & | $ | x_{12 | } |  | $ | & | 0.000 | & | 0.000 | \\ |
| $x_{13}$ | & | $ | R00275MM | $ | & | $ | x_{4},x_{13},x_{43},x_{64},x_{112 | } | * | $ | & | 1.000 | & | 0.104 | \\ |
| $x_{14}$ | & | $ | R00330MM | $ | & | $ | x_{14 | } | * | $ | & | 1.000 | & | 0.250 | \\ |
| $x_{15}$ | & | $ | R00342MM | $ | & | $ | x_{15},x_{43},x_{104},x_{110},x_{115 | } |  | $ | & | 1.000 | & | 0.082 | \\ |
| $x_{16}$ | & | $ | R00351MM | $ | & | $ | x_{16},x_{39},x_{57},x_{58},x_{113 | } | * | $ | & | 1.000 | & | 0.119 | \\ |
| $x_{17}$ | & | $ | R00355MM | $ | & | $ | x_{17},x_{60 | } |  | $ | & | 0.999 | & | 0.000 | \\ |
| $x_{18}$ | & | $ | R00371MM | $ | & | $ | x_{18 | } |  | $ | & | 0.997 | & | 0.000 | \\ |
| $x_{19}$ | & | $ | R00388MM | $ | & | $ | x_{19},x_{37},x_{52},x_{109},x_{116 | } |  | $ | & | 1.000 | & | 0.048 | \\ |
| $x_{20}$ | & | $ | R00430MM | $ | & | $ | x_{20},x_{106},x_{109},x_{118},x_{134 | } | * | $ | & | 1.000 | & | 0.251 | \\ |
| $x_{21}$ | & | $ | R00432MM | $ | & | $ | x_{21},x_{39},x_{57},x_{91},x_{111 | } | * | $ | & | 1.000 | & | 0.119 | \\ |
| $x_{22}$ | & | $ | R00512MM | $ | & | $ | x_{9},x_{22},x_{26},x_{29},x_{30},x_{31 | } |  | $ | & | 1.000 | & | NaN | \\ |
| $x_{23}$ | & | $ | R00551MM | $ | & | $ | x_{23},x_{31},x_{40 | } |  | $ | & | 0.999 | & | 0.000 | \\ |
| $x_{24}$ | & | $ | R00572MM | $ | & | $ | x_{20},x_{24},x_{45},x_{88 | } | * | $ | & | 1.000 | & | 0.251 | \\ |
| $x_{25}$ | & | $ | R00667MM | $ | & | $ | x_{25},x_{33},x_{60 | } |  | $ | & | 0.999 | & | 0.000 | \\ |
| $x_{26}$ | & | $ | R00705MM | $ | & | $ | x_{9},x_{22},x_{26},x_{29},x_{30},x_{31 | } |  | $ | & | 1.000 | & | NaN | \\ |
| $x_{27}$ | & | $ | R00709MM | $ | & | $ | x_{4},x_{27},x_{87},x_{106 | } | * | $ | & | 1.000 | & | 0.119 | \\ |
| $x_{28}$ | & | $ | R00713MM | $ | & | $ | x_{28},x_{50 | } | ** | $ | & | 1.000 | & | 3.994 | \\ |
| $x_{29}$ | & | $ | R00716MM | $ | & | $ | x_{9},x_{22},x_{26},x_{29},x_{30},x_{31 | } |  | $ | & | 1.000 | & | 0.000 | \\ |
| $x_{30}$ | & | $ | R00740MM | $ | & | $ | x_{9},x_{22},x_{26},x_{29},x_{30},x_{31 | } |  | $ | & | 1.000 | & | NaN | \\ |
| $x_{31}$ | & | $ | R00830MM | $ | & | $ | x_{9},x_{22},x_{26},x_{29},x_{30},x_{31 | } |  | $ | & | 1.000 | & | NaN | \\ |
| $x_{32}$ | & | $ | R00833MM | $ | & | $ | x_{9},x_{22},x_{26},x_{29},x_{32 | } |  | $ | & | 0.999 | & | 0.000 | \\ |
| $x_{33}$ | & | $ | R00851MM | $ | & | $ | x_{9},x_{22},x_{26},x_{29},x_{30},x_{33 | } |  | $ | & | 1.000 | & | NaN | \\ |
| $x_{34}$ | & | $ | R00927MM | $ | & | $ | x_{34},x_{81 | } | * | $ | & | 1.000 | & | 6.807 | \\ |
| $x_{35}$ | & | $ | R00941MM | $ | & | $ | x_{9},x_{22},x_{26},x_{29},x_{30},x_{35 | } |  | $ | & | 1.000 | & | 0.000 | \\ |
| $x_{36}$ | & | $ | R00945MM | $ | & | $ | x_{9},x_{22},x_{26},x_{29},x_{30},x_{36 | } |  | $ | & | 1.000 | & | 0.000 | \\ |
| $x_{37}$ | & | $ | R01082MM | $ | & | $ | x_{19},x_{37},x_{57},x_{116 | } | * | $ | & | 1.000 | & | 0.123 | \\ |
| $x_{38}$ | & | $ | R01175MM | $ | & | $ | x_{38},x_{64},x_{85},x_{110},x_{112 | } | * | $ | & | 1.000 | & | 0.217 | \\ |
| $x_{39}$ | & | $ | R01177MM | $ | & | $ | x_{38},x_{39},x_{98},x_{107},x_{109 | } | * | $ | & | 1.000 | & | 0.217 | \\ |
| $x_{40}$ | & | $ | R01214MM | $ | & | $ | x_{9},x_{22},x_{26},x_{29},x_{30},x_{40 | } |  | $ | & | 1.000 | & | 0.000 | \\ |
| $x_{41}$ | & | $ | R01218MM | $ | & | $ | x_{9},x_{22},x_{26},x_{29},x_{30},x_{41 | } |  | $ | & | 1.000 | & | 0.000 | \\ |
| $x_{42}$ | & | $ | R01253MM | $ | & | $ | x_{9},x_{22},x_{26},x_{29},x_{30},x_{42 | } |  | $ | & | 1.000 | & | 0.000 | \\ |
| $x_{43}$ | & | $ | R01279MM | $ | & | $ | x_{3},x_{43},x_{66},x_{108 | } | * | $ | & | 1.000 | & | 0.217 | \\ |
| $x_{44}$ | & | $ | R01280MM | $ | & | $ | x_{1},x_{44},x_{53},x_{103 | } | * | $ | & | 0.999 | & | 13.325 | \\ |
| $x_{45}$ | & | $ | R01325MM | $ | & | $ | x_{19},x_{43},x_{45},x_{61},x_{87 | } | * | $ | & | 1.000 | & | 0.119 | \\ |
| $x_{46}$ | & | $ | R01360MM | $ | & | $ | x_{46},x_{47},x_{62 | } | ** | $ | & | 1.000 | & | 1.614 | \\ |
| $x_{47}$ | & | $ | R01361MM | $ | & | $ | x_{46},x_{47},x_{62 | } | ** | $ | & | 1.000 | & | 1.618 | \\ |
| $x_{48}$ | & | $ | R01624MM | $ | & | $ | x_{48},x_{95 | } | * | $ | & | 0.998 | & | 7.263 | \\ |
| $x_{49}$ | & | $ | R01626MM | $ | & | $ | x_{49},x_{122 | } | * | $ | & | 0.990 | & | 3.750 | \\ |
| $x_{50}$ | & | $ | R01648MM | $ | & | $ | x_{28},x_{50 | } | ** | $ | & | 1.000 | & | 3.994 | \\ |
| $x_{51}$ | & | $ | R01655MM | $ | & | $ | x_{9},x_{22},x_{26},x_{29},x_{30},x_{51 | } |  | $ | & | 1.000 | & | 0.000 | \\ |
| $x_{52}$ | & | $ | R01700MM | $ | & | $ | x_{3},x_{52},x_{64},x_{108 | } | * | $ | & | 1.000 | & | 0.119 | \\ |
| $x_{53}$ | & | $ | R01706MM | $ | & | $ | x_{1},x_{44},x_{53},x_{103},x_{124 | } | ** | $ | & | 0.998 | & | 13.285 | \\ |
| $x_{54}$ | & | $ | R01799MM | $ | & | $ | x_{9},x_{22},x_{26},x_{29},x_{30},x_{54 | } |  | $ | & | 1.000 | & | NaN | \\ |
| $x_{55}$ | & | $ | R01801MM | $ | & | $ | x_{9},x_{22},x_{26},x_{29},x_{30},x_{55 | } |  | $ | & | 1.000 | & | NaN | \\ |
| $x_{56}$ | & | $ | R01859MM | $ | & | $ | x_{9},x_{22},x_{26},x_{29},x_{30},x_{56 | } |  | $ | & | 1.000 | & | NaN | \\ |
| $x_{57}$ | & | $ | R01900MM | $ | & | $ | x_{39},x_{57},x_{90},x_{109},x_{116 | } | * | $ | & | 1.000 | & | 0.119 | \\ |
| $x_{58}$ | & | $ | R01923MM | $ | & | $ | x_{16},x_{37},x_{38},x_{58},x_{90 | } | * | $ | & | 1.000 | & | 0.217 | \\ |
| $x_{59}$ | & | $ | R01939MM | $ | & | $ | x_{26},x_{51},x_{59},x_{135 | } |  | $ | & | 0.998 | & | 0.000 | \\ |
| $x_{60}$ | & | $ | R01940MM | $ | & | $ | x_{9},x_{22},x_{26},x_{29},x_{30},x_{60 | } |  | $ | & | 1.000 | & | 0.000 | \\ |
| $x_{61}$ | & | $ | R01975MM | $ | & | $ | x_{61},x_{85},x_{106},x_{110},x_{134 | } | * | $ | & | 1.000 | & | 0.199 | \\ |
| $x_{62}$ | & | $ | R01978MM | $ | & | $ | x_{46},x_{47},x_{62 | } | ** | $ | & | 1.000 | & | 1.614 | \\ |
| $x_{63}$ | & | $ | R02030MM | $ | & | $ | x_{9},x_{22},x_{26},x_{29},x_{30},x_{63 | } |  | $ | & | 1.000 | & | NaN | \\ |
| $x_{64}$ | & | $ | R02161MM | $ | & | $ | x_{10},x_{64},x_{89},x_{104 | } | * | $ | & | 1.000 | & | 0.116 | \\ |
| $x_{65}$ | & | $ | R02163MM | $ | & | $ | x_{27},x_{65},x_{90},x_{112},x_{128 | } | * | $ | & | 1.000 | & | 0.104 | \\ |
| $x_{66}$ | & | $ | R02164MM | $ | & | $ | x_{13},x_{27},x_{66},x_{114},x_{134 | } | * | $ | & | 1.000 | & | 0.123 | \\ |
| $x_{67}$ | & | $ | R02199MM | $ | & | $ | x_{67},x_{132 | } | * | $ | & | 1.000 | & | 6.807 | \\ |
| $x_{68}$ | & | $ | R02241MM | $ | & | $ | x_{9},x_{22},x_{26},x_{29},x_{30},x_{68 | } |  | $ | & | 1.000 | & | NaN | \\ |
| $x_{69}$ | & | $ | R02313MM | $ | & | $ | x_{9},x_{22},x_{26},x_{29},x_{30},x_{69 | } |  | $ | & | 1.000 | & | 0.000 | \\ |
| $x_{70}$ | & | $ | R02487MM | $ | & | $ | x_{51},x_{70 | } |  | $ | & | 1.000 | & | 0.000 | \\ |
| $x_{71}$ | & | $ | R02529MM | $ | & | $ | x_{71 | } |  | $ | & | 0.989 | & | 0.000 | \\ |
| $x_{72}$ | & | $ | R02569MM | $ | & | $ | x_{72 | } |  | $ | & | 0.881 | & | 0.000 | \\ |
| $x_{73}$ | & | $ | R02570MM | $ | & | $ | x_{37},x_{73},x_{86},x_{105},x_{112 | } | * | $ | & | 1.000 | & | 0.119 | \\ |
| $x_{74}$ | & | $ | R02571MM | $ | & | $ | x_{9},x_{22},x_{26},x_{29},x_{74 | } |  | $ | & | 1.000 | & | 0.000 | \\ |
| $x_{75}$ | & | $ | R02661MM | $ | & | $ | x_{31},x_{40},x_{75 | } |  | $ | & | 1.000 | & | 0.000 | \\ |
| $x_{76}$ | & | $ | R02662MM | $ | & | $ | x_{9},x_{22},x_{26},x_{29},x_{76 | } |  | $ | & | 1.000 | & | 0.000 | \\ |
| $x_{77}$ | & | $ | R02765MM | $ | & | $ | x_{9},x_{22},x_{26},x_{29},x_{30},x_{77 | } |  | $ | & | 1.000 | & | NaN | \\ |
| $x_{78}$ | & | $ | R03026MM | $ | & | $ | x_{19},x_{58},x_{78},x_{111},x_{116 | } | * | $ | & | 1.000 | & | 0.199 | \\ |
| $x_{79}$ | & | $ | R03102MM | $ | & | $ | x_{9},x_{22},x_{26},x_{29},x_{79 | } |  | $ | & | 0.996 | & | 0.000 | \\ |
| $x_{80}$ | & | $ | R03172MM | $ | & | $ | x_{80 | } | * | $ | & | 0.994 | & | 131.307 | \\ |
| $x_{81}$ | & | $ | R03174MM | $ | & | $ | x_{34},x_{81},x_{93 | } | * | $ | & | 1.000 | & | 6.807 | \\ |
| $x_{82}$ | & | $ | R03270MM | $ | & | $ | x_{2},x_{9},x_{42},x_{55},x_{82 | } | * | $ | & | 1.000 | & | 1.969 | \\ |
| $x_{83}$ | & | $ | R03314MM | $ | & | $ | x_{9},x_{22},x_{26},x_{29},x_{30},x_{83 | } |  | $ | & | 1.000 | & | 0.000 | \\ |
| $x_{84}$ | & | $ | R03381MM | $ | & | $ | x_{51},x_{83},x_{84 | } |  | $ | & | 1.000 | & | 0.000 | \\ |
| $x_{85}$ | & | $ | R03777MM | $ | & | $ | x_{64},x_{66},x_{85},x_{118 | } | * | $ | & | 1.000 | & | 0.217 | \\ |
| $x_{86}$ | & | $ | R03778MM | $ | & | $ | x_{64},x_{73},x_{86},x_{109 | } | * | $ | & | 1.000 | & | 0.217 | \\ |
| $x_{87}$ | & | $ | R03857MM | $ | & | $ | x_{65},x_{85},x_{87},x_{110 | } | * | $ | & | 1.000 | & | 0.217 | \\ |
| $x_{88}$ | & | $ | R03858MM | $ | & | $ | x_{39},x_{57},x_{78},x_{88 | } | * | $ | & | 1.000 | & | 0.217 | \\ |
| $x_{89}$ | & | $ | R03990MM | $ | & | $ | x_{3},x_{57},x_{89},x_{98},x_{118 | } | * | $ | & | 1.000 | & | 0.217 | \\ |
| $x_{90}$ | & | $ | R03991MM | $ | & | $ | x_{8},x_{39},x_{57},x_{90 | } | * | $ | & | 1.000 | & | 0.217 | \\ |
| $x_{91}$ | & | $ | R04170MM | $ | & | $ | x_{37},x_{58},x_{78},x_{91 | } | * | $ | & | 1.000 | & | 0.217 | \\ |
| $x_{92}$ | & | $ | R04203MM | $ | & | $ | x_{67},x_{92},x_{133 | } | * | $ | & | 1.000 | & | 6.807 | \\ |
| $x_{93}$ | & | $ | R04204MM | $ | & | $ | x_{81},x_{93 | } | * | $ | & | 1.000 | & | 6.807 | \\ |
| $x_{94}$ | & | $ | R04224MM | $ | & | $ | x_{94},x_{129 | } |  | $ | & | 1.000 | & | 0.000 | \\ |
| $x_{95}$ | & | $ | R04355MM | $ | & | $ | x_{95},x_{101},x_{123 | } | * | $ | & | 0.998 | & | 0.521 | \\ |
| $x_{96}$ | & | $ | R04428MM | $ | & | $ | x_{96},x_{101},x_{121},x_{123 | } | * | $ | & | 1.000 | & | 7.370 | \\ |
| $x_{97}$ | & | $ | R04430MM | $ | & | $ | x_{97},x_{99},x_{101},x_{122},x_{123 | } | * | $ | & | 0.998 | & | 7.370 | \\ |
| $x_{98}$ | & | $ | R04433MM | $ | & | $ | x_{98},x_{108},x_{112},x_{117},x_{118 | } | * | $ | & | 1.000 | & | 0.213 | \\ |
| $x_{99}$ | & | $ | R04533MM | $ | & | $ | x_{49},x_{97},x_{99},x_{119},x_{122},x_{123 | } | * | $ | & | 0.997 | & | 7.370 | \\ |
| $x_{100}$ | & | $ | R04536MM | $ | & | $ | x_{100},x_{101},x_{120},x_{123 | } | * | $ | & | 1.000 | & | 7.380 | \\ |
| $x_{101}$ | & | $ | R04537MM | $ | & | $ | x_{96},x_{101},x_{120},x_{121 | } | * | $ | & | 1.000 | & | 7.380 | \\ |
| $x_{102}$ | & | $ | R04543MM | $ | & | $ | x_{100},x_{102},x_{103},x_{124},x_{125 | } | * | $ | & | 1.000 | & | 13.300 | \\ |
| $x_{103}$ | & | $ | R04544MM | $ | & | $ | x_{44},x_{53},x_{103},x_{124},x_{125 | } | * | $ | & | 0.998 | & | 13.300 | \\ |
| $x_{104}$ | & | $ | R04737MM | $ | & | $ | x_{85},x_{89},x_{104},x_{106},x_{110 | } | * | $ | & | 1.000 | & | 0.217 | \\ |
| $x_{105}$ | & | $ | R04738MM | $ | & | $ | x_{8},x_{90},x_{105},x_{110 | } | * | $ | & | 1.000 | & | 0.217 | \\ |
| $x_{106}$ | & | $ | R04739MM | $ | & | $ | x_{73},x_{89},x_{98},x_{106},x_{112 | } | * | $ | & | 1.000 | & | 0.217 | \\ |
| $x_{107}$ | & | $ | R04740MM | $ | & | $ | x_{91},x_{105},x_{106},x_{107},x_{116 | } | * | $ | & | 1.000 | & | 0.217 | \\ |
| $x_{108}$ | & | $ | R04741MM | $ | & | $ | x_{39},x_{108},x_{112},x_{116},x_{117 | } | * | $ | & | 1.000 | & | 0.217 | \\ |
| $x_{109}$ | & | $ | R04742MM | $ | & | $ | x_{91},x_{109},x_{110},x_{114},x_{117 | } | * | $ | & | 1.000 | & | 0.217 | \\ |
| $x_{110}$ | & | $ | R04743MM | $ | & | $ | x_{4},x_{87},x_{110},x_{134 | } | * | $ | & | 1.000 | & | 0.217 | \\ |
| $x_{111}$ | & | $ | R04744MM | $ | & | $ | x_{78},x_{110},x_{111},x_{113},x_{116 | } | * | $ | & | 1.000 | & | 0.217 | \\ |
| $x_{112}$ | & | $ | R04745MM | $ | & | $ | x_{65},x_{66},x_{98},x_{112 | } | * | $ | & | 1.000 | & | 0.217 | \\ |
| $x_{113}$ | & | $ | R04746MM | $ | & | $ | x_{16},x_{37},x_{57},x_{113 | } | * | $ | & | 1.000 | & | 0.217 | \\ |
| $x_{114}$ | & | $ | R04747MM | $ | & | $ | x_{43},x_{91},x_{107},x_{114 | } | * | $ | & | 1.000 | & | 0.217 | \\ |
| $x_{115}$ | & | $ | R04748MM | $ | & | $ | x_{3},x_{27},x_{38},x_{115 | } | * | $ | & | 1.000 | & | 0.217 | \\ |
| $x_{116}$ | & | $ | R04749MM | $ | & | $ | x_{73},x_{86},x_{113},x_{115},x_{116 | } | * | $ | & | 1.000 | & | 0.217 | \\ |
| $x_{117}$ | & | $ | R04751MM | $ | & | $ | x_{38},x_{45},x_{98},x_{115},x_{117 | } | * | $ | & | 1.000 | & | 0.217 | \\ |
| $x_{118}$ | & | $ | R04754MM | $ | & | $ | x_{3},x_{115},x_{118},x_{134 | } | * | $ | & | 1.000 | & | 0.217 | \\ |
| $x_{119}$ | & | $ | R04952MM | $ | & | $ | x_{96},x_{101},x_{119},x_{121 | } | ** | $ | & | 1.000 | & | 7.372 | \\ |
| $x_{120}$ | & | $ | R04953MM | $ | & | $ | x_{49},x_{99},x_{100},x_{119},x_{120},x_{123 | } | * | $ | & | 0.997 | & | 7.375 | \\ |
| $x_{121}$ | & | $ | R04954MM | $ | & | $ | x_{96},x_{101},x_{119},x_{121 | } | ** | $ | & | 1.000 | & | 7.375 | \\ |
| $x_{122}$ | & | $ | R04956MM | $ | & | $ | x_{97},x_{120},x_{122},x_{123 | } | * | $ | & | 1.000 | & | 7.375 | \\ |
| $x_{123}$ | & | $ | R04959MM | $ | & | $ | x_{99},x_{100},x_{101},x_{122},x_{123 | } | * | $ | & | 1.000 | & | 7.380 | \\ |
| $x_{124}$ | & | $ | R04968MM | $ | & | $ | x_{96},x_{101},x_{119},x_{124 | } | * | $ | & | 1.000 | & | 13.262 | \\ |
| $x_{125}$ | & | $ | R04970MM | $ | & | $ | x_{44},x_{102},x_{103},x_{122},x_{125 | } | * | $ | & | 0.999 | & | 13.300 | \\ |
| $x_{126}$ | & | $ | R05064MM | $ | & | $ | x_{126},x_{131 | } |  | $ | & | 1.000 | & | 0.000 | \\ |
| $x_{127}$ | & | $ | R05066MM | $ | & | $ | x_{127 | } |  | $ | & | 1.000 | & | 0.000 | \\ |
| $x_{128}$ | & | $ | R07162MM | $ | & | $ | x_{3},x_{43},x_{108},x_{112},x_{128 | } |  | $ | & | 1.000 | & | 0.048 | \\ |
| $x_{129}$ | & | $ | R07390MM | $ | & | $ | x_{9},x_{22},x_{26},x_{29},x_{30},x_{129 | } |  | $ | & | 1.000 | & | NaN | \\ |
| $x_{130}$ | & | $ | R07599MM | $ | & | $ | x_{9},x_{22},x_{26},x_{29},x_{30},x_{130 | } |  | $ | & | 1.000 | & | 0.000 | \\ |
| $x_{131}$ | & | $ | R07600MM | $ | & | $ | x_{9},x_{22},x_{26},x_{29},x_{30},x_{131 | } |  | $ | & | 1.000 | & | 0.000 | \\ |
| $x_{132}$ | & | $ | R07603MM | $ | & | $ | x_{67},x_{92},x_{132 | } | * | $ | & | 1.000 | & | 6.807 | \\ |
| $x_{133}$ | & | $ | R07604MM | $ | & | $ | x_{67},x_{92},x_{132},x_{133 | } | * | $ | & | 1.000 | & | 6.807 | \\ |
| $x_{134}$ | & | $ | R07618MM | $ | & | $ | x_{43},x_{66},x_{118},x_{134 | } |  | $ | & | 1.000 | & | 0.082 | \\ |
| $x_{135}$ | & | $ | R08157MM | $ | & | $ | x_{9},x_{22},x_{26},x_{29},x_{30},x_{135 | } |  | $ | & | 1.000 | & | 0.000 | \\ |
